# Ancient inland human dispersals from Myanmar into interior East Asia since the Late Pleistocene

**DOI:** 10.1038/srep09473

**Published:** 2015-03-26

**Authors:** Yu-Chun Li, Hua-Wei Wang, Jiao-Yang Tian, Li-Na Liu, Li-Qin Yang, Chun-Ling Zhu, Shi-Fang Wu, Qing-Peng Kong, Ya-Ping Zhang

**Affiliations:** 1 State Key Laboratory of Genetic Resources and Evolution, Kunming Institute of Zoology, Chinese Academy of Sciences, Kunming 650223, China; 2 KIZ/CUHK Joint Laboratory of Bioresources and Molecular Research in Common Diseases, Kunming, China; 3 Kunming College of Life Science, University of Chinese Academy of Sciences, Beijing 100049, China

## Abstract

Given the existence of plenty of river valleys connecting Southeast and East Asia, it is possible that some inland route(s) might have been adopted by the initial settlers to migrate into the interior of East Asia. Here we analyzed mitochondrial DNA (mtDNA) HVS variants of 845 newly collected individuals from 14 Myanmar populations and 5,907 published individuals from 115 populations from Myanmar and its surroundings. Enrichment of basal lineages with the highest genetic diversity in Myanmar suggests that Myanmar was likely one of the differentiation centers of the early modern humans. Intriguingly, some haplogroups were shared merely between Myanmar and southwestern China, hinting certain genetic connection between both regions. Further analyses revealed that such connection was in fact attributed to both recent gene flow and certain ancient dispersals from Myanmar to southwestern China during 25–10 kya, suggesting that, besides the coastal route, the early modern humans also adopted an inland dispersal route to populate the interior of East Asia.

It is generally agreed that the peopling of East Asia resulted mainly from the Late Pleistocene south-to-north migrations, initiated from Southeast Asia by the earliest settlers after they migrated from Africa *via* a coastal route at ~60 kilo-years ago (kya)^1^,^2^,^3^,^4^,^5^,^6^,^7^. However, so far it remains elusive how these initial settlers migrated into the interior of East Asia. Although it is plausible that modern humans kept adopting the coastal route and moved along the coastline of the ancient Sundaland, reaching and finally leading to the settlement of East Asia, an alternative possibility that the settlers might have adopted an inland route into the interior of East Asia *via* river valleys could not be ruled out. Actually, as suggested by GIS-based analysis, river valleys had likely played an important role in populating the interior of South Asia by modern humans^8^. This opinion echoes with the suggestion that the major river systems in the northern mainland Southeast Asia, such as Ayeyarwady, Salween, and Mekong, created diverse environments and paths for human dispersal and thus were of great help for early hominine adaptation^9^. The modern human cranium excavated from northern Laos, dated to 51–46 kya^10^, evinced the very early presence of modern humans in the interior of Southeast Asia and lent further support to this hypothesized inland dispersal scenario that likely occurred in the Late Pleistocene.

Unfortunately, so far no genetic trace of this inland dispersal(s) was observed, notwithstanding much progress has been achieved on dissecting the genetic landscapes in East Asians^2^,^3^,^7^,^11^,^12^,^13^,^14^ and Southeast Asians^7^,^15^,^16^,^17^,^22^. One possible reason is that only a few study took into account the genetic data from both East and Southeast Asian populations; more importantly, another reason may be attributable to the scarcity of genetic information from Myanmar, the largest country in mainland Southeast Asia which locates at the junction connecting South, Southeast and East Asia. Although Myanmar likely served as the corridor where the initial settlers had adopted to enter and colonize southeastern Asia during their migration along Asian coast^4^,^6^, previous studies focused either on the genetic structure of some ethnic populations^18^ or on the distribution of a single haplogroup (viz. M31) in Myanmar^19^.

Therefore, if this hypothesized ancient inland dispersal route did exist, Myanmar likely served as the corridor. In fact, the two major rivers (i.e. Ayeyarwady and Salween) in Myanmar can trace their upstream back to southwestern China. The existence of such river valleys would facilitate the potential population movement northwards into the interior zones. Coincidentally, our recent study has observed the enrichment of a number of new basal mtDNA lineages in southwestern China (especially Yunnan Province) and suggested this region likely to be the genetic reservoir of the modern humans after they entered East Asia^13^, further favoring the possibility of directly genetic contribution from Southeast Asia, say Myanmar, to southwestern China possibly occurred in the Late Pleistocene.

## Results

### Classification of mtDNA sequences in Myanmar populations

As shown in Supplementary Table S1 online, among the 845 Myanmar mtDNAs which were analyzed for their control-region and additional coding-region sites, the majority (532/845, 62.96%) could be allocated unambiguously into East Eurasian haplogroups, such as D, G, M7-M13, A, N9a, R9 and B^4^,^11^,^13^,^16^,^20^,^21^,^22^,^23^,^24^, whereas 4.26% (36/845) were assigned into haplogroups of South Asian ancestry^6^,^25^,^26^,^27^,^28^,^29^. Surprisingly, a high frequency of samples (269/845, 31.83%) could not be recognized based on control-region variation and partial coding-region information. Completely sequencing 64 representatives of these unrecognizable mtDNAs revealed that 225 of them belong in fact to certain sub-clades of the already defined haplogroups, e.g., M4, M5, M7, M20, M21, M24, M30, M33, M35, M45, M46, M49, M50, M51, M54, M55, M58, M60, M72, M76, M90, M91, R22, R31, N21 and HV (Supplementary Fig. S1 online); whereas the rest 44 mtDNAs were proven to represent 3 so far undefined basal lineages, for which could not find any sister clades after compared with over 20,666 mtDNA genomes worldwide (mtDNA tree Build 16^30^; http://www.phylotree.org/) and therefore were named as M82, M83 and M84 here (Supplementary Fig. S1 online).

### Genetic relationship of Myanmar populations and their surrounding groups

**Figure 1:**
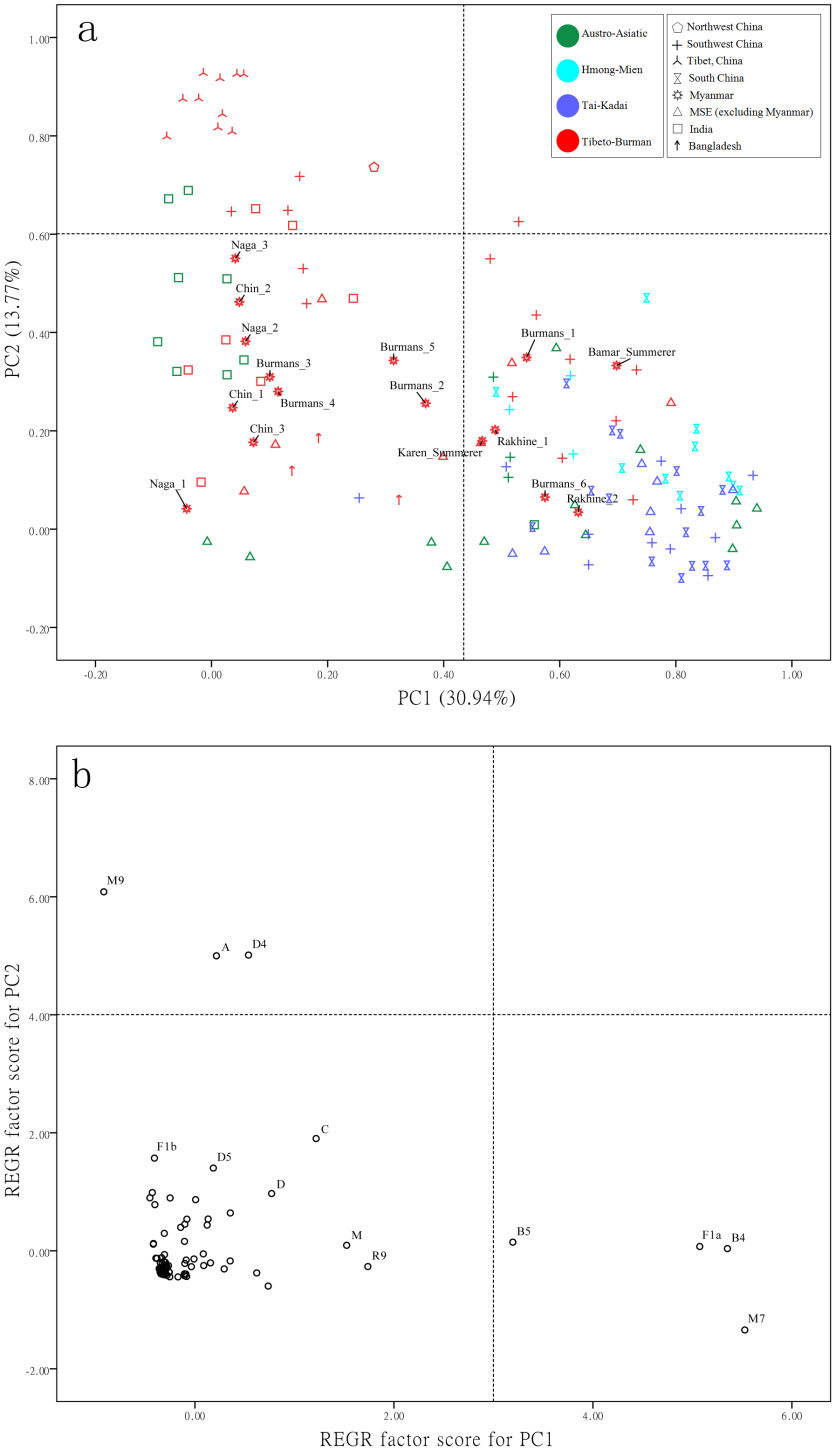
PCA of populations analyzed in the present study. (a) PC map of the 129 populations based on haplogroup frequencies. Myanmar populations were labeled. Barma_Summerer and Karen_Summerer were Barma and Karen from previous study^18^, for more details, see figure 4 and Supplementary Table S6 online. (b) Plot of the haplogroup contribution of the first and second PCs. The contribution of each haplogroup was calculated as the factor scores for PC1 and PC2 with regression (REGR) method in SPSS.

After unambiguously determining all the 845 Myanmar mtDNAs under study, the proportion of haplogroups of East Eurasian (66.51%) and South Asian (17.40%) ancestries remains stable in the whole Myanmar population. The East Asian-prevalent haplogroups (i.e., M9a, A, D4, G and C^2^,^11^,^14^,^20^,^21^,^31^) show relatively high frequencies in Myanmar populations (Supplementary Table S2 online), likely reflecting the genetic affiliation with the Tibeto-Burman precursors. The principal components analysis (PCA) result (accounting for 43.37% of the total variation) supports this scenario, as most of the Myanmar populations generally show closer affinity to Tibeto-Burman populations sampled from the other countries (Fig. 1a). This pattern finds further support from correspondence analysis (CA) (Supplementary Fig. S2 online) and the analysis of molecular variance (AMOVA) results (p < 0.05; Supplementary Table S3 online) based on haplogroup profiles.

### Genetic divergence in Myanmar populations

Nonetheless, substantial population substructure is still observed among Myanmar populations. In the PC map, Burmese (or Barma), Rakhine and Karen show closer relationship with Tai-Kadai and Hmong-Mien in Southeast Asia, while Naga and Chin show closer affinity to Austro-Asiatic and Tibeto-Burman populations in northeast India. This pattern plausibly reflects different population dynamics that the populations had suffered during their southward migration from East Asia and subsequently peopling scenarios^32^,^33^. In fact, Naga and Chin distribute in the border of Myanmar and northeast Indian^34^ and share many similarities of culture and language with populations in northeast India^35^ and thus likely obtained some genetic components from South Asia (Supplementary Tables S2 and S5 online). By contrast, Burmese (or Barma), Rakhine and Karen mainly distribute in Myanmar and would have less gene flow from northeast Indian populations than from Southeast Asian populations. This observation is consistent with previous suggestion that Burmese, Rakhine and Karen are typical Southeast Asian population rather than South Asian populations^18^, and gets further support from the admixture analysis in which the Tibeto-Burman populations in northeast India contributed more to both Naga and Chin than to the other Myanmar populations (Supplementary Table S4 online). Specifically, as the largest population in Myanmar, Burmese consist of 65% of the total population size of the country. Extensive genetic variation is observed among different Burmese regional populations, as reflected by the clustering pattern in PCA result (Fig. 1) as well as the haplogroup composition (Supplementary Table S1 online). Intriguingly, Burmese_1 and Burmese_6 show closer affinity to Rakhine and Karen populations, as well as Austro-Asiatic and Tai-Kadai populations from Southeast Asia, while Burmese_3 and Burmese_4 show closer affinity to northeast Indian and Bangladesh groups, likely reflecting gene flow between different Burmese and their neighboring populations.

### Enrichment of basal lineages in Myanmar populations

**Figure 2:**
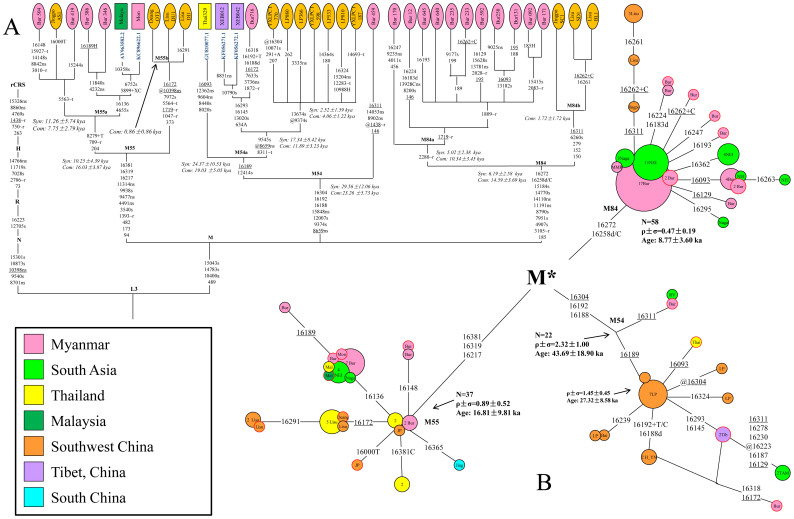
Phylogenetic trees and median networks of haplogroups M54, M55 and M84. Nucleotide position numbers are consistent with the revised Cambridge reference sequence (rCRS^54^). (a) Phylogenetic trees of haplogroups M54, M55 and M84. The newly sequenced samples in this study were marked in ellipses, while mtDNAs from the published literature were displayed in rectangles. Suffixes A, C and T refer to transversions, “d” means a deletion, and “+” indicates an insertion; recurrent mutations are underlined; “@” means a reverse mutation; “H” means heterogeneity. The C stretch length polymorphism in regions 303–315, AC indels at 515–522, 16182C, 16183C, 16193.1C(C) and 16519 were disregarded for the tree reconstruction. s, synonymous replacements; ns, nonsynonymous replacements; t, change in transfer RNA; r, change in ribosomal RNA gene; nc, mutations at the intergenic noncoding regions in segments 577–16023. Com: coalescent age calculated based on complete genome substitutions^51^; syn: coalescent age calculated based on coding region synonymous substitutions^51^. The geographic origin of samples was shown by different colors. (b) Median networks of haplogroups M54, M55 and M84 based mainly on HVS data (for more information, see Supplementary Table S8 online). The suffixes have the same meaning with those in the phylogenetic trees. The circles with red frame represent the complete sequenced individuals.

Intriguingly, even only Tibeto-Burman populations from Myanmar were analyzed in this study, which, as suggested in the historical records, could trace their origin back to western China^32^,^33^, a relatively high proportion of genetic components (15.38%) are proven to belong to the 3 newly identified basal lineages (viz. M82, M83 and M84) and some other basal lineages (viz. M24, M45, M49, M54, M55, M58, M72, M90, and M91 (Supplementary Table S1 online), which had been found in Myanmar and its surrounding areas according to the median joining networks (Fig. 2 and Supplementary Fig. S3 online) and previous studies^18^,^25^. This proportion retains stable (12.48%) after combining the published mtDNA data of Tibeto-Burman populations from Myanmar^18^. Specifically, with the exception of haplogroups M45, M72, M58 and M82 that show restricted distribution to a single ethnic group, the rest all are present in two or three ethnic populations. Significantly, out of the 12 basal lineages, 9 are observed in Burmese, whereas the number of these lineages is much fewer in the rest populations such as Naga (viz. M49, M55, M55, M24 and M83), Chin (viz. M49, M55, M72 and M84), Rakhine (or Arakanese) (viz. M24, M49, M82, M83 and M84), Da Wai (viz. M91), and Karen (viz. M90). It is possible that most of these lineages observed in Burmese are simply attributed to the largest sample size of this population analyzed in the present study. However, in consideration of the fact that Burmese is the largest population in Myanmar and consists of more than a half of the total sample of the country, our observation raises an alternative possibility that substantially genetic components from the aboriginal people had been assimilated into Burmese during the formation of this population. This notion is further supported by the small genetic distance between different Burmese and their surrounding populations from different geographic areas (Supplementary Table S5 online) and the scattered distribution of Burmese according to the PC analysis (Fig. 1a), as well as more genetic contribution of Austro-Asiatic and Tai-Kadai populations from Southeast Asia or southern East Asia to Burmese, compared with Naga and Chin (Supplementary Table S4 online).

Since these basal lineages likely represent the genetic relict of the initial settlers in Southeast Asia, the trace of the suggested Pleistocene inland dispersal, if really existed, would then be witnessed by these lineages. Meanwhile, the observed high diversity of the basal lineages in Myanmar seems to echo with the previous observation in southwestern China^13^, emphasizing the necessity for detailed analysis for these basal lineages. To achieve that, we further collected of 5,907 reported mtDNA sequences covering 115 populations from the neighboring regions of Myanmar (Supplementary Table S6 online), with especial attention to detect any possible ancient connection between Myanmar and the other region, especially southwestern China.

**Figure 3:**
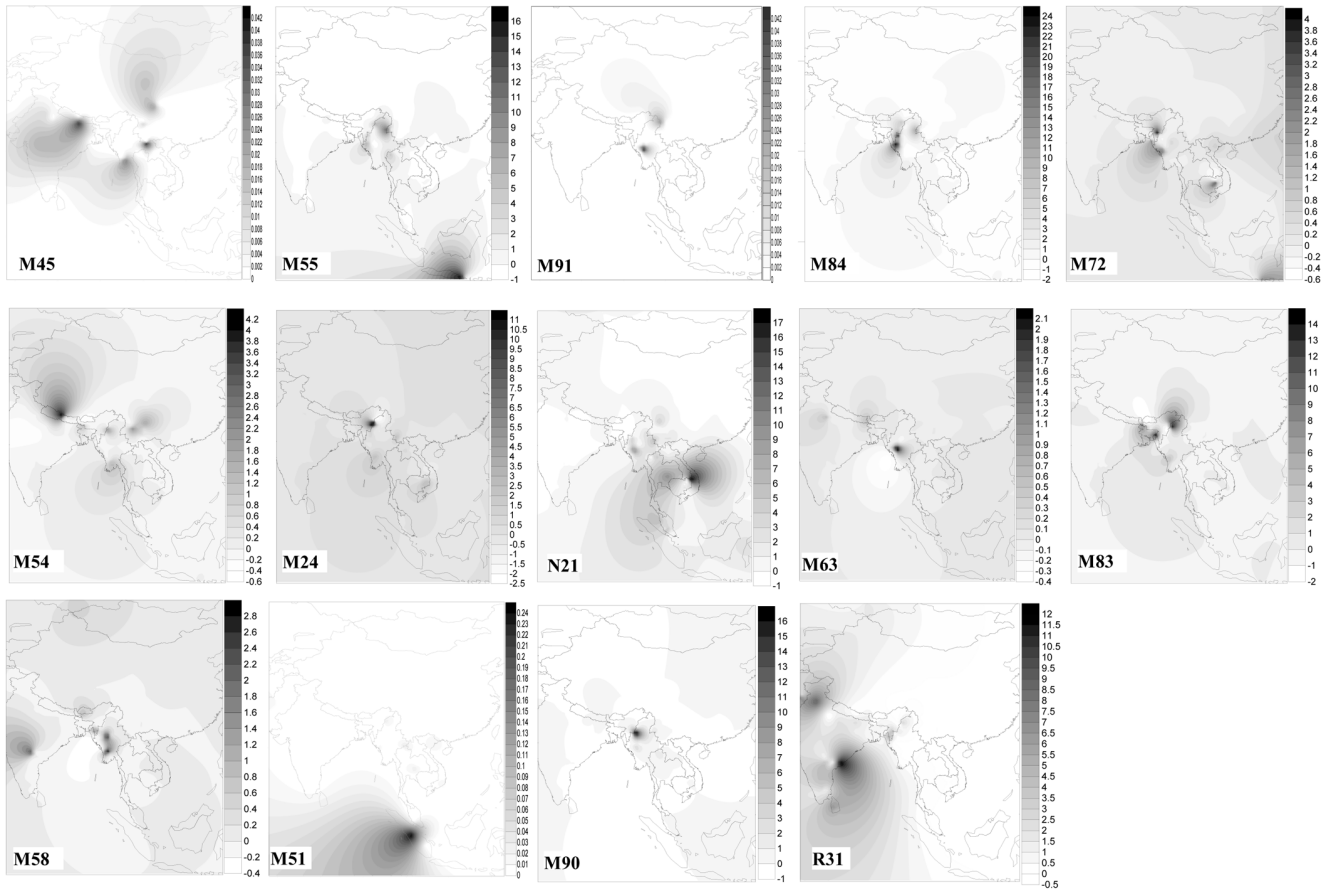
Contour maps of Myanmar basal haplogroups. These spatial-frequency distributions were created using the Kriging linear model of the Surfer 8.0 package, based on the frequency of each haplogroup in different populations (Supplementary Table S2 online).

By searching these basal haplogroups, we did observe their presence in the other neighboring populations, and their affinity was confirmed after the haplogroup-specific mutations were genotyped in the samples with matched control-region motif. Among the basal lineages in Myanmar, haplogroups M49, M72, M83, M55, M90, M91, M54, M84 and M24 show restricted distribution and high diversity in Myanmar and its surrounding areas, such as northeast India, northern Thailand, northern Laos and southwestern China (Figs. 2, 3 and Supplementary Fig. S3 online), suggesting their origin and differentiation *in situ*. Indeed, by showing the positive significant Moran's I values for small geographic distances and negative significant values for large distance classes (Supplementary Fig. S4 online), spatial analysis indicates clinal distributions of the haplogroups. These observations seem to be consistent with the crucially geographical location of Myanmar by connecting South, Southeast and East Asia. Time estimation results reveal that most of these basal haplogroups have ages as old as 50–20 kya (Table 1). The exceptions are haplogroups M55, M72 and M84, which coalesce ~15–9. The possible explanation of their recent coalescent ages would be that these haplogroups had gone through a bottleneck event or a founder effect during this period.

### Haplogroup sharing between Myanmar and southwestern China

Intriguingly, some haplogroups (including M90, M91, M24, M55, M54 and M84) are also found in southwestern China (Fig. 2 and Supplementary Fig. S3, Table S2 online). An extensive searching in large-scale of HVS data of over 47,000 individuals collected from Asia confirms their restricted distribution in Myanmar and southwestern China (Supplementary Table S7 online). To shed light on the mechanism underlying this distribution pattern, further analyses, including median networks and complete mtDNA sequencing, were performed on these haplogroups. As shown in median networks (Supplementary Fig. S3b online) and the reconstructed phylogenetic trees based on complete mitochondrial genomes (Supplementary Fig. S3a online), haplogroups M24, M90 and M91, with the ancestral nodes mainly occupied by Myanmar individuals, have only few haplotypes observed sporadically in southwestern China, reflecting their origination in Myanmar and recent gene flow into southwestern China. In contrast, the situation of haplogroups M54, M55 and M84 are somewhat different, as they have root types either in Myanmar and northeast India (M54 and M84; Fig. 2b), or at the border of Myanmar and Thailand (M55; Fig. 2b), suggesting these haplogroups possibly to be originated in Myanmar and its surrounding regions. Intriguingly, some subclades of these haplogroups are found to have confined distribution and thus *de novo* differentiation in southwestern China (Fig. 2), which is likely to be the results of ancient genetic contribution from Myanmar. Specifically, one branch of M55, which is defined by 16172, 7972, 5564, 1719, 1047, 373 and a recurrent mutation at position 10398 and designated as M55b here (Fig. 2a), is observed mainly in southwestern China and Thailand, indicating genetic introgression from Myanmar into these two places. Similarly, M84b, defined by 16311, 6260, 279, 152 and 150 is only observed in Yunnan, China (Fig. 2a). Haplogroup M54a, which is defined by 12414 and 16189, has a star-like structure with its root types occupied only by individuals from Yunnan, while the terminal haplotypes distributed in Yunnan, Tibet, Thailand and even Myanmar (Fig. 2a), likely reflecting an ancient demographic expansion.

## Discussion

In this report, by extensively dissecting the matrilineal composition in Myanmar populations (comprising Burmese, Chin, Rakhine, and Naga) at a high molecular resolution, our results show that more than a half of their maternal components belong to previously defined eastern Eurasian haplogroups, an observation in agreement with their linguistic affinity (Tibeto-Burman) and the historical records^32^,^33^. The relatively low distribution frequencies of South Asian lineages, such as M4, M5, M30, M33 and M45, suggest limited genetic introgression from India. These results were in concordance with previous suggestion that the Myanmar populations (especially Barma, Karen and Rakhine) showed similarity with populations in Southeast Asia, rather than South Asia^18^.

Significantly, our study has identified a number of novel and basal lineages in Myanmar, notwithstanding the fact that the Myanmar populations under consideration all have suggested Tibeto-Burman ancestry. Given the ancient coalescent ages (during Upper Pleistocene; Table 1) of these lineages as well as their restricted distribution and the highest diversities in Myanmar and neighboring regions (Figs. 2, 3 and Supplementary Fig. S3 online), it is then most likely that this region likely served as the one of the genetic differentiation centers of the initial settlers after they reached mainland Southeast Asia^36^.

Among the basal lineages identified in the Myanmar populations, some (e.g. M24, M90, M91, M55, M54 and M84) are also observed in southwestern China, suggesting certain direct but previously unknown genetic connections between Myanmar and mainland China. Further analyses reveal that haplogroups M24, M90 and M91 in southwestern China were the results of recent gene flow from Myanmar, likely occurred during the expansion of Pyu populations at about 200 BCE^32^ or economic trade between Yunnan and Myanmar (and northeast India) since Qin and Han dynasties^37^. Intriguingly, haplogroups M54, M55 and M84, showing the highest genetic diversity and thus their origination in the border of Myanmar and northeast India (e.g. M54 and M84), or the border of Myanmar and Thailand (e.g. M55), have subclades (i.e. M54a, M55b and M84b) to be present merely in southwestern China, strongly arguing for the existence of ancient genetic connection between both regions and, furthermore, suggesting that this connection was attributed to human dispersal(s) from Myanmar to the interior of China. The estimated ages of haplogroups M54a, M55b and M84b fall in two time periods (viz. ~20 vs. ~10 kya; Table 1), suggesting the migration events might last from the Late Paleolithic to early Neolithic.

This genetic connection finds additional support from the archaeological records. For instance, the carved stones, mainly observed in Paleolithic cultures in Yunnan of China, have also been found (although with a small proportion) in Anyathian sites near the border of Yunnan and Myanmar^38^. Additionally, the Neolithic cultures in the west of Shan plateau, like the Padah-lin Caves (13,000–1,700 years before present^39^), were suggested to have similarity with their stone counterparts in their surrounding areas, including western Yunnan^40^. The late Anyathian cultures in Myanmar were proved to distribute mainly along the Ayeyarwady river valley^41^, adding further support to the notion that the early hunter-gathers had moved along the major river valleys into the interior of East Asia.

In summary, the enrichment of a number of basal lineages in the Myanmar population indicates that this region likely served as one of the differentiation centers of the initial settlers after they reached southeastern Asia. Intriguingly, consistent with their geographic affinity between Myanmar and southwestern China, direct genetic connection exist between both regions which was attributed to recent gene flow from Myanmar to southwestern China (witnessed by haplogroups M24, M90 and M91) and certain ancient dispersals from Myanmar to southwestern China ranged from 25–10 kya (manifested by haplogroups M54, M55 and M84). This observation finds further support from the archaeological records and well explains the enrichment of basal lineages in southwestern China observed in our previous study^13^. As such, these findings disclose that, besides the well-known coastal route, the inland dispersal route did exist and was adopted by the ancestors of modern humans to enter and populate the interior of mainland East Asia, which was most likely facilitated by river valleys and had played an important yet unrecognized role in peopling East Asia.

## Methods

### Sampling

**Figure 4:**
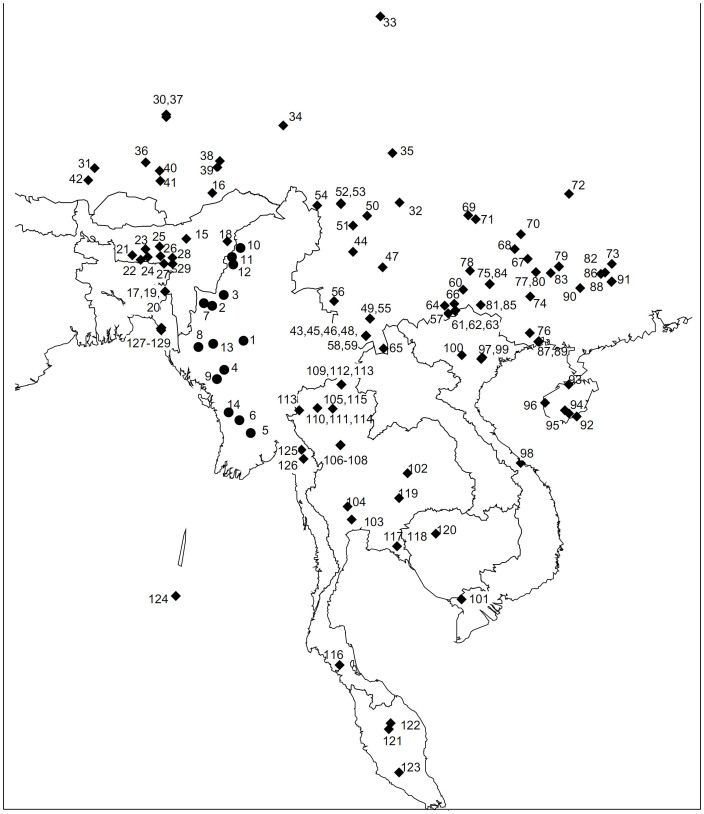
Geographic locations of 129 populations analyzed in the present study. The 14 Myanmar populations collected in this study are indicated by solid circles, whereas the rest populations are indicated by diamonds. The map was created by Surfer 8.0 package. More details regarding the populations are displayed in Supplementary Table S6 online.

To dissect the matrilineal landscape in Myanmar, a total of 845 individuals, sampled from 14 Myanmar populations representing the major ethnic groups in the country including Burmese, Chin, Rakhine, and Naga, were considered in the current investigation. The experimatal protocol were approved by the Ethics Committee at Kunming Institute of Zoology. Informed consent was obtained from each individuals before the study. The relevant information of the ethnic populations as well as the sample locations were summarized in Table S6 (Supplementary Table S6 online) and Figure 4, respectively.

### Sequencing and haplogroup allocation

Procedures for the extraction of genomic DNA, PCR amplification and DNA sequencing of mtDNA segment(s) have already been well described elsewhere^2^,^12^,^42^. The control-region sequencing ranges for the samples were listed in Table S1(Supplementary Table S1 online), commonly covering region 16024–407. Coding region RFLPs, including 8281–8289 deletion, 4831*Hha*I, 5176*Alu*I, 9820*Hinf*I, 12406*Hpa*I, 13262*Alu*I, 14465*Acc*I, were tested following the methods of Yao et al. (2004)^42^. All samples were first assigned into respective haplogroups on the basis of combined control-region and partial coding-region information according to the reconstructed mtDNA tree of East Asian^11^,^21^, Southeast Asian^4^,^15^,^22^,^23^, South Asian^6^,^25^,^26^,^27^,^28^,^29^, and even European lineages^6^,^43^,^44^,^45^. For the rest unassigned mtDNAs, complete genome sequencing, complemented by motif-search strategy^11^, was carried out, with the special intention to identify all the possible existed basal lineages in the samples with minimum effort. Specifically, 64 representatives of the unassigned mtDNAs were selected on the basis of the control-region variation motif for complete mtDNA genome sequencing (Supplementary Table S1 online). In detail, for samples from the same ethnic group and with identical control-region variation motif, one representative was chosen for complete sequencing. Then, the phylogenetic status of the remaining mtDNAs were determined by typing some specific coding-region sites (Supplementary Table S1 online) which were chosen under the guideline of newly obtained mtDNA genome information (Supplementary Fig. S1 online). For any mtDNA whose phylogenetic status could not be identified yet, further complete sequencing work will be carried out. When naming the newly identified novel basal lineages, we followed the nomenclature listed in PhyloTree website (mtDNA tree Build 16^30^; http://www.phylotree.org/) and our recently suggested haplogroup scheme^46^. For haplogroups of interest, 28 additional representative samples were also chosen for complete sequencing. The experiments were carried out in accordance with the approved guideline of Chinese Academy of Sciences.

### Data quality control

To ensure the quality of the complete genome data, our previously proposed quality-control measures, such as independent amplification, detecting errors by phylogenetic analysis and matching or near-matching method were followed^6^,^11^,^28^. Furthermore, to avoid the amplification of NUMT^47^ as well as the problem of artificial recombination that is easily introduced when dozens of primer sets are involved^48^, four pairs of PCR primers were designed to amplify the whole mtDNA genome. Each fragment was amplified independently. The amplified fragments, each of which contains more than 400bp overlapping regions with its neighbors^49^, were sequenced by use of 48 inner primers (12 for each fragment) reported in our previous studies^6^,^20^.

### Data analyses

To facilitate the comparison with the reported data from the neighboring populations and thus to distill the genetic trace left by the Pleistocene inland immigrants, previously published mtDNA data, including 1,524 mtDNAs from mainland Southeast Asia and 3,120 mtDNAs from East Asia, 690 mtDNA sequences from northeast India and 302 from Bangladesh, were taken into account. In addition, the recently published Myanmar data (116 Barma and 155 Karen^18^) were also considered (Fig. 4 and Supplementary Table S6 online).

The PCA and CA were performed based on haplogroup frequencies by using Statistical Package for the Social Sciences (SPSS) 16.0 software. Reduced median network for each basal haplogroup was constructed manually and checked by the program NETWORK 4.510 (www.fluxus-engineering.com/sharenet.htm). Phylogenetic tree of haplogroup was reconstructed manually based on complete sequences and confirmed by mtphyl software. Contour maps of spatial frequencies of haplogroup were constructed using Kriging linear model of Surfer 8.0 (Golden Software Inc. Golden, Colorado, USA). Spatial analysis was performed using the PASSAGE software packet. Moran's I metrix was applied in correlogram analysis^50^. The time to the most recent common ancestor (TMRCA) of a haplogroup was estimated using ρ statistic method as described previously^51^. Nei's d_A_ genetic distances^52^ and AMOVA were calculated by using the package Arlequin 3.11. Admixture estimation was performed by the Weighted Least Squares (WLS) Method^53^ using SPSS 16.0.

## Author Contributions

Q.-P.K. and Y.-P.Z. designed the research; L.-N.L., L.-Q.Y., C.-L.Z. and S.-F.W. colleted the samples; Y.-C.L., H.-W.W. and J.-Y.T. collected the data; Y.-C.L., H.-W.W., J.-Y.T. and C.-L.Z. performed the experiments; Y.-C.L., H.-W.W., J.-Y.T., Q.-P.K. and Y.-P.Z. analyzed data; Y.-C.L., Q.-P.K. and Y.-P.Z. wrote the paper.

## Additional Information

**Accession numbers:** All of the sequences obtained in the present study have been deposited into GenBank, with accession numbers KP345975-KP346066 for whole mtDNA genomes and KP346067-KP346911 for control region sequences.

## Supplementary Material

Supplementary Information Supplementary information

Supplementary Information Dataset 1

Supplementary Information Dataset 2

## Figures and Tables

**Table 1:** Estimated ages for basal haplogroups of Myanmar

		Complete genome substitutions§		Coding region synonymous substitutions†			Transitions between 16090 and 16365‡	
Haplogroup	N.	ρ ± σ	Age (kya)	ρ ± σ	Age (kya)	N.	ρ ± σ	Age (kya)
M24	9	7.89 ± 1.64	20.39 ± 4.23	2.78 ± 1.07	21.90 ± 8.45	37	0.84 ± 0.73	15.79 ± 13.84
M45	16	9.50 ± 1.47	24.56 ± 3.79	3.88 ± 0.82	30.55 ± 6.44	19	2.32 ± 0.96	43.64 ± 18.02
M54	12	9.00 ± 2.22	23.26 ± 5.73	3.75 ± 1.53	29.56 ± 12.06	22	2.32 ± 1.00	43.69 ± 18.90
M54a	11	7.36 ± 1.95	19.03 ± 5.05	3.09 ± 1.33	24.37 ± 10.53	20	1.45 ± 0.45	27.32 ± 8.58
M49	32	9.19 ± 1.52	23.75 ± 3.93	3.00 ± 0.77	23.65 ± 6.07	-	-	-
M55	10	6.20 ± 1.50	16.03 ± 3.87	1.30 ± 0.56	10.25 ± 4.39	37	0.89 ± 0.52	16.81 ± 9.81
M55a	7	3.00 ± 1.08	7.75 ± 2.79	1.43 ± 0.73	11.26 ± 5.74	-	-	-
M55b	3	0.33 ± 0.33	0.86 ± 0.86	-	-	-	-	-
M58	4	17.00 ± 2.62	43.94 ± 6.78	3.75 ± 1.25	29.56 ± 9.85	11	4.73 ± 1.44	89.09 ± 27.09
M72	6	5.83 ± 1.26	15.08 ± 3.25	1.17 ± 0.60	9.20 ± 4.74	22	1.54 ± 0.57	29.12 ± 10.70
M83	5	13.40 ± 1.99	34.64 ± 5.14	5.80 ± 1.25	45.73 ± 9.85	40	1.00 ± 0.38	18.84 ± 7.21
M84	14	5.64 ± 1.43	14.59 ± 3.69	0.79 ± 0.33	6.19 ± 2.58	58	0.47 ± 0.19	8.77 ± 3.60
M84a	11	4.00 ± 1.34	10.34 ± 3.45	0.64 ± 0.30	5.02 ± 2.38	-	-	-
M84b	3	0.67 ± 0.67	1.72 ± 1.72	-	-	-	-	-
M90	7	11.29 ± 2.26	29.17 ± 5.85	4.14 ± 1.41	32.66 ± 11.09	27	0.26 ± 0.10	4.89 ± 1.85
M91	10	15.30 ± 2.45	39.55 ± 6.33	7.70 ± 1.88	60.71 ± 14.85	17	1.88 ± 0.91	35.47 ± 17.17
R31	9	21.78 ± 2.56	56.30 ± 6.61	8.22 ± 1.60	64.82 ± 12.63	74	3.76 ± 0.95	70.80 ± 17.84
M51	24	12.33 ± 2.01	31.88 ± 5.18	3.08 ± 0.75	24.31 ± 5.91	25	1.88 ± 0.68	35.43 ± 12.72
M63	8	9.88 ± 2.35	25.53 ± 6.07	5.50 ± 1.78	43.36 ± 14.01	13	1.46 ± 0.77	27.55 ± 14.42
N21	14	6.50 ± 1.46	16.80 ± 3.77	1.93 ± 0.63	15.20 ± 5.01	122	1.50 ± 0.59	28.11 ± 11.12

N: Number of mtDNA sequences.

^§^Mutation rate is one mutation per 2,585 years^51^;

^†^Mutation rate is one mutation per 7,884 years^51^;

^‡^Mutation rate is one mutation per 18,845 years^51^.
