# Supplementary material for: Ancient inland human dispersals from Myanmar into interior East Asia since the Late Pleistocene
Source: Sci Rep. 2015 Mar 26;5:9473. doi: 10.1038/srep09473 (PMC4379912; doi:10.1038/srep09473)
Supplement: Supplementary Information [file srep09473-s1.pdf]

# Supplementary Information

## Ancient inland human dispersals from Myanmar into interior East Asia since the Late Pleistocene

Yu-Chun Li, Hua-Wei Wang, Jiao-Yang Tian, Li-Na Liu, Li-Qin Yang, Chun-Ling Zhu, Shi-Fang Wu, Qing-Peng Kong, and Ya-Ping Zhang

**Figure S1. Reconstructed phylogenetic tree of Myanmar mtDNA lineages based on 64 complete sequences**. Nucleotide position numbers are consistent with the revised Cambridge reference sequence (rCRS[1]). Suffixes A, C, G, and T refer to transversions, ‘‘d’’ means a deletion, and ‘‘+’’ indicates an insertion; recurrent mutations are underlined; ‘‘@’’ means a reverse mutation; “H” means heterogeneity. The C stretch length polymorphism in regions 303–315, AC indels at 515-522, 16182C, 16183C, 16193.1C(C) and 16519 were disregarded for the tree reconstruction.

Reference for Figure S1

1. Andrews RM, *et al*. Reanalysis and revision of the Cambridge reference sequence for human mitochondrial DNA. *Nat. Genet.* **23,** 147 (1999).

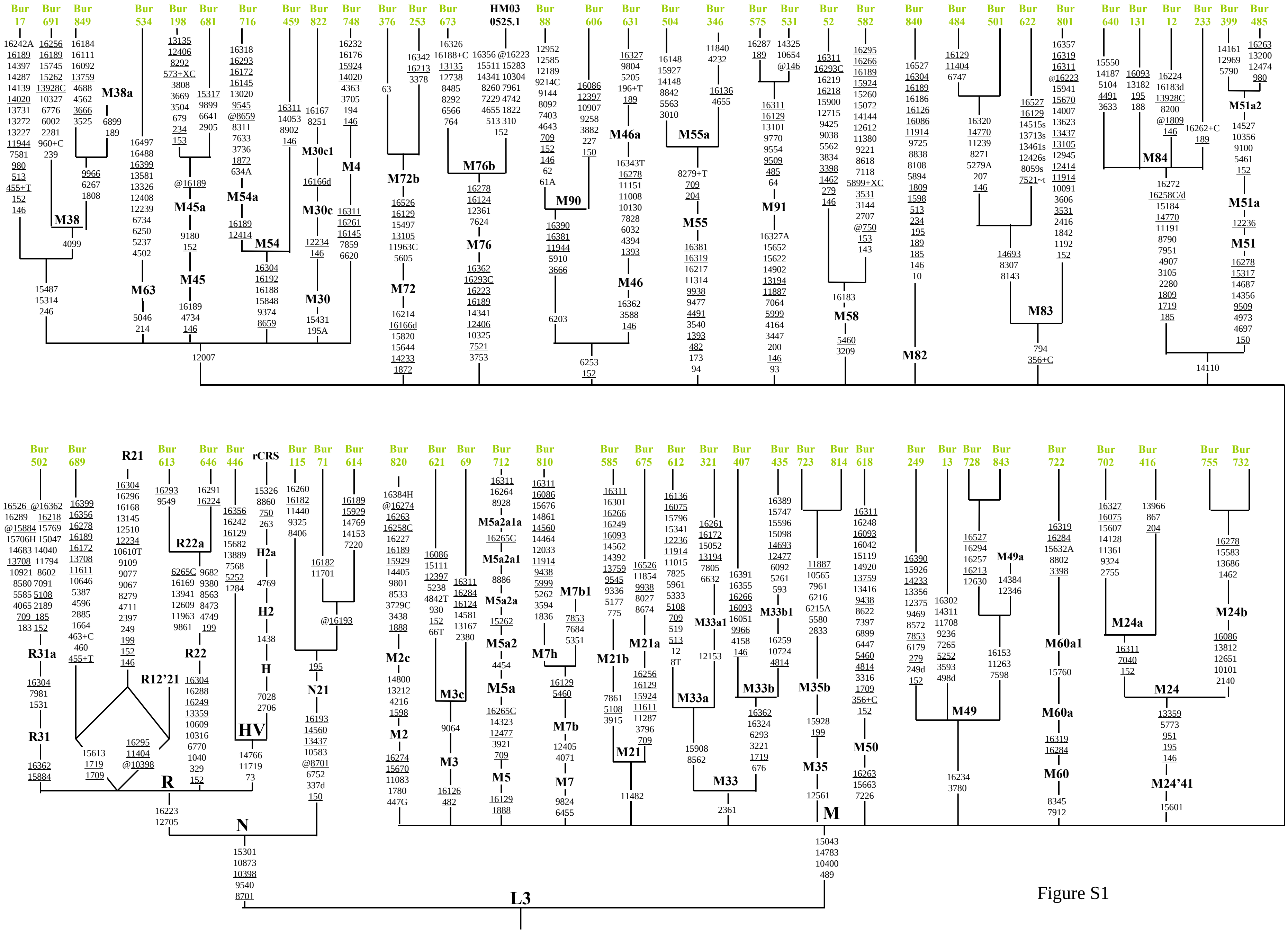
Bur 17
Bur 691
Bur 849
Bur 534
Bur 198
Bur 681
Bur 716
Bur 459
Bur 822
Bur 748
Bur 376
Bur 253
Bur 673
HM03 0525.1
Bur 88
Bur 606
Bur 631
Bur 504
Bur 346
Bur 575
Bur 531
Bur 52
Bur 582
Bur 840
Bur 484
Bur 501
Bur 622
Bur 801
Bur 640
Bur 131
Bur 12
Bur 233
Bur 399
Bur 485
M38a
M38
M63
M45a
M45
M54a
M54
M30c1
M30c
M30
M4
M72b
M72
M76b
M76
M90
M46a
M46
M55a
M55
M91
M58
M82
M83
M84
M51a2
M51a
M51
Bur 502
Bur 689
R21
Bur 613
Bur 646
Bur 446
rCRS
Bur 115
Bur 71
Bur 614
Bur 820
Bur 621
Bur 69
Bur 712
Bur 810
Bur 585
Bur 675
Bur 612
Bur 321
Bur 407
Bur 435
Bur 723
Bur 814
Bur 618
Bur 249
Bur 13
Bur 728
Bur 843
Bur 722
Bur 702
Bur 416
Bur 755
Bur 732
R22a
R22
R31a
R31
R12’21
R
HV
H2a
H2
H
N21
N
M2c
M2
M3c
M3
M5a2a1a
M5a2a1
M5a2a
M5a2
M5a
M5
M7b1
M7h
M7b
M7
M21b
M21a
M21
M33a1
M33a
M33b1
M33b
M33
M35b
M35
M
M50
M49a
M49
M60a1
M60a
M60
M24a
M24b
M24
M24’41
L3

Figure S1

**Figure S2. Two-dimension plot of the correspondence analysis based on haplogroup frequencies.** Only the haplogroups (represented by gray circles) contributing most to the first and the second dimensions are labelled. Populations from Myanmar were labelled as numbers 1-16. 1, Burmans_1; 2, Burmans_2; 3, Burmans_3; 4, Burmans_4; 5, Burmans_5; 6, Burmans_6; 7, Naga_1; 8, Naga_2; 9, Naga_3; 10, Chin_1; 11, Chin_2; 12, Chin_3; 13, Rakhine_1; 14, Rakhine_2; 15, Bamar_Summerer[1]; 16, Karen_Summerer[1].

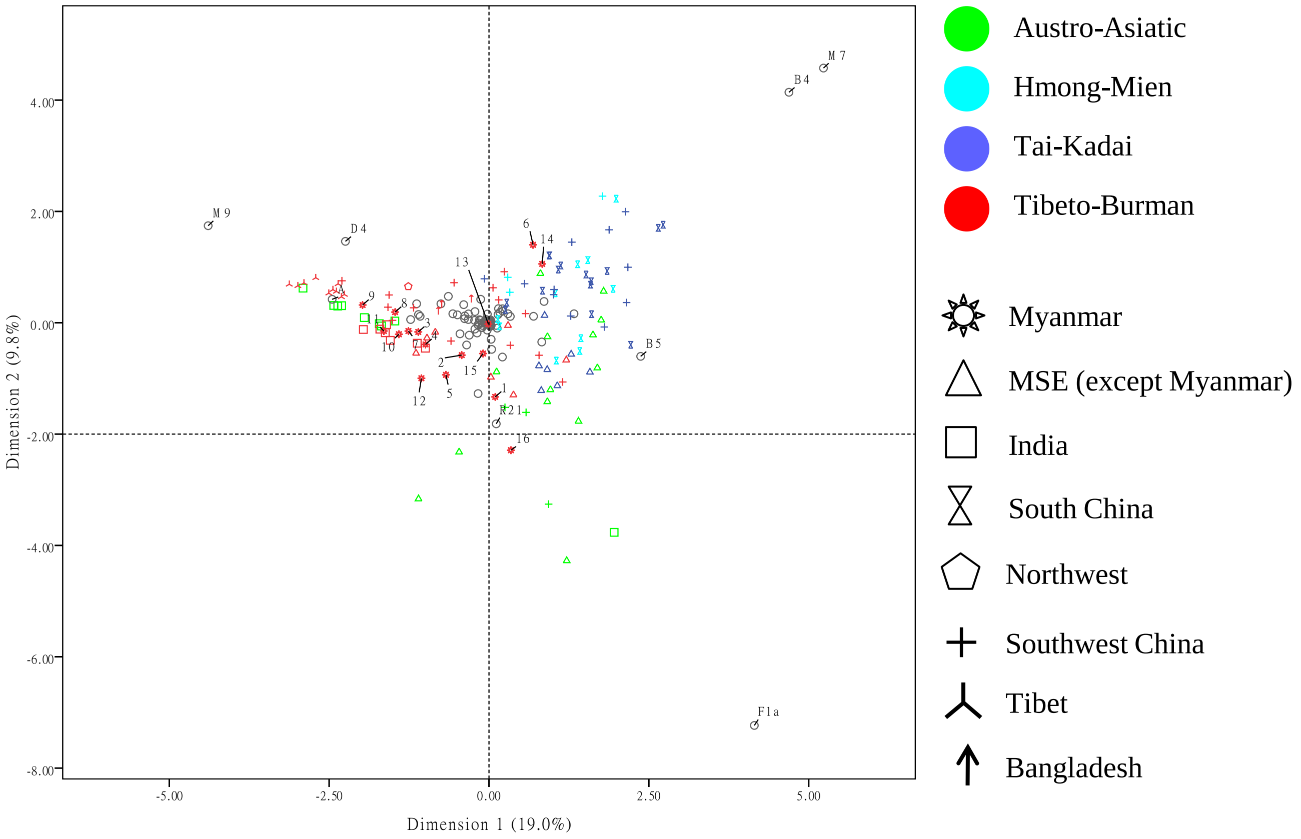



Reference for Figure S2

1. Summerer M, *et al.* Large-scale mitochondrial DNA analysis in Southeast Asia reveals evolutionary effects of cultural isolation in the multi-ethnic population of Myanmar. *BMC Evol. Biol.* **14,** 17 (2014).

**Figure S3.** Phylogenetic trees and median networks of M24, M45, M49, M58, M63, M72, M83, M90 and M91. (a). Phylogenetic trees of M24, M45, M49, M58, M63, M72, M83, M90 and M91. (b). median networks of M24, M45, M58, M63, M72, M83, M90 and M91. based mainly on HVS data (for more information, see Table S8). See the legends of Figures S1 and 3 for more details.

# Fig S3a

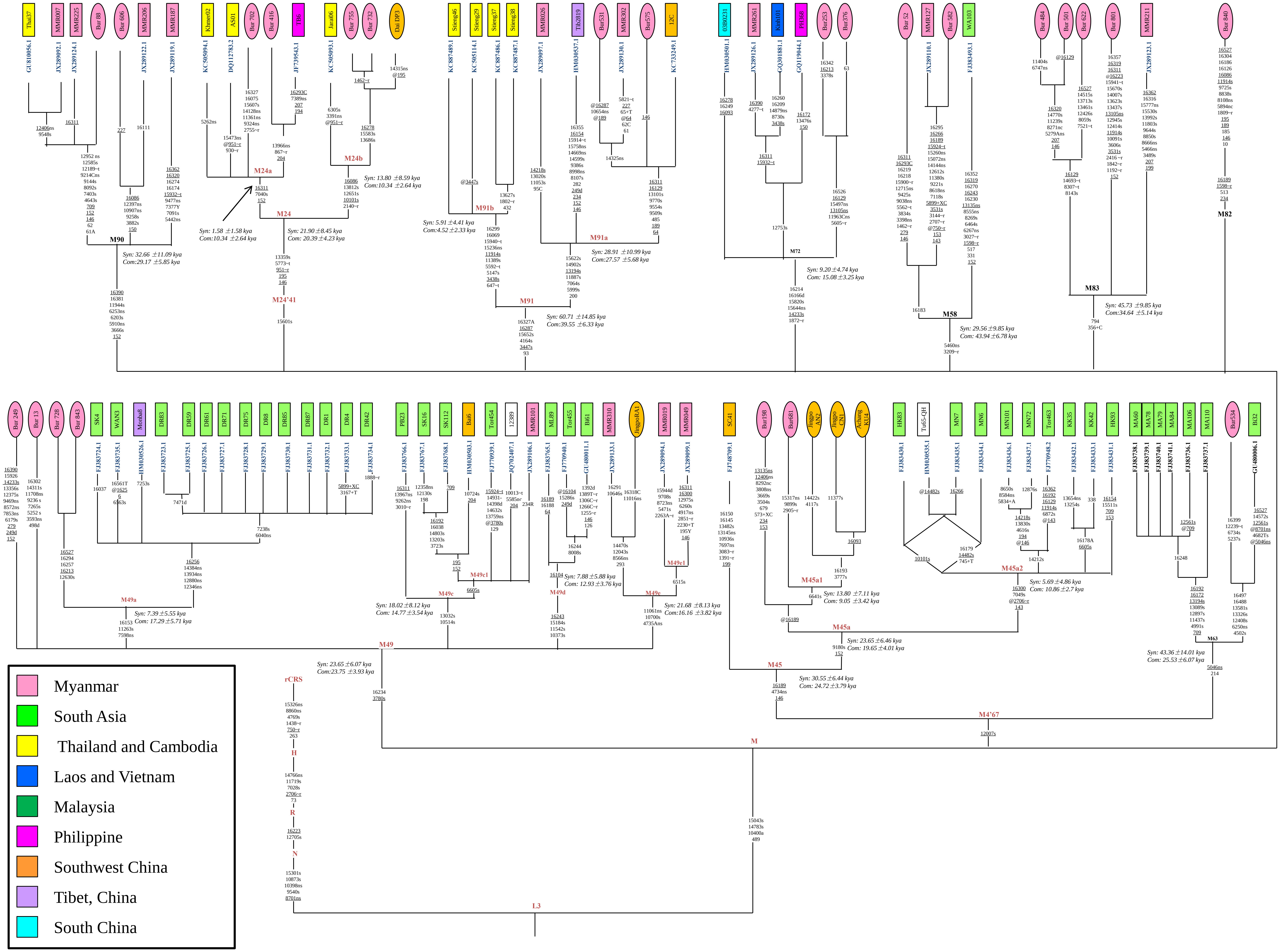
Myanmar
South Asia
Thailand and Cambodia
Laos and Vietnam
Malaysia
Philippine
Southwest China
Tibet, China
South China
M90
M24a
M24b
M24
M24'41
M91b
M91a
M91
M72
M58
M83
M82
M49a
M49c1
M49c
M49d
M49e1
M49e
M49
M45a1
M45a2
M45a
M45
M63
M4'67
M
rCRS
H
R
N
L3

Fig S3b

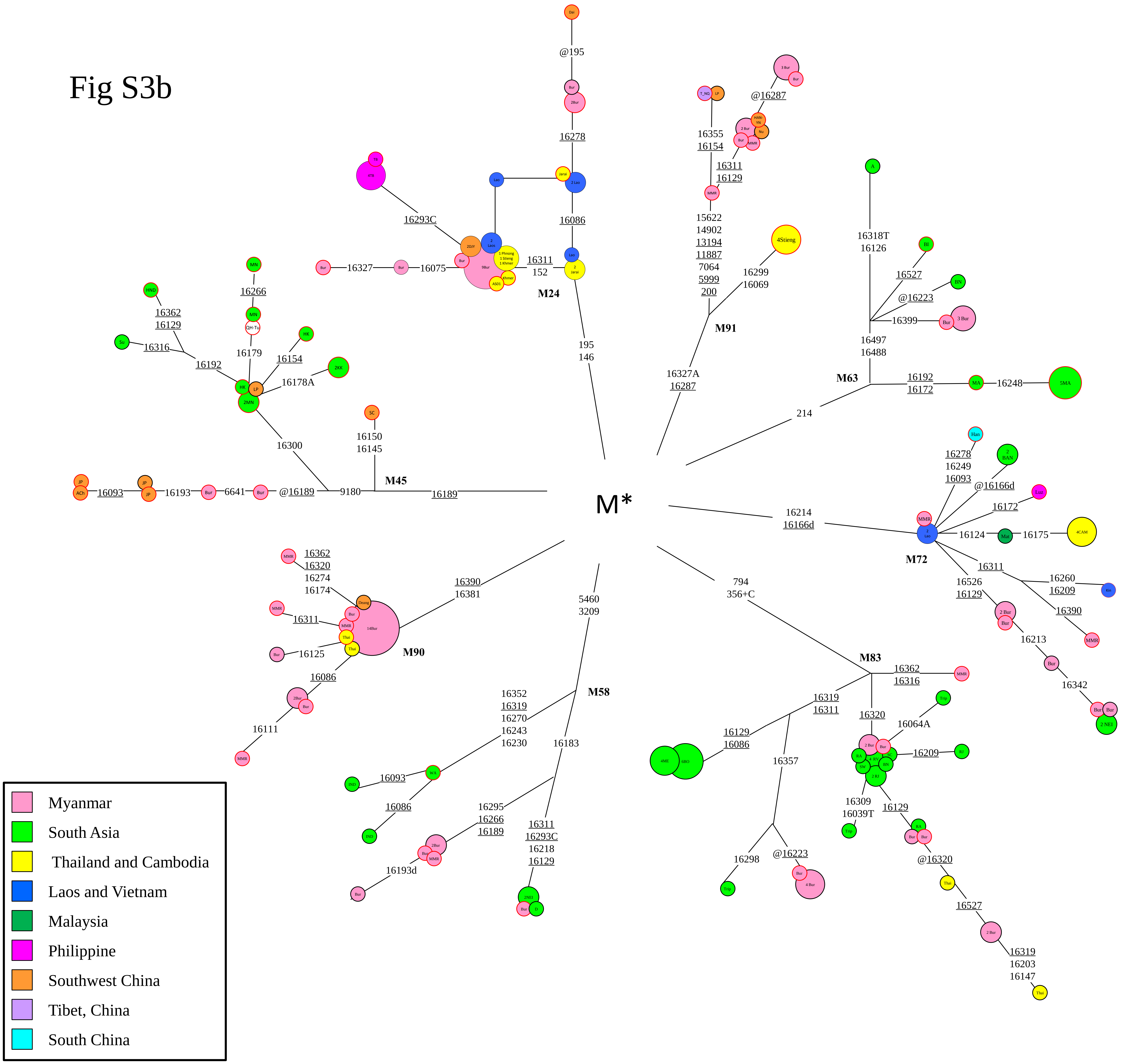
M*
M24
@195
16278
16086
16311
152
195
146
16293C
16327
16075
M45
16189
9180
@16189
6641
16193
16093
16150
16145
16300
16178A
16154
16179
16266
16192
16316
16362
16129
M91
16355
16154
16311
16129
15622
14902
13194
11887
7064
5999
200
16299
16069
16327A
16287
@16287
4Stieng
M63
16318T
16126
16527
@16223
16399
16497
16488
16192
16172
16248
214
5MA
M72
16214
16166d
16278
16249
16093
@16166d
16172
16124
16175
4CAM
16311
16260
16209
16390
16526
16129
16213
16342
M83
794
356+C
16362
16316
16319
16311
16320
16064A
16209
16129
16086
16357
16309
16039T
16129
16298
@16223
@16320
16527
16319
16203
16147
M58
5460
3209
16352
16319
16270
16243
16230
16183
16093
16086
16295
16266
16189
16193d
16311
16293C
16218
16129
M90
16390
16381
16362
16320
16274
16174
16311
16125
16086
16111
14Bur
Myanmar
South Asia
Thailand and Cambodia
Laos and Vietnam
Malaysia
Philippine
Southwest China
Tibet, China
South China

**Figure S4. Spatial Analyses Correlograms of different haplogroups in Myanmar.**

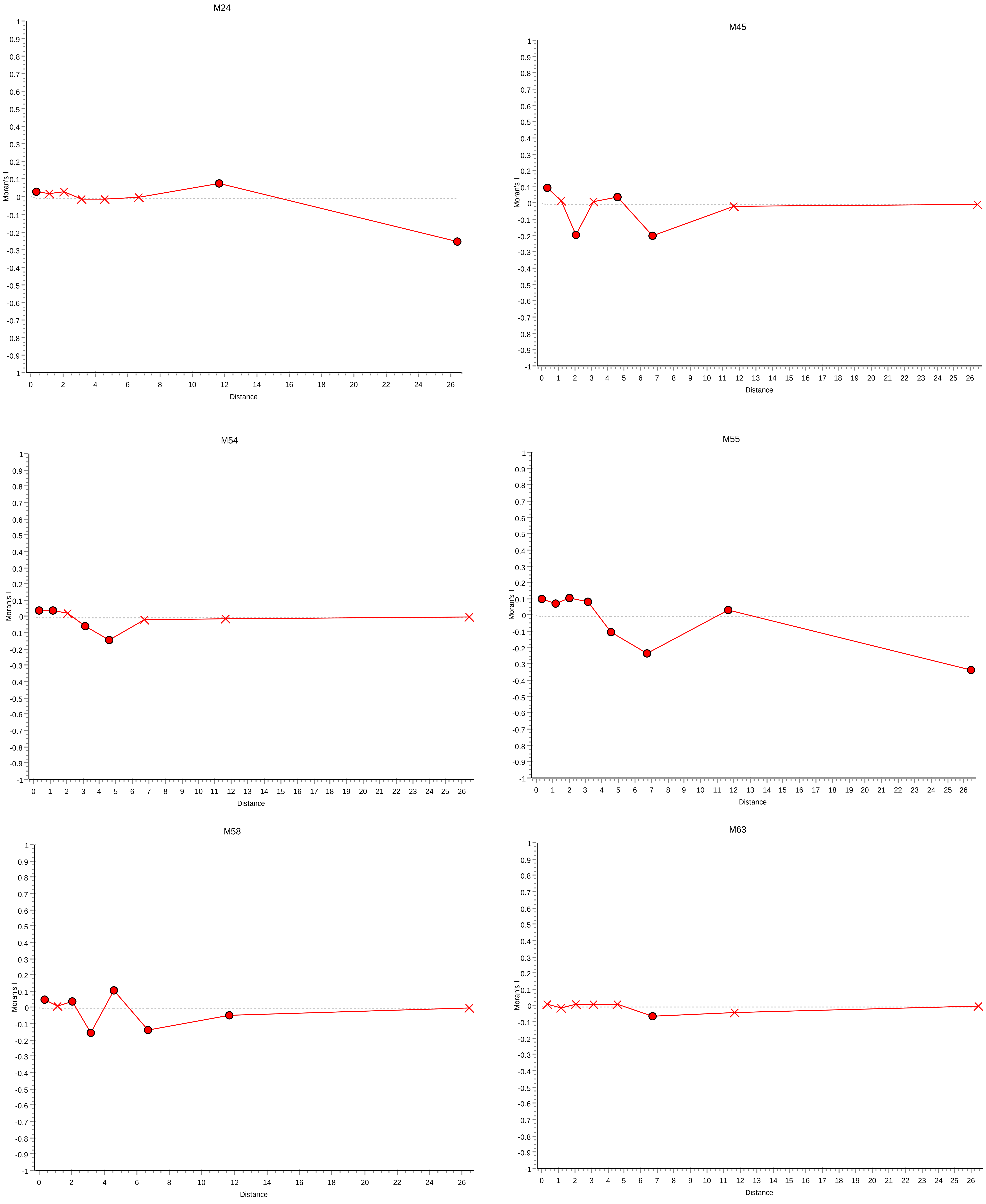

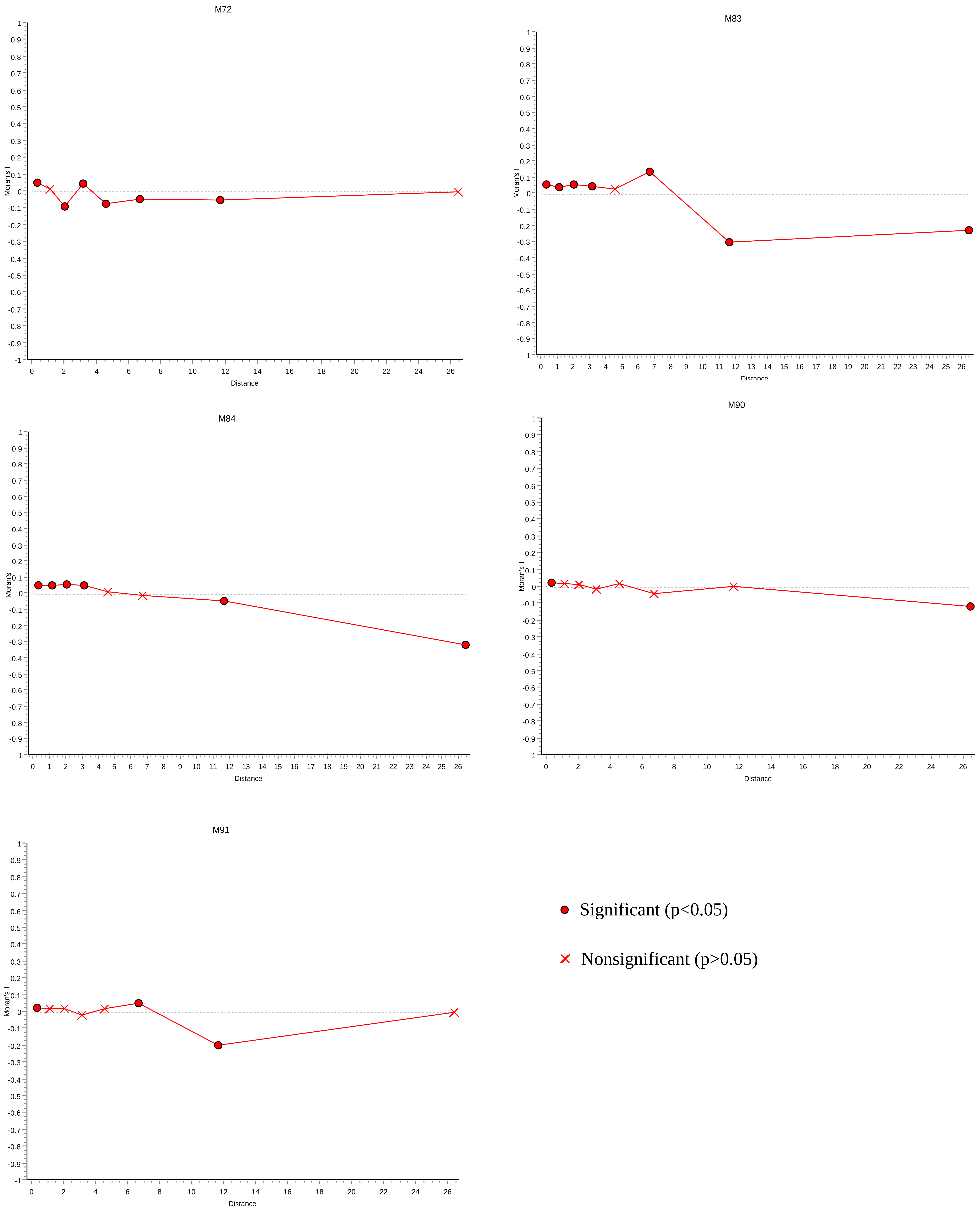
M72
Moran's I
Distance
M83
Moran's I
Distance
M84
Moran's I
Distance
M90
Moran's I
Distance
M91
Moran's I
Distance
Significant (p<0.05)
Nonsignificant (p>0.05)

## Supplementray Table S1. mtDNA variations of 845 Myanmar samples

| Code | Sample nam | Haplogroup | HVS-I (16000+) | HVS-II | Readable Region | 9bp | 4831*HhaI* | 5176*AluI* | 9820*HinfI* | 12406*HpaI* | 13262*AluI* | 14465*AccI* | Coding-Region Polymorphisms | Location | Nation |
|---|---|---|---|---|---|---|---|---|---|---|---|---|---|---|---|
| 1 | Burman025 | A | 223 274 290 319 362 527 | 73 152 235 263 315+C 522-523d | 16008-16569/1-574 | | | | | | | | | Magway | Burmans |
| 1 | Burman026 | F1a | 129 172 304 365 519 | 73 249d 263 309+C 315+C 522-523d | 16007-16569/1-573 | | | | | - | | | | Magway | Burmans |
| 1 | Burman027 | F1c | 111 129 304 519 | 73 152 234 249d 263 315+C 522-523d | 16009-16569/1-574 | | | | | - | | | | Magway | Burmans |
| 1 | Burman028 | F1a | 129 169 172 304 519 | 73 249d 263 309+CC 315+C | 16033-16569/1-319 | | | | | - | | | | Magway | Burmans |
| 1 | Burman029 | F1c | 111 129 304 519 | 73 152 234 249d 263 315+C 522-523d | 16009-16569/1-574 | | | | | - | | | | Magway | Burmans |
| 1 | Burman030 | M9a1b1 | 086 158 223 234 362 519 | 73 150 152 153 263 315+C 489 | 16007-16569/1-504 | | | | - | | | | 4238-4571=4491 | Magway | Burmans |
| 1 | Burman031 | F1c | 111 129 304 519 | 73 152 234 249d 263 309+CC 315+C 522-523d | 16000-16569/1-316 | | | | | - | | | | Magway | Burmans |
| 1 | Burman032 | A | 223 290 319 362 | 73 151 152 200 235 263 315+C 522-523d | 16014-16569/1-574 | | | | | | | | | Magway | Burmans |
| 1 | Burman033 | M7b | 086 129 189 216 223 297 | 73 150 199 263 309+C 315+C 332 489 | 16009-16569/1-574 | | | | + | | | | | Sagaing | Burmans |
| 1 | Burman034 | B5a | 092 140 182C 183C 189 266A 519 | 73 210 263 315+C 522-523d | 16012-16569/1-574 | + | | | | | | | | Sagaing | Burmans |
| 1 | Burman035 | F1a | 129 172 304 311 519 | 73 249d 263 309+CC 315+C | 16006-16569/1-319 | | | | | - | | | | Sagaing | Burmans |
| 1 | Burman036 | M49 | 153 213 223 234 257 294 519 527 | 73 263 309+C 315+C 489 | 16012-16569/1-574 | | | | | | | | 3682-4420=3780 | Sagaing | Burmans |
| 1 | Burman037 | U2b | 051 092 168 | 47 51 57 57+G 73 146 263 292 309+C 315+C 522-523d | 16012-16569/1-575 | | | | | | | | 12064-12355=12106 12308 | Sagaing | Burmans |
| 1 | Burman038 | B5a | 092 140 182C 183C 189 266A 519 | 73 210 263 315+C 522-523d | 16008-16569/1-574 | + | | | | | | | | Sagaing | Burmans |
| 1 | Burman039 | D4a | 201 223 319 362 | 73 152 263 309+C 315+C | 16017-16569/1-487 | | - | - | | | | | | Sagaing | Burmans |
| 1 | Burman040 | D4a | 201 223 319 362 | 73 152 263 309+C 315+C 489 | 16016-16569/1-575 | | - | - | | | | | | Sagaing | Burmans |
| 1 | Burman041 | C | 189 223 298 327 357 519 | 73 249d 263 309+CC 315+C | 16012-16569/1-376 | | | | | | + | | | Sagaing | Burmans |
| 1 | Burman042 | F1a1a | 108 129 162 172 304 519 | 73 249d 263 315+C 522-523d | 16008-16569/1-574 | | | | | - | | | | Sagaing | Burmans |
| 1 | Burman043 | B5a | 092 140 182C 183C 189 266A 519 | 73 210 263 315+C 522-523d | 16007-16569/1-574 | + | | | | | | | | Sagaing | Burmans |
| 1 | Burman044 | M7b | 129 192 223 297 | 73 150 199 263 315+C 489 | 16007-16569/1-574 | | | | + | | | | | Sagaing | Burmans |
| 1 | Burman045 | F1a | 129 172 295 304 519 | 73 200 249d 263 315+C | 16007-16569/1-316 | | | | | - | | | | Sagaing | Burmans |
| 1 | Burman046 | B5a | 092 140 182C 183C 189 266A 519 | 73 210 263 315+C | 16006-16569/1-426 | + | | | | | | | | Sagaing | Burmans |
| 1 | Burman048 | U2b | 051 092 168 | 47 51 57 57+G 73 146 263 292 309+C 315+C 522-523d | 16008-16569/1-575 | | | | | | | | 1502-2135=1811 | Sagaing | Burmans |
| 1 | Burman050 | F1a | 129 172 295 304 519 | 73 200 249d 263 315+C 522-523d | 16013-16569/1-575 | | | | | - | | | | Sagaing | Burmans |
| 1 | Burman051 | U2b | 051 092 168 | 47 51 57 57+G 73 146 263 292 309+C 315+C | 16007-16569/1-316 | | | | | | | | 12056-12244=12106 | Sagaing | Burmans |
| 1 | Burman052* | M58 | 129 183 218 223 293C 311 519 | 73 146 263 279 309+C 315+C | 16007-16569/1-316 | | | | | | | | 4520-5220=4769; 15016-15472=15043 15301 15326 | Sagaing | Burmans |
| 1 | Burman053 | F1a | 129 172 304 311 519 | 73 249d 263 309+C 315+C 522-523d | 16010-16569/1-575 | | | | | - | | | | Sagaing | Burmans |
| 1 | Burman054 | U2b | 051 092 168 | 47 51 57 57+G 73 146 263 292 309+C 315+C | 16013-16569/1-316 | | | | | | | | 12054-12310=12106 12308 | Sagaing | Burmans |
| 1 | Burman055 | D4a | 201 223 319 362 | 73 152 263 309+C 315+C 489 | 16007-16569/1-575 | | - | - | | | | | | Sagaing | Burmans |
| 1 | Burman056 | B5a | 092 140 182C 183C 189 266A 519 | 73 210 263 315+C 522-523d | 16007-16569/1-575 | + | | | | | | | | Sagaing | Burmans |
| 1 | Burman058 | B5a | 092 140 182C 183C 189 266A 519 | 73 210 263 315+C 522-523d | 16007-16569/1-574 | + | | | | | | | | Sagaing | Burmans |
| 1 | Burman059 | D4a | 201 223 319 362 | 73 152 263 309+C 315+C 489 | 16012-16569/1-575 | | | | | | | | | Sagaing | Burmans |
| 2 | Burman061 | D | 093 223 362 526 | 73 263 309+C 315+C 489 | 16007-16569/1-543 | | - | - | | | | | | Sagaing | Burmans |
| 2 | Burman062 | A | 179 223 290 311 319 362 | 73 152 235 263 315+C | 16025-16569/1-575 | | | | | | | | | Sagaing | Burmans |
| 2 | Burman063 | F1 | 093 183C 189 304 519 | 73 146 249d 263 309+C 315+C | 16008-16569/1-317 | | | | | - | | | | Sagaing | Burmans |
| 2 | Burman064 | B* | 051 183C 189 354 519 527 | 73 263 315+C 356+C | 16010-16569/1-575 | | | | | | | | 11727-12219=11914 | Sagaing | Burmans |
| 2 | Burman065 | G | 223 304 362 | 73 263 309+C 315+C 489 | 16007-16569/1-575 | | + | + | | | | | | Sagaing | Burmans |
| 2 | Burman066 | M76 | 124 183C 189 293C 356 362 519 | 73 263 310 | 16014-16569/1-309 | | + | - | | | | | 7924-8360=7961 8260; 4528-5446=4655 4742 4769 | Sagaing | Burmans |
| 2 | Burman068 | N8 | 223 263 274 311 318C 343 357 519 | 73 152 263 309+C 315+C | 16011-16569/1-575 | | | | | | | | 7906-8443=7961 8188 | Sagaing | Chin |
| 2 | Burman069* | M3c | 124 126 284 311 519 | 73 263 315+C 482 489 | 16007-16569/1-575 | | | | | | | | | Sagaing | Chin |
| 2 | Burman070 | G | 223 304 362 | 73 263 309+C 315+C 489 | 16001-16569/1-545 | | + | + | | | | | | Sagaing | Chin |
| 2 | Burman071* | N21 | 182 223 519 | 73 150 195 263 315+C 337d 522-523d | 16008-16569/1-574 | | | | | | | | 13071-13514=13437 | Sagaing | Burmans |
| 2 | Burman072 | R9d | 223 304 362 | 73 263 309+C 315+C 489 | 16031-16569/1-510 | | | | | | | | 12591-12857=12705 | Sagaing | Burmans |
| 2 | Burman073 | F1 | 183C 189 304 519 | 73 249d 263 309+C 315+C | 16021-16569/1-379 | | | | | - | | | | Sagaing | Burmans |
| 2 | Burman074 | R9b | 192 304 311 390 519 | 73 183 204 263 309+C 315+C | 16021-16569/1-454 | | | | | | | | 12644-12837=12714 | Sagaing | Burmans |
| 2 | Burman075 | R9b | 192 304 309 390 519 | 73 183 204 263 309+C 315+C | 16007-16569/1-316 | | | | | | | | 12691-12736=12714 | Sagaing | Burmans |
| 2 | Burman076 | D4 | 223 256 311 362 519 | 73 146 200 263 315+C 489 | 16022-16569/1-575 | | - | - | | | | | | Sagaing | Burmans |
| 2 | Burman077 | M7a | 209 223 243 304 519 | 73 152 199 263 315+C 489 513 | 16007-16569/1-575 | | | | | | | | 6364-6908=rCRS | Sagaing | Burmans |
| 2 | Burman078 | A | 189 223 290 319 | 64 73 151 152 235 263 315+C | 16023-16193/16199-316 | | | | | | | | | Sagaing | Burmans |
| 2 | Burman079 | M24 | 075 223 311 519 | 73 146 152 195 263 309+C 489 | 16021-16569/1-515 | | | | | | | | 15012-15800=15043 15301 15326 15601 15607 | Sagaing | Burmans |
| 2 | Burman081 | F1a | 129 172 189 304 519 | 73 152 249d 263 315+C 522-523d | 16009-16569/1-575 | | | | | - | | | | Sagaing | Burmans |
| 2 | Burman082 | U2b | 051 168 224 239 357 | 73 146 195 263 309+CC 315+C | 16021-16569/1-575 | | | | | | | | 12059-12609=12106 12308 12372 | Sagaing | Burmans |
| 2 | Burman083 | A | 189 223 290 319 | 64 73 151 152 235 263 315+C 522-523d | 16008-16569/1-575 | | | | | | | | | Sagaing | Burmans |
| 2 | Burman084 | R9b | 192 304 309 390 519 | 73 183 204 263 309+C 315+C | 16021-16569/1-363 | | | | | | | | 12589-13057=12714 | Sagaing | Burmans |
| 2 | Burman085 | M90 | 223 381 390 | 61A 62 73 146 152 263 309+C 315+C 489 | 16012-16569/1-575 | | | | | | | | 5813-6362=5910 6023 6253 | Sagaing | Burmans |
| 2 | Burman086 | M90 | 223 381 390 | 61A 62 73 146 152 263 309+C 315+C 489 | 16009-16569/1-575 | | | | | | | | 5811-6363=5910 6023 6253 | Sagaing | Burmans |
| 2 | Burman087 | D4b2 | 223 519 | 73 263 309+C 315+C 489 522-523d | 16007-16569/1-575 | | | | | | | | | Sagaing | Burmans |
| 2 | Burman088* | M90 | 223 381 390 | 61A 62 73 146 152 263 309+C 315+C 489 | 16007-16569/1-575 | | | | | | | | | Sagaing | Burmans |
| 2 | Burman089 | R9b | 192 304 309 390 519 | 73 183 204 263 309+C 315+C 522-523d | 16013-16569/1-575 | | | | | | | | 12596-13178=12714 | Sagaing | Burmans |
| 2 | Burman090 | F1 | 183C 189 304 519 | 73 249d 263 309+C 315+C | 16007-16569/1-316 | | | | | - | | | | Sagaing | Burmans |
| 2 | Burman091 | M4a | 145 192 223 300 316 519 | 73 146 263 309+CC 315+C 489 | 16008-16569/1-575 | | | | | | | | 11729-12146=12007 | Sagaing | Burmans |
| 2 | Burman092* | M84 | 223 258C 272 519 | 73 185H 263 309+C 315+C 489 | 16014-16569/1-575 | | | | | | | | 14079-14596=14110 | Sagaing | Burmans |
| 2 | Burman093 | B4 | 183C 189 217 234 519 | 73 262 263 315+C | 16007-16569/1-575 | + | | | | | | | | Sagaing | Burmans |
| 2 | Burman094 | M84 | 223 258C 272 519 | 73 185 263 309+C 315+C | 16012-16569/1-316 | | | | | | | | 14076-14604=14110 | Sagaing | Burmans |

| Code | Sample nam | Haplogroup | HVS-I (16000+) | HVS-II | Readable Region | 9bp | 4831*Hha*I | 5176*Alu*I | 9820*Hinf*I | 12406*Hpa*I | 13262*Alu*I | 14465*Acc*I | Coding-Region Polymorphisms | Location | Nation |
|---|---|---|---|---|---|---|---|---|---|---|---|---|---|---|---|
| 2 | Burman095 | F1c | 111 129 304 519 | 73 152 234 249d 263 315+C 522-523d | 16012-16569/1-575 | | | | | - | | | | Sagaing | Burmans |
| 2 | Burman096 | F1a | 129 172 304 519 | 73 249d 263 315+C 522-523d | 16002-16569/1-575 | | | | | - | | | | Sagaing | Burmans |
| 2 | Burman097 | D5a2 | 092 172 182C 183C 189 223 266 362 | 73 150 263 309+CC 315+C | 16018-16569/1-320 | | - | - | | | | | | Sagaing | Burmans |
| 2 | Burman099 | D5a2 | 092 164 172 182C 183C 189 223 266 362 519 | 73 150 263 309+C 315+C | 16008-16569/1-316 | | - | - | | | | | | Sagaing | Burmans |
| 2 | Burman100 | F1a | 129 172 304 519 | 73 249d 263 309+CC 315+C 522-523d | 16013-16569/1-575 | | - | - | | | | | 6364-6799=6392 6515 | Sagaing | Burmans |
| 2 | Burman101 | G | 037 129 223 261 362 519 | 73 152 235 263 309+C 315+C 489 | 16010-16569/1-575 | | + | + | | | | | | Sagaing | Burmans |
| 2 | Burman102 | G | 037 129 223 261 362 519 | 73 152 235 263 309+C 315+C 489 | 16009-16569/1-505 | | + | + | | | | | | Sagaing | Burmans |
| 2 | Burman103 | G | 037 129 223 261 362 519 | 73 152 235 263 309+C 315+C 489 | 16013-16569/1-575 | | + | + | | | | | | Sagaing | Burmans |
| 2 | Burman104 | G | 037 129 223 261 362 519 | 73 152 235 263 309+C 315+C 489 | 16012-16569/1-575 | | + | + | | | | | 4521-5181=4769 | Sagaing | Burmans |
| 2 | Burman105 | D4g2a | 223 274 362 519 | 73 263 298 309+CC 315+C 489 | 16007-16569/1-575 | | | | | | | | | Sagaing | Burmans |
| 2 | Burman107 | F1a | 129 172 304 519 | 73 249d 263 309+CC 315+C 522-523d | 16014-16569/1-571 | | | | | - | | | | Sagaing | Burmans |
| 2 | Burman108 | M5 | 129 223 311 | 73 263 315+C 489 | 16005-16569/1-575 | | | | | | | | 4519-5223=4769; 15011-15472=15043 15301 15326 | Sagaing | Burmans |
| 2 | Burman109 | F | 183C 189 304 519 | 73 249d 263 309+C 315+C 464 522-523d | 16013-16569/1-575 | | | | | | | | 6364-6890=6620; 12606-12802=rCRS | Sagaing | Burmans |
| 2 | Burman110 | R9b | 192 304 309 390 519 | 73 183 204 263 309+C 315+C 522-523d | 16012-16569/1-575 | | | | | | | | 6364-6742=6620 | Sagaing | Burmans |
| 2 | Burman111 | R9b | 192 304 309 390 519 | 73 183 204 263 309+C 315+C | 16001-16569/1-517 | | | | | | | | 12598-12937=12882 | Sagaing | Burmans |
| 2 | Burman112 | N21 | 182 193 223 260 519 | 73 150 195 263 309+C 315+CC 337d | 16013-16569/1-575 | | | | | | | | 13106-13609=13437 | Sagaing | Burmans |
| 2 | Burman113 | R9b | 192 304 309 390 519 | 73 183 204 263 309+C 315+C 522-523d | 16013-16569/1-575 | | | | | | | | 14617-15070=14766 14953 | Sagaing | Burmans |
| 2 | Burman114 | M12a | 172 183C 189 209 223 234 290 291 519 | 73 125 127 128 146 195 263 309+C 315+C 489 | 16013-16569/1-552 | | | | | | | | 13093-13926=rCRS; 6365-6906=6446 | Sagaing | Burmans |
| 2 | Burman115* | N21 | 182 193 223 260 519 | 73 150 195 263 309+C 315+C 337d | 16013-16569/1-317 | | | | | | | | | Sagaing | Burmans |
| 2 | Burman116 | M31a2 | 093 126 145 223 | 73 195 263 315+C 489 | 16015-16569/1-575 | | | | | | - | | 15014-15827=15043 15258 15301 15326 15440 15530 | Sagaing | Burmans |
| 2 | Burman117 | F1a | 129 172  304 519 | 73 249d 263 315+C 522-523d | 16019-16569/1-575 | | | | | - | | | | Sagaing | Burmans |
| 3 | Burman176 | M90 | 223 381 390 | 61A 62 73 146 152 263 309+C 315+C | 16032-16569/1-455 | | | | | | | | | Sagaing | Burmans |
| 3 | Burman177 | R9b | 124 148 304 309 327 390 519 | 73 204 263 309+C 315+C 523+CA | 16013-16569/1-575 | | | | | | | | 12599-12910=12714 | Sagaing | Burmans |
| 3 | Burman178 | R9b | 124 148 304 309 327 390 519 | 73 204 263 309+C 315+C 523+CA | 16013-16569/1-575 | | | | | | | | 12598-13039=12714 | Sagaing | Burmans |
| 3 | Burman179 | M9a1b1 | 158 223 234 311 362 519 | 73 150 152 153 263 315+C 489 | 16012-16569/1-575 | | | | | | | | 4237-4787=4491 4769 | Sagaing | Burmans |
| 3 | Burman180 | R9b | 124 148 304 309 327 390 519 | 73 204 263 309+C 315+C 523+CA | 16013-16569/1-575 | | | | | | | | 12598-12967=12714 | Sagaing | Burmans |
| 3 | Burman181 | M46 | 223 278 327 343T 519 | 73 146 152 189 196+T 309+C 315+C 489 522-523d | 16013-16569/1-575 | | | | | | | | 5811-6360=6032 6253 | Sagaing | Burmans |
| 3 | Burman182 | U2b | 051 092 209 239 311 352 353 | 73 146 152 234 263 315+C | 16012-16569/1-575 | | | | | | | | 12059-12507=12106 12308 12372 | Sagaing | Burmans |
| 3 | Burman183 | M50a | 042 093 223 248 263 311 519 | 73 152 263 315+C 356+C 489 522-523d | 16044-16569/1-575 | | | | | | | | | Sagaing | Burmans |
| 3 | Burman184 | R9b | 124 148 304 309 327 390 519 | 73 204 263 309+C 315+C 523+CA | 16013-16569/1-575 | | | | | | | | 12605-12930=12714 | Sagaing | Burmans |
| 3 | Burman185 | D4g2a | 223 274 362 | 73 150 263 298 309+C 315+C 489 | 16013-16569/1-575 | | | | | | | | 4528-5000=4769 4883 | Sagaing | Burmans |
| 3 | Burman186 | R9b | 124 148 304 309 327 390 519 | 73 204 263 | 16018-16569/1-272 | | | | | | | | 12591-13138=12714 | Sagaing | Burmans |
| 3 | Burman187 | R9b | 124 148 304 309 327 390 519 | 73 204 263 309+C 315+C 523+CA | 16013-16569/1-575 | | | | | | | | 12594-12965=12714 | Sagaing | Burmans |
| 3 | Burman188 | D4b2 | 223 519 | 73 263 309+C 315+C 489 522-523d | 16013-16569/1-575 | | | | | | | | | Sagaing | Burmans |
| 3 | Burman189 | M90 | 223 381 390 | 61A 62 73 146 152 263 309+C 315+C 489 | 16013-16569/1-575 | | | | | | | | 5843-6290=5910 6023 6253 | Sagaing | Burmans |
| 3 | Burman190 | M90 | 223 381 390 | 61A 62 73 146 152 263 309+C 315+C 489 | 16007-16569/1-575 | | | | | | | | 11023-11821=11719; 14077-14694 =rCRS | Sagaing | Burmans |
| 3 | Burman191 | G2a | 223 278 362 | 73 260 263 309+C 315+C 489 | 16012-16569/1-575 | | | | | | | | 4520-5223=4769 5108 | Sagaing | Burmans |
| 3 | Burman192 | R31 | 223 289 304 526 | 73 152 183 184 185 204 207 263 315+CC | 16007-16569/1-460 | | | | | | | | 11023-11825=11719; 14098-14702=rCRS | Sagaing | Burmans |
| 3 | Burman193 | B5a | 129 140 182C 183C 189 261 266A 519 | 73 152 210 263 315+C | 16007-16569/1-316 | | | | | | | | 11033-11755=11465 11719; 14075-14449=rCRS | Sagaing | Burmans |
| 3 | Burman194 | M90 | 223 381 390 | 61A 62 73 146 152 263 309+C 315+C | 16007-16569/1-316 | | | | | | | | 11025-11779=11719; 14072-14338 =rCRS | Sagaing | Burmans |
| 3 | Burman195 | M90 | 223 381 390 | 61A 62 73 146 152 263 309+C 315+C | 16016-16569/1-316 | | | | | | | | | Sagaing | Burmans |
| 3 | Burman196 | M84 | 223 258d 272 519 | 73 185 263 309+C 315+C | 16013-16569/1-316 | | | | | | | | 14077-14705=14110 | Sagaing | Chin |
| 3 | Burman197 | A | 223 290 319 362 519 | 73 151 152 200 235 263 522-523d | 16011-16569/1-575 | | | | | | | | 11033-11719=rCRS; 14090-14702=rCRS | Sagaing | Burmans |
| 3 | Burman198* | M45 | 519 | 73 146 152 153 234 263 309+C 315+C 489 | 16227-16569/1-573 | | | | | | | | 1803-2369=rCRS; 2432-4962=2706 3504 3669 3808 4734 4769; 5309-5928=rCRS; 11023-11797=11719; 14075-14701=rCRS | Sagaing | Burmans |
| 3 | Burman199 | F1 | 189 304 311 400 519 | 73 146 249d 263 315+C 522-523d | 16012-16569/1-575 | | | | | | | | 6364-6653=6392 | Sagaing | Burmans |
| 3 | Burman200 | R22a | 169 224 249 265C 288 291 304 519 | 73 152 199 263 309+C 315+C 329 | 16013/16569/1-574 | | | | | | | | 12600-12929=12609; 13072-13671=13359 | Sagaing | Burmans |
| 3 | Burman201 | F1 | 189 304 311 400 519 | 73 146 249d 263 315+C 522-523d | 16016-16569/1-575 | | | | | | | | 6357-6758=6392 | Sagaing | Burmans |
| 3 | Burman202 | C | 223 298 311 325 327 357 | 73 249d 263 310 | 16013-16569/1-315 | | | | | | | | 13073-13576=13263 | Sagaing | Burmans |
| 3 | Burman203 | F1b | 183C 189 232A 249 302 304 519 | 73 152 249d 263 309+C 315+C 522-523d | 16013-16569/1-575 | | | | | | | | 6364-6780=6392 | Sagaing | Burmans |
| 3 | Burman204 | D5a | 093 172 182C 183C 189 223 266 362 | 73 150 263 309+C 315+C | 16014-16569/1-319 | | | | | | | | 11028-11797=11719; 14075-14694=rCRS | Sagaing | Burmans |
| 3 | Burman205 | M90 | 223 381 390 | 61A 62 73 146 152 263 309+C 315+C 489 | 16013-16569/1-575 | | | | | | | | 5831-6270=5910 6023 6253 | Sagaing | Burmans |
| 3 | Burman206 | D5a2 | 092 172 182C 183C 189 223 266 362 | 73 150 263 309+C 315+C | 16012-16569/1-317 | | | | | | | | | Sagaing | Burmans |
| 3 | Burman207 | D5a2 | 092 172 182C 183C 189 223 266 362 | 73 150 263 309+C 315+C 489  522-523d | 16012-16569/1-575 | | | | | | | | | Sagaing | Burmans |
| 3 | Burman208 | D5a | 092 182C 183C 189 223 266 362 | 73 150 263 309+CC 315+C | 16007-16569/1-319 | | | | | | | | 11033-11821=11719; 14074-14703=rCRS | Sagaing | Burmans |
| 3 | Burman209 | M90 | 125 223 381 390 | 61A 62 73 146 152 263 309+C 315+C | 16007-16569/1-316 | | | | | | | | 11033-11798=11440 11719; 14071-14697=rCRS | Sagaing | Burmans |
| 3 | Burman210 | R22a | 169 224 249 265C 288 291 304 519 | 73 152 199 263 309+C 315+C | 16013-16569/1-317 | | | | | | | | 12589-12966=12609; 13085-13653=13539 | Sagaing | Burmans |
| 3 | Burman211 | M90 | 223 381 390 | 61A 62 73 146 152 263 309+C 315+C 489 | 16012-16569/1-575 | | | | | | | | 5831-6362=5910 6023 6253 | Sagaing | Burmans |
| 3 | Burman212 | D4e | 223 291 362 | 73 94 263 315+C 489 | 16017-16569/1-476 | | | | | | | | 4521-523=4769 4883 5178A | Sagaing | Burmans |
| 3 | Burman213 | R22a | 169 249 265C 288 291 304 519 | 73 152 199 263 309+C 315+C | 16012-16569/1-316 | | | | | | | | 11022-11866=11719; 14076-14695=rCRS | Sagaing | Burmans |
| 3 | Burman214 | M71 | 129 136 140 223 271 519 | 46 73 143 146 151 235 263 315+C 489 | 16013-16569/1-575 | | | | | | | | 11022-11832=11626 11719; 14065-14693=14605 | Sagaing | Burmans |
| 3 | Burman215 | D4 | 129 223 362 | 73 152 263 309+C 315+C 489 | 16007-16569/1-575 | | | | | | | | 11033-11753=11719; 14071-14694=14668 | Sagaing | Burmans |
| 3 | Burman216 | F1a | 129 172 304 519 | 73 249d 263 315+C | 16016-16569/1-348 | | | | | | | | 6364-6763=6392 6515 | Sagaing | Burmans |
| 3 | Burman217 | D4b2 | 223 519 | 73 263 309+C 315+C 489 522-523d | 16007-16569/1-575 | | | | | | | | | Sagaing | Burmans |
| 3 | Burman218 | K | 093 224 311 362 519 | 73 263 315+C 497 523+CA | 16010-16569/1-574 | | | | | | | | | Sagaing | Burmans |

| Code | Sample nam | Haplogroup | HVS-I (16000+) | HVS-II | Readable Region | 9bp | 4831*Hha1* | 5176*Alu1* | 9820*Hinf1* | 12406*Hpa1* | 13262*Alu1* | 14465*Acc1* | Coding-Region Polymorphisms | Location | Nation |
|---|---|---|---|---|---|---|---|---|---|---|---|---|---|---|---|
| 3 | Burman219 | R22a | 169 224 249 265C 288 291 304 519 | 73 152 199 263 309+C 315+C 329 | 16017-16569/1-575 | | | | | | | | | Sagaing | Burmans |
| 3 | Burman220 | M90 | 223 381 390 | 61A 62 73 146 152 263 309+C 315+C 489 | 16018-16569/1-575 | | | | | | | | 5814-6280=5910 6023 6253 | Sagaing | Burmans |
| 3 | Burman221 | M90 | 223 381 390 | 61A 62 73 146 152 263 309+C 315+C | 16009-16569/1-316 | | | | | | | | 11033-11864=11719; 14073-14694=rCRS | Sagaing | Burmans |
| 3 | Burman222 | M90 | 223 381 390 | 61A 62 73 146 152 263 309+C 315+C 489 | 16007-16569/1-575 | | | | | | | | 5813-6059=5910 6023 | Sagaing | Burmans |
| 3 | Burman223 | R22a | 169 224 249 265C 288 291 304 519 | 73 152 199 263 309+C 315+C 329 | 16013-16569/1-575 | | | | | | | | 12597-12909=12609; 13084-13533=13359 | Sagaing | Burmans |
| 3 | Burman224 | M9a1b1 | 158 223 234 362 519 | 73 150 152 263 309+C 315+C 489 | 16012-16569/1-575 | | | | | | | | 4237-4862=4491 4769 | Sagaing | Burmans |
| 3 | Burman225 | U2b | 051 093 168 172 359 | 73 146 263 315+C | 16012-16569/1-316 | | | | | | | | 12042-12512=12106 12308 12372 | Sagaing | Burmans |
| 3 | Burman226 | D | 223 311H 362 519 | 73 263 315+C 489 522-523d | 16006-16569/1-575 | | | | | | | | | Sagaing | Burmans |
| 4 | Burman515 | M63 | 223 399 488 497 519 | 73 214 263 309+C 315+C 489 | 16019-16569/1-574 | | | | | | | | 11756-12313=12007 12239 | Magway | Burmans |
| 4 | Burman516 | M63 | 223 399 488 497 519 | 73 214 263 309+C 315+C 489 | 16043-16569/1-574 | | | | | | | | 11756-12268=12007 12239 | Magway | Burmans |
| 4 | Burman517 | G2a1 | 136 223 227 278 362 | 73 263 309+C 315+C 489 523+CA | 16042-16569/1-574 | | + | | | | | | | Magway | Burmans |
| 4 | Burman518 | B5a | 111 140 183C 189 266A 519 | 73 210 263 315+C 522-523d | 16012-16569/1-574 | + | | | | | | | | Magway | Burmans |
| 4 | Burman519 | C | 093 129 223 298 327 519 | 73 249d 263 315+C 489 | 16018-16569/1-574 | | | | | | + | | | Magway | Burmans |
| 4 | Burman520 | D | 167 223 362 | 73 94 263 309+C 315+C 489 523+CA | 16020-16569/1-574 | | - | - | | | | | | Magway | Burmans |
| 4 | Burman521 | M91 | 129 223 311 327A | 64 73 93 200 263 309+C 315+C 485 489 | 16045-16569/1-574 | | | | | | | | 9278-9550=9509 9540 | Magway | Burmans |
| 4 | Burman522 | G | 114 223 362 | 73 263 309+C 315+C 489 | 16022-16569/1-574 | | + | | | | | | | Magway | Burmans |
| 4 | Burman523 | N21 | 182 193 223 260 310 519 | 73 150 195 214 263 315+C 337d | 16027-16569/1-574 | | | | | | | | 13148-13660=13437 | Magway | Burmans |
| 4 | Burman524 | F3a | 183C 189 221 298 355 362 519 | 73 249d 263 309+C 315+C | 16017-16569/1-319 | | | | | + | | | | Magway | Burmans |
| 4 | Burman525 | G | 114 223 362 | 73 263 309+C 315+C 489 | 16019-16569/1-574 | | + | | | | | | | Magway | Burmans |
| 4 | Burman526 | C | 093 129 223 298 327 519 | 73 249d 263 315+C 489 | 16007-16569/1-574 | | | | | | + | | | Magway | Burmans |
| 4 | Burman527 | N21 | 182 193 223 260 310 519 | 73 150 195 214 263 315+C 337d | 16008-16569/1-574 | | | | | | | | 13084-13449=13437 | Magway | Burmans |
| 4 | Burman528 | M91 | 129 223 311 327A | 64 73 93 200 263 309+C 315+C 485 489 | 16016-16569/1-574 | | | | | | | | 9224-9700=9509 9540 9554 | Magway | Burmans |
| 4 | Burman529 | N21 | 182 193 223 260 310 519 | 73 150 195 214 263 315+C 337d | 16031-16569/1-574 | | | | | | | | 13105-13570=13437 | Magway | Burmans |
| 4 | Burman530 | B5a | 140 182C 183C 189 261 266A 304 519 | 73 152 210 263 315+C 522-523d | 16040-16569/1-574 | + | | | | | | | | Magway | Burmans |
| 4 | Burman531* | M91 | 129 223 311 327A | 64 73 93 200 263 309+C 315+C 485 489 | 16012-16569/1-574 | | | | | | | | | Magway | Burmans |
| 4 | Burman532 | N21 | 182 193 223 260 310 519 | 73 150 195 214 263 315+C 337d | 16024-16569/1-574 | | | | | | | | 13085-13460=13437 | Magway | Burmans |
| 4 | Burman533 | G | 114 223 362 | 73 263 309+C 315+C 489 | 16032-16569/1-574 | | + | | | | | | | Magway | Burmans |
| 4 | Burman534* | M63 | 223 399 488 497 519 | 73 214 263 309+C 315+C 489 | 16043-16569/1-574 | | | | | | | | | Magway | Burmans |
| 4 | Burman535 | G | 114 223 362 | 73 263 309+C 315+C | 16061-16569/1-480 | | | | | | | | 4519-5226=4769 4833 4853 5108 | Magway | Burmans |
| 4 | Burman536 | F1c | 111 129 304 519 | 73 152 234 249d 263 315+C 522-523d | 16012-16569/1-574 | | | | | - | | | | Magway | Burmans |
| 4 | Burman537 | U2a | 051 140 154 206C 230 261 311 519 | 73 152 263 309+C 315+C | 16046-16569/1-316 | | | | | | | | 12058-12465=12308 12372 | Magway | Burmans |
| 4 | Burman538 | M12 | 129 172 223 290 519 | 73 152 198 263 309+C 315+C | 16001-16569/1-316 | | | | | | | | 14600-15378=14727 14766 14783 15010 15043 15301 15326 | Magway | Burmans |
| 4 | Burman539 | G | 114 223 362 | 73 263 309+C 315+C 489 | 16040-16569/1-574 | | + | | | | | | | Magway | Burmans |
| 4 | Burman540 | D5a2 | 092 164 182C 183C 189 223 266 311 362 | 73 150 263 315+C 489 522-523d | 16043-16569/1-574 | | - | - | | | | | | Magway | Burmans |
| 4 | Burman541 | C | 093 223 298 311 327 519 | 73 249d 263 315+C 489 | 16013-16569/1-574 | | | | | | + | | | Magway | Burmans |
| 4 | Burman542 | G2a1 | 136 223 227 278 362 | 73 263 309+C 315+C 489 523+CA | 16024-16569/1-574 | | + | | | | | | | Magway | Burmans |
| 4 | Burman543 | M91 | 129 223 287 311 327A | 64 73 93 146 189 200 263 309+C 315+C 485 489 | 16043-16569/1-574 | | | | | | | | 9218-9700=9509 9540 9554 | Magway | Burmans |
| 4 | Burman544 | N21 | 182 193 223 260 310 519 | 73 150 195 214 263 315+C 337d | 16038-16569/1-574 | | | | | | | | 13084-13537=13437 | Magway | Burmans |
| 4 | Burman545 | G2a1 | 136 223 227 278 362 | 73 263 309+C 315+C 489 523+CA | 16024-16569/1-574 | | + | | | | | | | Magway | Burmans |
| 4 | Burman546 | N21 | 182 193 223 260 310 519 | 73 150 195 214 263 315+C 337d | 16018-16569/1-574 | | | | | | | | 13111-13490=13437 | Magway | Burmans |
| 4 | Burman547 | U2a | 051 140 154 206C 230 261 311 519 | 73 152 263 309+C 315+C | 16008-16569/1-574 | | | | | | | | 12059-12444=12308 12372 | Magway | Burmans |
| 4 | Burman548 | F1c | 111 129 304 519 | 73 152 234 249d 263 315+C 522-523d | 16017-16569/1-574 | | | | | - | | | | Magway | Burmans |
| 4 | Burman549 | M12 | 129 172 223 290 519 | 73 152 198 263 309+C 315+C | 16012-16569/1-323 | | | | | | | | 14619-15252=14727 14766 14783 15010 15043 | Magway | Burmans |
| 4 | Burman550 | C | 093 223 298 311 327 519 | 73 249d 263 315+C 489 | 16014-16569/1-574 | | | | | | + | | | Magway | Burmans |
| 4 | Burman551 | U2a | 051 140 154 206C 230 261 311 519 | 73 152 263 309+C 315+C | 16008-16569/1-574 | | | | | | | | 12062-12384=12308 12372 | Magway | Burmans |
| 4 | Burman552 | B5a | 111 140 183C 189 266A 519 | 73 210 263 315+C 522-523d | 16016-16569/1-574 | + | | | | | | | | Magway | Burmans |
| 4 | Burman553 | U2a | 051 140 154 206C 230 261 311 519 | 73 152 263 309+C 315+C | 16018-16569/1-431 | | | | | | | | 12059-12462=12308 12372 | Magway | Burmans |
| 4 | Burman554 | G | 114 223 362 | 63 73 263 309+C 315+C | 16010-16569/1-316 | | + | + | | | | | | Magway | Burmans |
| 4 | Burman555 | M12 | 129 172 223 290 519 | 73 152 198 263 309+C 315+C | 16017-16569/1-322 | | | | | | | | 14626-14860=14727 14766 14783 | Magway | Burmans |
| 4 | Burman556 | C | 093 129 223 298 327 519 | 73 249d 263 315+C 489 | 16008-16569/1-574 | | | | | | + | | | Magway | Burmans |
| 4 | Burman557 | U2a | 051 140 154 206C 230 261 311 519 | 73 153 263 309+C 315+C | 16018-16569/1-574 | | | | | | | | 12059-12487=12308 12372 | Magway | Burmans |
| 4 | Burman558 | U2a | 051 140 154 206C 230 261 311 519 | 73 152 263 309+C 315+C | 16016-16569/1-516 | | | | | | | | 12509-12547=12308 12372 | Magway | Burmans |
| 4 | Burman559 | N21 | 182 193 223 260 519 | 73 150 195 263 315+C 337d | 16014-16569/1-574 | | | | | | | | 13156-13442=13437 | Magway | Burmans |
| 4 | Burman560 | C | 093 129 223 298 327 519 | 73 249d 263 315+C 489 | 16014-16569/1-574 | | | | | | + | | | Magway | Burmans |
| 4 | Burman561 | U2a | 051 140 154 206C 230 261 311 519 | 73 152 263 309+C 315+C | 16026-16569/1-574 | | | | | | | | 12060-12434=12308 12372 | Magway | Burmans |
| 4 | Burman562 | F1c | 111 129 304 519 | 73 152 234 249d 263 315+C 522-523d | 16017-16569/1-574 | | | | | - | | | | Magway | Burmans |
| 4 | Burman563 | C | 093 129 223 298 327 519 | 73 249d 263 315+C 489 | 16017-16569/1-574 | | | | | | + | | | Magway | Burmans |
| 4 | Burman564 | Z | 093 185 223 260 298 357 | 73 152 207 249d 263 309+C 315+C 489 523+CA | 16018-16569/1-574 | | | | | | | | 8827-9221=8860 9090 | Magway | Burmans |
| 4 | Burman565 | F1a1a | 108 129 162 172 243 293C 304 519 | 73 152 249d 263 309+C 315+C | 16015-16569/1-316 | | | | | - | | | | Magway | Burmans |
| 4 | Burman566 | M12 | 129 172 182 223 290 519 | 73 152 198 263 309+C 315+C 489 522-523d 573+CCC | 16043-16569/1-574 | | | | | | | | 14592-14884=14727 14766 14783 | Magway | Burmans |
| 4 | Burman567 | N21 | 182 193 223 260 310 519 | 73 150 195 214 263 315+C 337d | 16017-16569/1-574 | | | | | | | | 13158-13438=13437 | Magway | Burmans |
| 4 | Burman568 | M12 | 129 172 182 223 290 519 | 73 152 198 263 309+C 315+C 489 522-523d 573+CCC | 16018-16569/1-574 | | | | | | | | | Magway | Burmans |
| 4 | Burman569 | M12 | 129 172 182 223 290 519 | 73 152 198 263 309+C 315+C 489 522-523d 573+CCC | 16011-16569/1-574 | | | | | | | | 14765-15291=14766 14783 15010 15043 | Magway | Burmans |
| 4 | Burman570 | N21 | 182 193 223 260 310 519 | 73 150 195 214 263 315+C 337d | 16017-16569/1-574 | | | | | | | | 13107-13843=13437 | Magway | Burmans |
| 4 | Burman571 | U2a | 051 140 154 206C 230 261 311 519 | 73 152 263 309+C 315+C | 16007-16569/1-574 | | | | | | | | 12058-12753=12308 12372 | Magway | Burmans |

| Code | Sample nam | Haplogroup | HVS-I (16000+) | HVS-II | Readable Region | 9bp | 4831*Hha*I | 5176*Alu*I | 9820*Hinf*I | 12406*Hpa*I | 13262*Alu*I | 14465*Acc*I | Coding-Region Polymorphisms | Location | Nation |
|---|---|---|---|---|---|---|---|---|---|---|---|---|---|---|---|
| 4 | Burman572 | F1c | 111 129 304 519 | 73 152 234 249d 263 315+C 522-523d | 16017-16569/1-574 | | | | | - | | | | Magway | Burmans |
| 4 | Burman573 | G | 223 362 | 73 207 263 309+C 315+C 489 | 16020-16569/1-574 | | + | + | | | | | | Magway | Burmans |
| 4 | Burman574 | F3a | 183C 189 221 298 355 362 519 | 73 249d 263 309+C 3156+C | 16017-16569/1-319 | | | | | + | | | | Magway | Burmans |
| 4 | Burman575* | M91 | 129 223 287 311 327A | 64 73 93 146 189 200 263 309+CC 315+C 485 489 | 16009-16569/1-570 | | | | | | | | | Magway | Burmans |
| 4 | Burman576 | N21 | 182 193 223 260 310 519 | 73 150 195 214 263 315+C 337d | 16016-16569/1-574 | | | | | | | | 13117-13769=13437 | Magway | Burmans |
| 4 | Burman577 | Z | 093 185 223 260 298 357 | 73 152 207 249d 263 309+C 315+C 489 523+CA | 16012-16569/1-574 | | | | | | | | 8828-9216=8860 9090 | Magway | Burmans |
| 4 | Burman578 | F1c | 111 129 304 519 | 73 152 234 249d 263 315+C 522-523d | 16010-16569/1-574 | | | - | | - | | | | Magway | Burmans |
| 4 | Burman579 | F1c | 111 129 304 519 | 73 152 234 249d 263 315+C 522-523d | 16014-16569/1-574 | | | | | - | | | | Magway | Burmans |
| 4 | Burman580 | Z | 093 185 223 260 298 357 | 73 152 207 249d 263 309+C 315+C 489 523+CA | 16022-16569/1-574 | | | | | | | | 4536-5197=4715 4769 | Magway | Burmans |
| 4 | Burman581 | G2a1 | 136 223 227 278 362 | 73 263 309+C 315+C 489 522+CA | 16043-16569/1-574 | | + | | | | | | | Magway | Burmans |
| 4 | Burman582* | M58 | 183 189 223 266 295 519 | 73 143 153 263 309+C 315+C 489 | 16012-16569/1-574 | | | | | | | | | Magway | Burmans |
| 4 | Burman583 | N21 | 182 193 223 260 310 519 | 73 150 195 214 263 315+C 337d | 16015-16569/1-574 | | | | | | | | 13106-13650=13437 | Magway | Burmans |
| 4 | Burman584 | N21 | 182 193 223 260 310 519 | 73 150 195 214 263 315+C 337d | 16026-16569/1-574 | | | | | | | | 13084-13491=13437 | Magway | Burmans |
| 4 | Burman585* | M21c | 093 223 249 266 301 311 519 | 73 263 315+C 489 | 16018-16569/1-574 | | | | | | | | | Magway | Burmans |
| 4 | Burman586 | M10 | 223 311 519 | 73 94 195 263 309+C 315+C 489 573+CCC | 16007-16569/1-574 | | | | | | | | 15013-15677=15040 15043 15071 15218 15301 15326 | Magway | Burmans |
| 4 | Burman587 | M10 | 223 311 519 | 73 94 195 263 309+C 315+C | 16044-16569/1-410 | | | | | | | | 15011-15757=15040 15043 15071 15218 15301 15326 | Magway | Burmans |
| 4 | Burman588 | M12 | 129 172 223 290 305 | 73 152 198 263 309+C 315+C 489 522-523d | 16017-16569/1-573 | | | | | | | | 14619-15296=14727 14766 14783 15010 15043 | Magway | Burmans |
| 4 | Burman589 | F1c | 111 129 304 519 | 73 152 234 249d 263 315+C 522-523d | 16026-16569/1-574 | | | | | - | | | | Magway | Burmans |
| 4 | Burman590 | M21c | 093 223 249 266 301 311 519 | 73 263 315+C 489 | 16023-16569/1-574 | | | | | | | | 11375-11856=11482 11719 | Magway | Burmans |
| 4 | Burman591 | Z | 093 185 223 260 298 357 | 73 152 207 249d 263 309+C 315+C 489 523+CA | 16014-16569/1-574 | | | | | | | | 15630-16082=15784 15884 15928 | Magway | Burmans |
| 4 | Burman592* | M84 | 129 223 258d 272 519 | 73 185 195 263 315+C 489 | 16006-16569/1-574 | | | | | | | | 1513-2000=1719 1809 | Magway | Burmans |
| 4 | Burman593 | M91 | 129 223 287 311 327A | 64 73 93 146 189 200 263 309+C 315+C 485 489 | 16044-16569/1-319/367-574 | | | | | | | | 9256-9650=9509 9540 9554 | Magway | Burmans |
| 4 | Burman594 | M91 | 129 223 311 327A 362H | 64 73 93 200 263 309+C 315+C 485 489 | 16017-16569/1-574 | | | | | | | | 9222-9736=9509 9540 9554 | Magway | Burmans |
| 4 | Burman595 | F1c | 111 129 304 519 | 73 152 234 249d 263 315+C 522-523d | 16026-16569/1-574 | | | | | - | | | | Magway | Burmans |
| 4 | Burman596 | F1c | 111 129 304 519 | 73 152 234 249d 263 315+C 522-523d | 16043-16569/1-574 | | | | | - | | | | Magway | Burmans |
| 4 | Burman597 | C | 093 129 223 298 327 519 | 73 249d 263 315+C 489 | 16022-16569/1-574 | | | | | | + | | | Magway | Burmans |
| 4 | Burman598 | D | 167 223 362 | 73 94 263 309+C 315+C 489 523+CA | 16032-16569/1-573 | | | - | | | | | | Magway | Burmans |
| 4 | Burman599 | F1c | 111 129 304 519 | 73 152 234 249d 263 315+C 522-523d | 16019-16569/1-574 | | | | | - | | | | Magway | Burmans |
| 4 | Burman600 | N21 | 182 193 223 260 310 519 | 73 150 195 214 263 315+C 337d | 16031-16569/1-574 | | | | | | | | 13110-13737=13437 | Magway | Burmans |
| 4 | Burman601 | M63 | 223 399 488 497 519 | 73 214 263 309+C 315+C 489 | 16032-16569/1-574 | | | | | | | | 11726-12246=12007 12239 | Magway | Burmans |
| 4 | Burman602 | B5a | 111 140 183C 189 266A 519 | 73 210 263 315+C 522-523d | 16004-16569/1-574 | + | | | | | | | | Magway | Burmans |
| 4 | Burman603 | D | 167 223 362 | 73 94 263 309+C 315+C 489 523+CA | 16032-16569/1-574 | | - | - | | | | | | Magway | Burmans |
| 4 | Burman604 | G2a1 | 136 223 227 278 362 | 73 263 309+C 315+C 489 523+CA | 16010-16569/1-574 | | + | | | | | | | Magway | Burmans |
| 4 | Burman605 | D | 167 223 362 | 73 94 263 309+C 315+C 489 523+CA | 16018-16569/1-574 | | - | - | | | | | | Magway | Burmans |
| 4 | Burman606* | M90 | 086 223 381 390 | 73 150 227 263 309+C 315+C 489 | 16021-16569/1-514 | | | | | | | | | Magway | Burmans |
| 4 | Burman607 | A | 189 223 290 319 | 64 73 151 152 235 263 315+C 522-523d | 16018-16569/1-574 | | | | | | | | | Magway | Burmans |
| 4 | Burman608 | A | 189 223 290 319 | 64 73 151 152 235 263 315+C 522-523d | 16018-16569/1-574 | | | | | | | | | Magway | Burmans |
| 4 | Burman609 | D4g2a | 223 274 362 | 73 150 263 298 309+C 315+C 489 | 16001-16569/1-574 | | | | | | | | | Magway | Burmans |
| 4 | Burman610 | M9a1b1 | 158 223 234 362 519 | 73 150 152 153 263 315+C | 16016-16569/1-392 | | | | | | | | 4257-4806=4491 4769 | Magway | Burmans |
| 4 | Burman611 | B5a | 140 182C 183C 189 260 261 266A 519 | 73 152 210 263 309+CC 315+C | 16023-16569/1-316 | + | | | | | | | | Magway | Burmans |
| 4 | Burman612* | M33a | 075 136 223 519 | 8T 12 73 263 309+C 315+C 489 513 519 | 16019-16569/1-574 | | | | | | | | | Magway | Burmans |
| 4 | Burman613* | R22a | 169 249 265C 288 293 304 519 | 73 152 199 263 309+C 315+C 329 | 16022-16569/1-574 | | | | | | | | | Magway | Burmans |
| 4 | Burman614* | N21 | 189 223 519 | 73 150 195 263 309+C 315+C 337d | 16024-16569/1-478 | | | | | | | | | Magway | Burmans |
| 4 | Burman615 | M83 | 129 223 519 527 | 73 263 309+C 315+C 356+C 489 | 16010-16569/1-574 | | | | | | | | 7903-8488=8059 8143 8307 | Magway | Burmans |
| 4 | Burman616 | G1 | 184 223 290 362 519 | 73 152 263 309+C 315+C 489 | 16044-16569/1-574 | | | | | | | | 4520-5025=4769 4833; 8245-9027=8701 8860 8940 | Magway | Burmans |
| 4 | Burman617 | M50a | 042 093 223 248 263 311 519 | 73 152 263 315+C 356+C 489 522-523d | 16015-16569/1-574 | | | | | | | | 15012-15853=15043 15119 15301 15326 15663 | Magway | Burmans |
| 4 | Burman618* | M50a | 042 093 223 248 263 311 519 | 73 152 263 315+C 356+C 489 522-523d | 16014-16569/1-574 | | | | | | | | 15011-15275=15043 15119 | Magway | Burmans |
| 4 | Burman619 | D5a2 | 092 172 182C 183C 189 223 266 362 | 73 150 189 263 309+C 315+C 489 522-523d | 16019-16569/1-574 | | - | - | | | | | | Magway | Burmans |
| 4 | Burman620 | A | 223 290 319 362 519 | 73 151 152 200 235 263 315+C | 16026-16569/1-506 | | | | | | | | | Magway | Burmans |
| 4 | Burman621* | M3c | 086 126 223 519 | 66T 73 152 263 309+C 315+C 482 489 522-523d | 16018-16569/1-574 | | | | | | | | | Magway | Burmans |
| 4 | Burman622* | M83 | 129 223 519 527 | 73 263 309+C 315+C | 16023-16569/1-323 | | | | | | | | | Magway | Burmans |
| 4 | Burman623 | M90 | 223 381 390 | 61A 62 73 146 152 263 309+C 315+C 489 | 16021-16569/1-574 | | | | | | | | 5833-6365=5910 6023 6253 | Magway | Burmans |
| 4 | Burman624 | C | 051 223 298 327 | 73 195 249d 263 309+C 315+C 489 | 16004-16569/1-574 | | | | | | + | | | Magway | Burmans |
| 4 | Burman625 | M90 | 223 381 390 | 61A 62 73 146 152 263 309+C 315+C 489 | 16007-16569/1-574 | | | | | | | | 5811-6270=5910 6023 6253 | Magway | Burmans |
| 4 | Burman626 | F1a | 129 172 304 519 | 73 249d 263 315+C 522-523d | 16004-16569/1-574 | | | | | - | | | | Magway | Burmans |
| 4 | Burman627 | U2 | 051 318 | 73 146 263 309+C 315+C | 16012-16569/1-574 | | | | | | | | 12068-12276=12106 | Magway | Burmans |
| 4 | Burman628 | C | 223 298 327 354 357 519 | 73 93 249d 263 309+C 315+C | 16029-16569/1-323 | | | | | | + | | | Magway | Burmans |
| 4 | Burman629 | D5a2 | 092 172 182C 183C 189 223 266 362 | 73 150 263 309+C 315+C 489 522-523d | 16013-16569/1-574 | | - | - | | | | | | Magway | Burmans |
| 4 | Burman630 | M9a1b1 | 158 223 234 362 519 | 73 150 152 153 263 315+C 489 | 16012-16569/1-506 | | | | | | | | 4237-4697=4491 | Magway | Burmans |
| 4 | Burman631* | M46 | 223 278 327 343T 519 | 73 146 152 189 196+T 309+C 315+C 489 522-523d | 16007-16569/1-551 | | | | | | | | | Magway | Burmans |
| 4 | Burman632 | A | 189 223 290 319 | 64 73 151 152 235 263 315+C 522-523d | 16030-16569/1-574 | | | | | | | | | Magway | Burmans |
| 4 | Burman633 | N21 | 182 193 223 260 519 | 73 150 195 263 309+C 315+C 337d | 16012-16569/1-572 | | | | | | | | 13089-13499=13437 | Magway | Burmans |
| 4 | Burman634 | M90 | 086 223 381 390 | 73 150 227 263 309+C 315+C 489 | 16024-16569/1-441 | | | | | | | | 5831-6278=5910 6023 6253 | Magway | Burmans |
| 4 | Burman635 | A | 189 223 290 319 | 64 73 151 152 235 263 315+C 522-523d | 16007-16569/1-574 | | | | | | | | | Magway | Burmans |
| 4 | Burman636 | M90 | 086 223 381 390 | 73 150 227 263 309+C 315+C 489 | 16017-16569/1-574 | | | | | | | | 5821-6204=5910 6023 | Magway | Burmans |

| Code | Sample nam | Haplogroup | HVS-I (16000+) | HVS-II | Readable Region | 9bp | 4831*Hha*1 | 5176*Alu*1 | 9820*Hinf*1 | 12406*Hpa*1 | 13262*Alu*1 | 14465*Acc*1 | Coding-Region Polymorphisms | Location | Nation |
|---|---|---|---|---|---|---|---|---|---|---|---|---|---|---|---|
| 5 | Burman650 | N21 | 093 182 193 223 260 519 | 73 150 195 263 309+C 315+C 337d | 16008-16569/1-468 | | | | | | | | 13089-13672=13437 | Bago | Burmans |
| 5 | Burman651 | F1 | 172 304 519 | 73 249d 263 315+C 522-523d | 16008-16569/1-574 | | | | | | | | 6364-6816=6392 6515 | Bago | Burmans |
| 5 | Burman652 | M84 | 183d 223 224 258d 272 519 | 73 146 185 263 309+C 315+C 489 | 16043-16569/1-599 | | | | | | | | 1502-2000=1719 | Bago | Burmans |
| 5 | Burman653 | D5 | 189 223 362 519 | 73 150 309+CC 315+C 456 489 | 16043-16569/1-596 | | - | - | | | | | | Bago | Burmans |
| 5 | Burman654 | R22a | 169 224 249 265C 288 291 304 519 | 73 152 199 263 315+C 329 | 16042-16569/1-596 | | | | | | | | 12600-12796=12609 | Bago | Burmans |
| 5 | Burman655 | M13b | 145 168 188 223 257 311 519 | 73 152 263 315+C 489 513 | 16018-16569/1-596 | | | | | | | | 10187-10744=10373 10398 10400 10411 | Bago | Burmans |
| 5 | Burman656 | A | 223 256 290 319 362 | 73 151 152 200 235 263 315+C 522-523d | 16014-16569/1-596 | | | | | | | | | Bago | Burmans |
| 5 | Burman657 | M20 | 086 129 209 223 272 519 | 73 152 225 249d 263 315+C 316 489 522-523d | 16018-16569/1-590 | | | | | | | | 12053-12615=12354; 14076-14704=14110 | Bago | Burmans |
| 5 | Burman658 | R22a | 169 224 249 265C 288 291 304 519 | 73 152 199 263 315+C 329 | 16045-16569/1-562 | | | | | | | | 12596-12958=12609; 13085-13599=13359 | Bago | Burmans |
| 5 | Burman659 | F1a | 129 169 172 304 519 | 73 249d 263 309+CC 315+C 522-523d | 16018-16569/1-497 | | | | | - | | | | Bago | Burmans |
| 5 | Burman660 | M10 | 223 254 311 519 | 73 94 195 263 309+C 315+C 489 | 16013-16569/1-572 | | | | | | | | 15012-15720=15040 15043 15071 15218 15301 15326 | Bago | Burmans |
| 5 | Burman661 | F1c | 111 129 304 519 | 73 152 234 249d 263 315+C 522-523d | 16012-16569/1-585 | | | | | - | | | | Bago | Burmans |
| 5 | Burman662 | A | 223 256 290 319 362 | 73 151 152 200 235 263 315+C 522-523d | 16012-16569/1-572 | | | | | | | | | Bago | Burmans |
| 5 | Burman663 | M5a2a1 | 129 223 264 265C 311 519 | 73 263 309+C 315+C 489 | 16077-16569/1-559 | | | | | | | | 14077-14597=14323 | Bago | Burmans |
| 5 | Burman664 | F1c | 111 129 189 304 519 | 73 152 185 234 249d 263 315+C 522-523d | 16024-16569/1-599 | | | | | - | | | | Bago | Burmans |
| 5 | Burman665 | F1a | 129 172 304 519 | 73 249d 263 315+C 522-523d | 16068-16569/1-565 | | | | | - | | | | Bago | Burmans |
| 5 | Burman666 | G2a1 | 136 223 227 278 362 | 73 263 309+C 315+C | 16012-16569/1-423 | | + | + | | | | | | Bago | Burmans |
| 5 | Burman667 | F1 | 189 284 304 362 519 | 73 146 249d 263 315+C 522-523d | 16011-16569/1-586 | | | | | - | | | | Bago | Burmans |
| 5 | Burman668 | F1c | 111 129 304 519 | 73 152 234 249d 263 315+C 522-523d | 16018-16569/1-586 | | | | | - | | | | Bago | Burmans |
| 5 | Burman669 | F2a | 092A 189 243 291 304 | 73 207 249d 263 309+C 315+C | 16013-16569/1-316 | | | | | + | | | | Bago | Burmans |
| 5 | Burman670 | F2a | 092A 189 243 291 304 | 73 207 249d 263 309+C 315+C | 16041-16569/1-316 | | | | | + | | | | Bago | Burmans |
| 5 | Burman671 | G2a | 129 223 278 362 | 73 150 263 309+C 315+C 489 | 16059-16569/1-555 | | | | | | | | 4520-5175=4769 4833 4967 5108 | Bago | Burmans |
| 5 | Burman672 | M49 | 223 234 302 519 | 73 263 309+C 315+C 489 498d | 16043-16569/1-596 | | | | | | | | 3669-4200=3780 | Bago | Burmans |
| 5 | Burman673* | M76 | 124 183C 189 278 293C 326 362 | 73 263 309+CC | 16000-16569/1-315 | | | | | | | | | Bago | Burmans |
| 5 | Burman674 | A | 223 290 319 362 | 73 151 152 200 235 263 315+C 522-523d | 16024-16569/1-596 | | | | | | | | | Bago | Burmans |
| 5 | Burman675* | M21a | 129 223 256 526 | 73 263 309+C 315+C 489 | 16014-16569/1-586 | | | | | | | | 15011-15614=15043 15301 15326 | Bago | Burmans |
| 5 | Burman676 | B5a | 140 183C 189 266A 519 | 73 146 210 263 309+CC 315+C | 16012-16569/1-362 | + | | | | | | | | Bago | Burmans |
| 5 | Burman677 | D | 174 223 257 311 362 | 73 152 263 315+C 489 | 16012-16569/1-599 | | - | - | | | | | | Bago | Burmans |
| 5 | Burman678 | N21 | 093 182 193 223 260 519 | 73 150 195 263 309+C 315+C 337d | 16011-16569/1-599 | | | | | | | | 13107-13820=13437 | Bago | Burmans |
| 5 | Burman679 | F1 | 183C 189 519 | 73 249d 263 263 309+C 315+C | 16012-16569/1-323 | | | | | - | | | | Bago | Burmans |
| 5 | Burman680 | A | 223 256 290 319 362 | 73 151 152 200 235 263 315+C 522-523d | 16018-16569/1-596 | | | | | | | | | Bago | Burmans |
| 5 | Burman681* | M45 | 223 519 | 73 146 152 263 309+C 315+C 489 | 16016-16569/1-596 | | | | | | | | | Bago | Burmans |
| 5 | Burman682 | F1a | 058T 129 169 172 304 519 | 73 249d 263 309+CC 315+C 522-523d | 16012-16569/1-596 | | | | | - | | | | Bago | Burmans |
| 5 | Burman683 | M38 | 189 223 256 519 | 73 239 246 263 309+C 315+C 489 | 16027-16569/1-595 | | | | | | | | 15011-15650=15043 15262 15301 15314 15326 15487 | Bago | Burmans |
| 5 | Burman684 | C | 183C 189 223 249 298 327 357 519 | 73 249d 263 309+CC | 16045-16569/1-309 | | | | | | + | | | Bago | Burmans |
| 5 | Burman685 | A | 223 290 319 362 | 73 151 152 200 235 263 315+C 522-523d | 16018-16569/1-596 | | | | | | | | | Bago | Burmans |
| 5 | Burman686 | M49 | 223 234 302 519 | 73 263 309+C 315+C | 16012-16569/1-316 | | | | | | | | 3677-4020=3780 | Bago | Burmans |
| 5 | Burman687 | F1c | 111 129 266 304 519 | 73 152 249d 263 309+C 315+C | 16018-16569/1-316 | | | | | - | | | | Bago | Burmans |
| 5 | Burman688 | F1c | 111 129 189 304 519 | 73 152 185 234 249d 263 315+C 522-523d | 16017-16569/1-598 | | | | | - | | | | Bago | Burmans |
| 5 | Burman689* | R21 | 172 182C 183C 189 278 356 399 519 | 73 263 309+C 315+C 455+T 460 463+CC | 16018-16569/1-464 | | | | | | | | 12059-12782=rCRS; 14614-14700=rCRS | Bago | Burmans |
| 5 | Burman690 | M20 | 086 129 209 223 272 519 | 73 152 225 249d 263 315+C 316 489 522-523d | 16008-16569/1-588 | | | | | | | | 12036-12513=12354; 14072-14700=14110 | Bago | Burmans |
| 5 | Burman691* | M38 | 189 223 256 519 | 73 239 246 263 309+C 315+C 489 | 16021-16569/1-573 | | | | | | | | | Bago | Burmans |
| 5 | Burman692 | F1a1a | 093 108 129 162 172 259A 304 519 | 73 249d 263 315+C 489 522-523d | 16075-16569/1-596 | | | | | - | | | | Bago | Burmans |
| 5 | Burman693 | F1a | 129 172 304 519 | 73 249d 263 309+C 315+C 522-523d | 16043-16569/1-586 | | | | | - | | | | Bago | Burmans |
| 5 | Burman694 | B5a | 140 182C 183C 189 250 261 266A 519 | 73 152 210 263 309+CCC 315+C | 16043-16569/1-316 | + | | | | | | | | Bago | Burmans |
| 5 | Burman695 | A | 223 256 290 319 362 | 73 151 152 200 235 263 315+C 522-523d | 16013-16569/1-598 | | | | | | | | | Bago | Burmans |
| 5 | Burman696 | M7b1 | 129 192 223 297 | 73 150 199 263 309+C 315+C 489 | 16035-16569/1-488 | | | | + | | | | | Bago | Burmans |
| 5 | Burman697 | F1 | 189 284 304 362 519 | 73 146 249d 263 315+C | 16086-16569/1-496 | | | | | - | | | | Bago | Burmans |
| 5 | Burman698 | A | 223 290 319 362 | 73 151 152 200 235 263 315+C 522-523d | 16012-16569/1-586 | | | | | | | | | Bago | Burmans |
| 5 | Burman699 | M74 | 093 146 223 311 362 519 | 73 263 315+C 489 | 16016-16569/1-539 | | | | | | | | 4517-5214=4769 5054; 15007-15734=15043 15301 15326 | Bago | Burmans |
| 5 | Burman700 | M9a1b1 | 158 223 234 362 519 | 73 150 152 | 16017-16569/1-161 | | | | | | | | 4244-4961=4491 4769 | Bago | Burmans |
| 5 | Burman701 | F1c | 111 129 189 304 519 | 73 152 185 234 249d 263 315+C | 16013-16569/1-316 | | | | | - | | | | Bago | Burmans |
| 5 | Burman702* | M24 | 075 223 311 327 519 | 73 146 152 195 263 315+C | 16021-16569/1-316 | | | | | | | | | Bago | Burmans |
| 5 | Burman703 | F1a1a | 093 108 129 162 172 259A 304 519 | 73 249d 263 315+C 482 522-523d | 16010-16569/1-574 | | | | | - | | | | Bago | Burmans |
| 5 | Burman704 | F1a1a | 093 108 129 162 272 259A 304 519 | 73 249d 263 315+C 482 522-523d | 16018-16569/1-574 | | | | | - | | | | Bago | Burmans |
| 5 | Burman705 | M5a2a1 | 129 223 264 265C 311 519 | 73 263 309+C 315+C | 16012-16569/1-316 | | | | | | | | 4520-5223=4769; 15018-15659=15043 15262 15301 15326 | Bago | Burmans |
| 5 | Burman706 | M10 | 223 254 311 519 | 73 94 195 263 309+C 315+C | 16001-16569/1-396 | | | | | | | | 15625-16240=15924 16223 | Bago | Burmans |
| 5 | Burman707 | F1c | 111 129 266 304 519 | 73 152 249d 263 309+C 315+C 522-523d | 16001-16569/1-574 | | | | | - | | | | Bago | Burmans |
| 5 | Burman708 | R22a | 169 224 249 265C 288 291 304 519 | 73 152 199 263 315+C 329 | 16001-16569/1-460 | | | | | | | | 12599-12818=12609; 13084-13383=13359 | Bago | Burmans |
| 5 | Burman709 | M20 | 086 129 209 223 272 519 | 73 152 225 249d 263 315+C 316 489 | 16012-16569/1-510 | | | | | | | | 12040-12433=12354; 14075-14254=14110? | Bago | Burmans |
| 5 | Burman710 | F1 | 189 284 304 362 519 | 73 146 249d 263 315+C 522-523d | 16020-16569/1-574 | | | | | - | | | | Bago | Burmans |
| 5 | Burman711 | R22a | 169 224 249 265C 288 291 304 519 | 73 152 199 263 315+C 329 | 16021-16569/1-574 | | | | | | | | 12595-12966=12609; 13084-13375=13359 | Bago | Burmans |
| 5 | Burman712* | M5a2a1 | 129 223 264 265C 311 519 | 73 263 309+C 315+C 489 | 16018-16569/1-574 | | | | | | | | | Bago | Burmans |
| 5 | Burman713 | B5a | 140 182C 183C 189 261 266A 304 519 | 73 152 210 263 309+CC 315+C 522-523d | 16013-16569/1-574 | + | | | | | | | | Bago | Burmans |
| 5 | Burman714 | M38 | 189 223 256 519 | 73 239 246 263 309+C 315+C | 16001-16569/1-316 | | | | | | | | 15011-15679=15043 15262 15301 15314 15326 15487 | Bago | Burmans |

| Code | Sample nam | Haplogroup | HVS-I (16000+) | HVS-II | Readable Region | 9bp | 4831*Hha*1 | 5176*Alu*1 | 9820*Hinf*1 | 12406*Hpa*1 | 13262*Alu*1 | 14465*Acc*1 | Coding-Region Polymorphisms | Location | Nation |
|---|---|---|---|---|---|---|---|---|---|---|---|---|---|---|---|
| 5 | Burman715 | D5 | 189 223 362 519 | 73 150 309+CC 315+C | 16021-16569/1-316 | | - | - | | | | | | Bago | Burmans |
| 5 | Burman716* | M54 | 145 172 188 189 192 223 293 304 318 519 | 73 263 315+C 489 | 16021-16569/1-574 | | | | | | | | | Bago | Burmans |
| 5 | Burman717 | R22a | 169 224 249 265C 288 291 304 519 | 73 152 199 263 315+C 329 | 16012-16569/1-347 | | | | | | | | 12595-12958=12609; 13078-13541=13359 | Bago | Burmans |
| 5 | Burman718 | D4 | 201 223 362 | 73 152 263 315+C 489 522-523d | 16011-16569/1-574 | | - | - | | | | | | Bago | Burmans |
| 6 | Burman719 | M7h | 086 129 223 311 | 73 200 263 315+C 489 | 16043-16569/1-585 | | | | | | | | 15012-15731=15043 15301 15326 15676 | Ayeyarwady | Burmans |
| 6 | Burman720 | M58 | 183 189 193d 223 266 295 519 | 73 143 153 263 309+C 315+C 489 | 16050-16569/1-510 | | | | | | | | 15617-16192=15924 16183 16189 | Ayeyarwady | Burmans |
| 6 | Burman721 | M12 | 129 172 223 290 519 | 73 152 198 263 309+CC 315+C 489 522-523d | 16006-16569/1-575 | | | | | | | | 14618-15055=14727 14766 14783 15010 15043 | Ayeyarwady | Burmans |
| 6 | Burman722* | M60a | 223 284 319 519 | 73 263 315+C 489 522-523d | 16023-16569/1-596 | | | | | | | | | Ayeyarwady | Burmans |
| 6 | Burman723* | M35b | 223 519 | 73 199 263 315+C 489 | 16032-16569/1-495 | | | | - | | | | 6358-6876=rCRS | Ayeyarwady | Burmans |
| 6 | Burman724 | M12 | 129 172 223 290 519 | 73 152 198 263 309+CC 315+C | 16032-16569/1-328 | | | | | | | | 14615-15490=14727 14766 14783 15010 15043 15301 15326 | Ayeyarwady | Burmans |
| 6 | Burman725 | M7h | 086 129 223 311 | 73 263 315+C 489 | 16012-16569/1-504 | | | | | | | | 12005-12435=12405; 15001-15770=15043 15301 15326 15676 | Ayeyarwady | Burmans |
| 6 | Burman726 | M12 | 129 172 223 290 519 | 73 152 198 263 309+CC 315+C | 16013-16569/1-316 | | | | | | | | 14620-15530=14727 14766 14783 15010 15043 15301 15326 | Ayeyarwady | Burmans |
| 6 | Burman727 | M2c | 182C 183C 189 223 227 258C 263 | 73 263 447G 489 | 16007-16569/1-552 | | | | | | | | 15410-15986=15670 15929 | Ayeyarwady | Burmans |
| 6 | Burman728* | M49 | 153 213 223 234 257 294 519 527 | 73 263 309+C 315+C 489 | 16012-16569/1-504 | | | | | | | | | Ayeyarwady | Burmans |
| 6 | Burman729 | M38 | 092 111 184 223 519 | 73 246 263 315+C 489 | 16010-16569/1-597 | | | | | | | | 15011-15600=15043 15301 15314 15326 15487 | Ayeyarwady | Burmans |
| 6 | Burman730 | B4c2 | 183C 184A 189 217 235 519 | 73 263 309+CC 315+C | 16012-16569/1-506 | + | | | | | | | | Ayeyarwady | Burmans |
| 6 | Burman731 | M7h | 086 129 223 311 | 73 263 315+C 485+C 486+A 487 | 16018-16569/1-598 | | | | | | | | 9818-10074=9824; 12058-12435=12405; 15010-15312=15043 15301 | Ayeyarwady | Burmans |
| 6 | Burman732* | M24 | 086 223 278 519 | 73 146 195 263 309+C 315+C | 16032-16569/1-316 | | | | | | | | 5831-6103=rCRS | Ayeyarwady | Burmans |
| 6 | Burman733 | M7h | 086 129 223 311 | 73 263 315+C 489 | 16012-16569/1-599 | | | | | | | | 9824-9978=rCRS (No 9824?); 15015-15700=15043 15301 15326 15676 | Ayeyarwady | Burmans |
| 6 | Burman734 | D | 192 223 256 311 362 519 | 73 146 200 228 263 309+C 315+C 489 | 16017-16569/1-600 | | | | | | | | 4520-5118=4769 4883; 15010-15633=15043 15301 15326 | Ayeyarwady | Burmans |
| 6 | Burman735 | D | 192 223 256 311 362 519 | 73 146 200 228 263 309+C 315+C 489 | 16023-16569/1-515 | | | | | | | | 4520-5250=4769 4883 5178A; 15011-15780=15043 15301 15326 | Ayeyarwady | Burmans |
| 6 | Burman736 | M7h | 086 129 223 311 | 73 263 315+C 489 | 16020-16569/1-599 | | | | | | | | 9817-10153=9824; 12058-12432=12405; 15005-15259=15043 | Ayeyarwady | Burmans |
| 6 | Burman737 | D | 192 223 256 311 362 519 | 73 146 200 228 263 309+C 315+C 489 | 16008-16569/1-515 | | | | | | | | 4519-5111=4769 4883; 15011-15782=15043 15301 15326 | Ayeyarwady | Burmans |
| 6 | Burman738 | M7h | 086 129 223 311 | 73 263 315+C 489 | 16012-16569/1-566 | | | | | | | | 9817-10030=9824; 12060-12417=12405; 15011-15870=15043 15301 15326 15676 | Ayeyarwady | Burmans |
| 6 | Burman739 | R9b | 124 148 304 309 327 390 519 | 73 263 309+C 315+C | 16018-16569/1-563 | | | | | | | | | Ayeyarwady | Burmans |
| 6 | Burman740 | D5a2 | 092 172 182C 183C 189 223 266 362 | 73 150 263 309+C 315+C | 16016-16569/1-316 | | - | - | | | | | | Ayeyarwady | Burmans |
| 6 | Burman741 | M9a1b1 | 086 158 223 234 356 362 519 | 73 150 152 153 263 315+C 489 | 16012-16569/1-597 | | | | | | | | 4237-4961=4491 4769 | Ayeyarwady | Burmans |
| 6 | Burman742 | G | 174 223 239 362 | 73 214 263 315+C 356+C 489 | 16012-16569/1-539 | | + | + | | | | | | Ayeyarwady | Burmans |
| 6 | Burman743 | M7h | 086 129 223 311 | 73 263 315+C 489 | 16012-16569/1-590 | | | | | | | | 9812-12027=9824; 12060-12425=12405; 15008-15817=15043 15301 15326 15676 | Ayeyarwady | Burmans |
| 6 | Burman744 | N21 | 093 182 193 223 260 519 | 73 150 195 263 309+C 315+C 337d | 16012-16569/1-460 | | | | | | | | 13084-13672=13437 | Ayeyarwady | Burmans |
| 6 | Burman745 | M58 | 183 189 223 266 295 519 | 73 143 153 263 309+C 315+C 489 | 16012-16569/1-505 | | | | | | | | 15625-16200=15924 16183 16189 | Ayeyarwady | Burmans |
| 6 | Burman746 | U2 | 051 114A | 73 146 215 263 309+C 315+C | 16017-16569/1-563 | | | | | | | | 12058-12284=12106 | Ayeyarwady | Burmans |
| 6 | Burman747 | M7h | 086 129 223 311 | 73 263 315+C 489 | 16012-16569/1-593 | | | | | | | | 9817-10171=9824; 12059-12203=rCRS; 15008-15699=15043 15301 15326 15676 | Ayeyarwady | Burmans |
| 6 | Burman748* | M4a | 145 176 223 232 261 311 519 | 73 146 194 263 309+C 315+C 489 | 16010-16569/1-566 | | | | | | | | | Ayeyarwady | Burmans |
| 6 | Burman749 | A | 223 274 290 319 362 527 | 73 152 235 263 315+C 522-523d | 16013-16569/1-591 | | | | | | | | | Ayeyarwady | Burmans |
| 6 | Burman750 | M7h | 086 129 223 311 | 73 263 315+C 489 | 16012-16569/1-592 | | | | | | | | 9817-10210=9824; 12059-12420=12405; 15017-15633=15043 15301 15326 | Ayeyarwady | Burmans |
| 6 | Burman751 | M7b | 129 223 297 357 | 73 150 199 263 315+C 489 | 16012-16569/1-592 | | | | + | | | | | Ayeyarwady | Burmans |
| 6 | Burman752 | M7h | 086 129 223 311 | 73 263 315+C 489 | 16012-16569/1-513 | | | | | | | | 9817-10256=9824; 12058-12541=12405; 15010-15473=15043 15301 15326 | Ayeyarwady | Burmans |
| 6 | Burman753 | B4c2 | 183C 184A 189 217 235 519 | 73 263 309+CC 315+C | 16023-16569/1-316 | + | | | | | | | | Ayeyarwady | Burmans |
| 6 | Burman754 | G | 174 223 239 362 | 73 214 263 315+C 356+C 489 | 16008-16569/1-504 | | + | + | | | | | | Ayeyarwady | Burmans |
| 6 | Burman755* | M24 | 086 223 278 519 | 73 146 195 263 315+C | 16022-16569/1-476 | | | | | | | | | Ayeyarwady | Burmans |
| 6 | Burman756 | M7h | 086 129 223 311 | 73 263 315+C 489 | 16017-16569/1-579 | | | | | | | | 12058-12407=12405?; 15011-15864=15043 15301 15326 15676 | Ayeyarwady | Burmans |
| 6 | Burman757 | M9a1b1 | 086 158 223 234 356 362 519 | 73 150 152 153 263 | 16012-16569/1-275 | | | | | | | | 4242-4877=4491 4769 | Ayeyarwady | Burmans |
| 6 | Burman758 | R9b | 124 148 304 309 327 390 519 | 73 263 309+C 315+C | 16012-16569/1-563 | | | | | | | | 12593-13177=12714 | Ayeyarwady | Burmans |
| 6 | Burman759 | M38 | 092 111 184 223 519 | 73 246 263 315+C 489 | 16012-16569/1-575 | | | | | | | | 15012-15613=15043 15301 15314 15326 15487 | Ayeyarwady | Burmans |
| 6 | Burman760 | M9a1b1 | 158 223 234 362 519 | 73 150 152 153 263 315+C 489 | 16009-16569/1-563 | | | | | | | | 4238-4907=4491 4769 | Ayeyarwady | Burmans |
| 6 | Burman761 | D4 | 189 201 223 362 | 73 152 234 263 309+C 315+C 489 | 16007-16569/1-574 | | - | - | | | | | | Ayeyarwady | Burmans |
| 6 | Burman762 | M49 | 153 213 223 234 257 294 519 527 | 73 263 309+C 315+C 489 | 16032-16569/1-504 | | | | | | | | 3682-3829=3780 | Ayeyarwady | Burmans |
| 6 | Burman763 | M58 | 183 189 223 266 295 519 | 73 143 153 263 309+C 3159+C | 16033-16569/1-316 | | | | | | | | 15616-16192=15924 16183 16189 | Ayeyarwady | Burmans |
| 6 | Burman764 | M20 | 086 129 209 223 272 519 | 73 152 225 249d 263 309+C 315+C 316 | 16017-16569/1-352 | | | | | | | | 12045-12396=12354; 14086-14460=14110 | Ayeyarwady | Burmans |
| 6 | Burman765 | M7h | 086 129 223 311 | 73 263 315+C 489 | 16008-16569/1-565 | | | | | | | | 9814-10273=9824; 12059-12432=12405; 15012-15861=15043 15301 15326 15676 | Ayeyarwady | Burmans |
| 6 | Burman766 | M7h | 086 129 223 311 | 73 263 315+C 489 | 16014-16569/1-589 | | | | | | | | 9817-10188=9824; 12059-12430=12405; 15011-15752=15043 15301 15326 15676 | Ayeyarwady | Burmans |
| 6 | Burman767 | M83 | 311 319 357 | 73 152 263 315+C 356+C | 16038-16569/1-468 | | | | | | | | 15412-15998=15670 15941 | Ayeyarwady | Burmans |
| 6 | Burman768 | M7h | 086 129 223 311 | 73 263 315+C 489 | 16014-16569/1-600 | | | | | | | | 9817-10160=9824; 12060-12534=12405; 15011-15731=15043 15301 15326 15676 | Ayeyarwady | Burmans |
| 6 | Burman769 | M7h | 086 129 223 311 | 73 263 315+C 489 | 16019-16569/1-565 | | | | | | | | 9817-9978=9824; 12058-12450=12405; 15011-15789=15043 15301 15326 15676 | Ayeyarwady | Burmans |
| 6 | Burman770 | M7h | 086 129 223 311 | 73 263 315+C 489 | 16016-16569/1-590 | | | | | | | | 9819-10021=9824; 12059-12440=12405; 15011-15176=15043 | Ayeyarwady | Burmans |
| 6 | Burman771 | F1a | 129 172 304 519 | 73 152 249d 263 315+C 522-523d 573+C | 16013-16569/1-596 | | | | | - | | | | Ayeyarwady | Burmans |
| 6 | Burman772 | M7h | 086 129 223 311 | 73 263 315+C 489 | 16012-16569/1-566 | | | | | | | | 9816-9941=9824; 12058-12439=12405; 15011-15821=15043 15301 15326 15676 | Ayeyarwady | Burmans |
| 6 | Burman773 | F1 | 183C 189 213 304 519 | 73 249d 263 309+CC 315+C | 16012-16569/1-316 | | | | | - | | | | Ayeyarwady | Burmans |
| 6 | Burman774 | F1 | 183C 189 213 304 519 | 73 249d 263 309+CC 315+C | 16012-16569/1-316 | | | | | - | | | | Ayeyarwady | Burmans |
| 6 | Burman775 | M83 | 311 319 357 | 73 152 263 315+C 356+C 489 | 16029-16569/1-590 | | | | | | | | 15426-16056=15670 15941 | Ayeyarwady | Burmans |
| 6 | Burman776 | G | 174 223 239 362 | 73 214 263 315+C 356+C 489 | 16012-16569/1-573 | | + | + | | | | | | Ayeyarwady | Burmans |
| 6 | Burman777 | M7h | 086 129 223 311 | 73 263 315+C 489 | 16012-16569/1-593 | | | | | | | | 12060-12435=12405; 15012-15697=15043 15301 15326 15676 | Ayeyarwady | Burmans |
| 6 | Burman778 | F1 | 183C 189 213 304 519 | 73 249d 263 309+CC 315+C | 16023-16569/1-316 | | | | | - | | | | Ayeyarwady | Burmans |
| 6 | Burman780 | M7h | 086 129 223 311 | 73 263 315+C 489 | 16013-16569/1-574 | | | | | | | | 9817-9911=9824; 15012-15763=15043 15301 15326 15676 | Ayeyarwady | Burmans |

| Code | Sample nam | Haplogroup | HVS-I (16000+) | HVS-II | Readable Region | 9bp | 4831*HhaI* | 5176*AluI* | 9820*HinfI* | 12406*HpaI* | 13262*AluI* | 14465*AccI* | Coding-Region Polymorphisms | Location | Nation |
|---|---|---|---|---|---|---|---|---|---|---|---|---|---|---|---|
| 6 | Burman781 | M7b1 | 129 192 223 297 | 73 150 199 263 309+C 315+C 489 | 16029-16569/1-572 | | | | + | | | | | Ayeyarwady | Burmans |
| 6 | Burman782 | R9b | 124 148 304 309 327 390 519 | 73 263 309+C 315+C | 16014-16569/1-574 | | | | | | | | 12600-12754=12714 | Ayeyarwady | Burmans |
| 6 | Burman783 | D4 | 192 223 256 311 362 519 | 73 146 200 228 263 309+C 315+C 489 | 16013-16569/1-574 | | - | - | | | | | | Ayeyarwady | Burmans |
| 6 | Burman784 | M49 | 223 234 519 | 73 263 309+C 315+C 489 | 16012-16569/1-574 | | | | | | | | 3678-4281=3780 | Ayeyarwady | Burmans |
| 6 | Burman785 | M12 | 129 172 223 290 519 | 73 152 198 263 309+C 315+C | 16018-16569/1-316 | | | | | | | | 14613-15300=14727 14766 14783 15010 15043 | Ayeyarwady | Burmans |
| 6 | Burman786 | B4c2 | 183C 184A 189 217 235 519 | 73 263 309+CC 315+C | 16013-16569/1-574 | + | | | | | | | | Ayeyarwady | Burmans |
| 6 | Burman787 | M38 | 092 111 184 223 519 | 73 246 263 315+C 489 | 16013-16569/1-574 | | | | | | | | 15003-15571=15043 15301 15314 15326 15487 | Ayeyarwady | Burmans |
| 6 | Burman788 | G2a1 | 136 223 227 278 362 | 73 263 309+C 315+C 489 523+CA | 16014-16569/1-574 | | + | + | | | | | | Ayeyarwady | Burmans |
| 6 | Burman789 | D5a2 | 092 164 182C 183C 189 223 266 362 | 73 150 228 263 309+CC 315+C 363? 489 522-523d | 16012-16569/1-574 | | - | - | | | | | | Ayeyarwady | Burmans |
| 6 | Burman790 | A | 214 223 290 311G 319 362 | 73 215 235 263 315+C 522-523d | 16012-16569/1-574 | | | | | | | | | Ayeyarwady | Burmans |
| 6 | Burman791 | M7a | 093 145 169 209 223 266 317T 324 362 519 | 73 146 199 263 309+C 315+C 489 520 | 16013-16569/1-574 | | | | + | | | | | Ayeyarwady | Burmans |
| 7 | Burman118 | C | 223 298 327 519 | 73 146 249d 263 315+C 489 | 16014-16569/1-575 | | | | | - | | | 13076-13307=13236 | Chin | Chin |
| 7 | Burman119 | D | 209 223 362 519 | 73 263 315+C 489 522-523d | 16018-16569/1-575 | | - | - | | | | | | Chin | Chin |
| 7 | Burman120 | D | 209 223 362 519 | 73 263 315+C | 16007-16569/1-316 | | - | - | | | | | | Chin | Chin |
| 7 | Burman121 | F1 | 189 304 311 519 | 73 146 249d 263 309+C 315+C 522-523d | 16007-16569/1-575 | | | | | - | | | | Chin | Chin |
| 7 | Burman122 | F1 | 189 304 311 519 | 73 146 249d 263 309+C 315+C | 16007-16569/1-494 | | | | | - | | | | Chin | Chin |
| 7 | Burman123 | M33b | 093 223 266 324 355 362 391 519 | 73 146 263 309+C 315+C 489 | 16012-16569/1-575 | | | | | | | | 3200-3675=3221 | Chin | Chin |
| 7 | Burman124 | C | 223 298 327 519 | 73 146 249d 263 315+C 489 | 16012-16569/1-533 | | | | | | | | 13084-13513=13263 | Chin | Chin |
| 7 | Burman125 | M84 | 223 258d 272 519 | 73 185 263 315+C 489 | 16015-16569/1-575 | | | | | | | | 11033-11817=11191 11719 | Chin | Chin |
| 7 | Burman126 | F1 | 189 304 311 519 | 73 146 249d 263 309+C 315+C 522-523d | 16032-16569/1-575 | | | | | | | | 6365-6860=6392 | Chin | Chin |
| 7 | Burman127 | D4 | 209 223 362 519 | 73 263 315+C 489 522-523d | 16015-16569/1-575 | | | | | | | | 11003-11756=11719; 14076-14697=14668 | Chin | Chin |
| 7 | Burman128 | F1 | 304 311 519 | 73 146 249d 263 309+C 315+C 522-523d 549 | 16224-16569/1-563 | | | | | | | | 11003-11814=11440 11719; 14074-14703=rCRS | Chin | Chin |
| 7 | Burman129 | C | 223 298 327 519 | 73 146 249d 263 315+C 489 | 16013-16569/1-575 | | | | | | | | 11022-11875=11719; 13076-13664=rCRS; 14078-14689=14318 | Chin | Chin |
| 7 | Burman130 | D4 | 209 223 362 519 | 73 152H 263 315+C | 16010-16569/1-408 | | | | | | | | 11022-11775=11719; 14072-14694=14668 | Chin | Chin |
| 7 | Burman131* | M84 | 093 223 258d 272 519 | 73 185 188 195 263 315+C | 16008-16569/1-371 | | | | | | | | | Chin | Chin |
| 7 | Burman132 | F1 | 189 304 311 400 519 | 73 146 249d 263 315+C | 16021-16569/1-400 | | | | | - | | | | Chin | Chin |
| 7 | Burman133 | F1 | 189 304 311 519 | 73 146 249d 263 309+C 315+C | 16023-16569/1-316 | | | | | - | | | | Chin | Chin |
| 7 | Burman134 | F1 | 189 304 311 519 | 73 146 249d 263 309+C 315+C | 16012-16569/1-316 | | | | | - | | | | Chin | Chin |
| 7 | Burman135 | M33b | 093 223 266 324 355 362 391 519 | 73 146 263 309+C 315+C 489 | 16012-16569/1-504 | | | | | | | | 3202-3676=3221 | Chin | Chin |
| 7 | Burman136 | M84 | 223 258d 272 519 | 73 185 263 315+C 489 | 16004-16569/1-575 | | | | | | | | 1529-1839=1719 | Chin | Chin |
| 7 | Burman137 | D | 092 223 311 362 519 | 73 94 214 262 263 315+C 489 | 16019-16569/1-574 | | | | | | | | 4520-5105=4769 4883 | Chin | Chin |
| 7 | Burman138 | M84 | 223 258d 272 519 | 73 185 263 315+C 489 | 16014-16569/1-575 | | | | | | | | 1634-1762=1719 | Chin | Chin |
| 7 | Burman139 | M84 | 223 258d 272 519 | 73 185 263 315+C 489 | 16011-16569/1-504 | | | | | | | | 1703-1731=1719 | Chin | Chin |
| 7 | Burman140 | F1 | 189 304 311 519 | 73 146 249d 263 309+CC 315+C | 16012-16569/1-316 | | | | | - | | | | Chin | Chin |
| 7 | Burman141 | M9a1a2 | 145 223 234 316 519 | 73 153 263 309+C 315+C | 16022-16569/1-316 | | | | | | | | 4243-4875=4491 4769 | Chin | Chin |
| 7 | Burman142 | F1 | 189 304 311 519 | 73 146 249d 263 309+CC 315+C | 16018-16569/1-316 | | | | | - | | | | Chin | Chin |
| 7 | Burman143 | D | 092 223 311 362 | 73 94 262 263 315+C 489 | 16012-16569/1-575 | | | | | | | | 4628-4912=4769 4883 | Chin | Chin |
| 7 | Burman144 | M33b | 093 223 266 324 355 362 391 519 | 73 146 263 | 16012-16569/1-290 | | | | | | | | 3217-3627=3221; 11033-11845=11719; 14084-14323=rCRS | Chin | Chin |
| 7 | Burman145 | A | 223 274 290 304 319 356 362 | 73 152 235 263 309+CC 315+C 469+A 522-523d 549 | 16012-16569/1-486 | | | | | | | | | Chin | Chin |
| 7 | Burman146 | M55 | 136 217 223 319 381 | 73 94 173 204 263 315+C 469+A 482 489 | 16012-16569/1-575 | | | | | | | | 9243-9539=9447 | Chin | Chin |
| 7 | Burman147 | A | 223 290 319 362 519 | 73 151 152 200 235 263 315+C 469+A 522-523d | 16013-16569/1-575 | | | | | | | | | Chin | Chin |
| 7 | Burman148 | F1 | 189 304 311 519 | 73 146 249d 263 309+C 315+C | 16008-16569/1-366 | | | | | | | | 6364-6640=6392 | Chin | Chin |
| 7 | Burman149 | Z | 185 189 223 260 298 519 | 73 152 249d 263 315+C 489 | 16013-16569/1-575 | | | | | | | | | Chin | Chin |
| 7 | Burman150 | M84 | 223 258d 272 519 | 73 185 263 315+C 489 | 16007-16569/1-575 | | | | | | | | 1502-2000=1719 1809 | Chin | Chin |
| 7 | Burman151 | M84 | 223 258d 272 519 | 73 185 263 315+C | 16026-16569/1-316 | | | | | | | | 1513-1900=1719 1809 | Chin | Chin |
| 7 | Burman152 | D | 092 223 311 362 | 73 94 262 263 315+C | 16015-16569/1-463 | | | | | | | | 4519-5223=4769 4883 5178A | Chin | Chin |
| 7 | Burman153 | C | 223 298 327 519 | 73 146 249d 263 315+C 489 | 16021-16569/1-498 | | | | | | + | | | Chin | Chin |
| 7 | Burman154 | M33b | 093 223 266 324 355 362 391 519 | 73 146 263 309+C 315+C 489 | 16011-16569/1-575 | | | | | | | | 3213-3652=3221 | Chin | Chin |
| 7 | Burman155 | F1 | 189 304 311 519 | 73 146 234 249d 263 309+CC 315+C | 16020-16569/1-316 | | | | | - | | | | Chin | Chin |
| 7 | Burman156 | M33b | 093 223 266 324 355 362 391 519 | 73 146 263 309+C 315+C | 16013-16569/1-316 | | | | | | | | 3200-3408=3221 | Chin | Chin |
| 7 | Burman157 | M84 | 223 258d 272 519 | 73 185 263 315+C | 16019-16522/45-316 | | | | | | | | 1624-1764=1719 | Chin | Chin |
| 7 | Burman158 | M84 | 223 258d 272 519 | 73 185 263 315+C | 16022-16569/1-316 | | | | | | | | 1690-1856=1719 1809 | Chin | Chin |
| 7 | Burman159 | M84 | 223 258d 272 519 | 73 185 263 315+C 489 | 16012-16569/1-575 | | | | | | | | | Chin | Chin |
| 7 | Burman160 | F1 | 189 304 311 519 | 73 146 249d 263 309+C 315+C 522-523d | 16012-16569/1-575 | | | | | - | | | | Chin | Chin |
| 7 | Burman161 | M9a | 223 234 362 519 | 73 150 152 153 263 315+C 489 522-523d | 16015-16569/1-575 | | | | | | | | 4237-4803=4491 4769 | Chin | Chin |
| 7 | Burman162 | C | 223 298 327 519 | 73 146 249d 263 315+C 489 | 16021-16569/1-575 | | | | | | + | | | Chin | Chin |
| 7 | Burman163 | C | 223 298 327 519 | 73 146 249d 263 315+C 489 | 16012-16569/1-575 | | | | | | + | | | Chin | Chin |
| 7 | Burman164 | M55 | 136 217 223 319 381 | 73 94 173 204 263 315+C 482 489 | 16018-16569/1-544 | | | | | | | | 9250-9551=9447 9540 | Chin | Chin |
| 7 | Burman165 | M84 | 223 258d 272 519 | 73 185 263 315+C 489 | 16013-16569/1-537 | | | | | | | | 1624-1936=1719 1809 | Chin | Chin |
| 7 | Burman166 | M84 | 223 258d 272 519 | 73 185 263 315+C | 16021-16569/1-316 | | | | | | | | 1563-1897=1719 1809 | Chin | Chin |
| 7 | Burman167 | D | 092 223 311 362 | 73 94 262 263 315+C | 16019-16569/1-316 | | | | | | | | 4519-5211=4769 4883 5178A; 15006-15673=15043 15301 15326 | Chin | Chin |
| 7 | Burman168 | M84 | 093 223 258d 272 519 | 73 185 188 195 263 315+C 489 | 16021-16569/1-575 | | | | | | | | 1592-1929=1719 1809 | Chin | Chin |
| 7 | Burman169 | D | 092 223 311 362 | 73 94 262 263 315+C 489 | 16023-16569/1-575 | | | | | | | | 4530-4912=4769 4883; 15002-15674=15043 15301 15326 | Chin | Chin |
| 7 | Burman170* | M84 | 223 247 258d 272 519 | 73 185 263 315+C 456 489 | 16013-16569/1-503 | | | | | | | | 1626-1962=rCRS | Chin | Chin |
| 7 | Burman171* | M84 | 223 258C 272 519 | 73 185 263 309+C 315+C 489 522-523d | 16013-16569/1-575 | | | | | | | | 14079-14595=14110 | Chin | Chin |

| Code | Sample nam | Haplogroup | HVS-I (16000+) | HVS-II | Readable Region | 9bp | 4831*HhaI* | 5176*AluI* | 9820*HinfI* | 12406*HpaI* | 13262*AluI* | 14465*AccI* | Coding-Region Polymorphisms | Location | Nation |
|---|---|---|---|---|---|---|---|---|---|---|---|---|---|---|---|
| 7 | Burman172 | M55 | 136 217 223 319 381 | 73 94 173 204 263 315+C 482 489 | 16012-16569/1-572 | | | | | | | | 9217-9660=9447 9540 | Chin | Chin |
| 7 | Burman173 | K | 093 224 311 362 519 | 73 263 315+C 497 523+CA | 16007-16569/1-516 | | | | | | | | | Chin | Chin |
| 7 | Burman174 | M51 | 093 278 519 | 73 152 263 309+C 315+C 356+C 368 489 | 16007-16569/1-511 | | | | | | | | | Chin | Chin |
| 7 | Burman175 | G1 | 051 184 223 290 362 519 | 73 263 309+CC 315+C 489 | 16013-16569/1-517 | | | | | | | | 4520-5212=4769 4833 4841 5108 | Chin | Chin |
| 8 | Burman227 | M51 | 223 278 519 | 73 150 152 263 309+C 315+C 489 | 16007-16569/1-504 | | | | | | | | 14079-14577=14110 14167? 14356? 14527 | Chin | Chin |
| 8 | Burman228 | U2 | 051 110 318 | 73 146 263 523+CA | 16044-16569/1-575 | | | | | | | | 1502-2196=1598 1811 | Chin | Chin |
| 8 | Burman229 | G | 213 223 246T 311 362 519 | 73 195 263 315+C 489 | 16085-16569/1-574 | | + | + | | | | | | Chin | Chin |
| 8 | Burman230 | Z | 185 189 193d 223 260 298 311 362 519 | 73 152 249d 263 315+C 489 | 16007-16569/1-574 | | | | | | | | 8831-9219=8860 9090 | Chin | Chin |
| 8 | Burman231 | A5a | 187 223 290 319 362 | 73 151 152 200 235 263 315+C 522-523d | 16024-16569/1-558 | | | | | | | | | Chin | Chin |
| 8 | Burman232 | F1 | 189 284 304 362 519 | 73 146 152 249d 263 309+C 315+C 523d | 16008-16569/1-574 | | | | | - | | | | Chin | Chin |
| 8 | Burman233* | M84 | 223 258C 262+C 272 519 | 73 185 189 263 315+C 489 | 15015-16569/1-574 | | | | | | | | | Chin | Chin |
| 8 | Burman234 | U2 | 051 318 | 73 146 263 523+CA | 16019-16569/1-575 | | | | | | | | 12058-12484=12106 12308 12372 | Chin | Chin |
| 8 | Burman235* | M84 | 223 258d 272 519 | 73 185 189 199 263 315+C 489 | 16015-16569/1-509 | | | | | | | | 1660-1968=1719 1809 | Chin | Chin |
| 8 | Burman236 | A5a | 187 223 290 319 362 519 | 73 151 152 200 235 263 315+C 522-523d | 16017-16569/1-574 | | | | | | | | | Chin | Chin |
| 8 | Burman237 | D | 092 223 311 362 | 73 94 189 207 214 263 315+C 489 | 16017-16569/1-575 | | | | | | | | 4540-4912=4769 4883; 15012-15746=15043 15301 15326 | Chin | Chin |
| 8 | Burman238 | A11 | 093 223 234 290 293C 319 519 | 73 152 235 263 315+C 522-523d | 16015-16569/1-575 | | | | | | | | | Chin | Chin |
| 8 | Burman239 | F1 | 086 111 304 519 | 73 152 234 249d 263 309+C 315+C 522-523d | 16009-16569/1-573 | | | | | - | | | | Chin | Chin |
| 8 | Burman240 | M49 | 223 234 390 519 | 73 152 249d 263 279 309+CC 315+C 489 | 16004-16569/1-574 | | | | | | | | 3676-4350=3780 | Chin | Chin |
| 8 | Burman241 | A11 | 093 223 234 290 293C 319 519 | 73 152 235 263 315+C 522-523d | 16007-16569/1-574 | | | | | | | | | Chin | Chin |
| 8 | Burman242 | F1 | 189 304 311 519 | 73 146 152 249d 263 309+C 315+C 522-523d | 16007-16569/1-317 | | | | | - | | | | Chin | Chin |
| 8 | Burman243 | M72a | 129 166d 213 214 223 526 | 73 263 315+C 489 | 16007-16569/1-574 | | | | | | | | 15422-16035=15497 15644 15820 | Chin | Chin |
| 8 | Burman244 | G | 213 223 246T 311 362 519 | 73 195 263 309+CC 315+C | 16012-16569/1-317 | | + | + | | | | | | Chin | Chin |
| 8 | Burman245 | F1 | 093 189 304 311 519 | 73 143 146 249d 263 309+C 315+C | 16031-16569/1-317 | | | | | - | | | | Chin | Chin |
| 8 | Burman246 | F1 | 086 111 304 519 | 73 152 234 249d 263 309+C 315+C 522-523d | 16015-16569/1-573 | | | | | - | | | | Chin | Chin |
| 8 | Burman247 | D | 092 223 311 362 | 73 94 189 207 214 263 315+C 489 | 16017-16569/1-574 | | | | | | | | 4520-5529=4769 4883 5178A | Chin | Chin |
| 8 | Burman248 | M49 | 223 234 390 519 | 73 152 249d 263 279 309+CC 315+C | 16013-16569/1-321 | | | | | | | | 3676-4360=3780 | Chin | Chin |
| 8 | Burman249* | M49 | 223 234 390 519 | 73 152 249d 263 279 309+CC 315+C 489 | 16009-16569/1-574 | | | | | | | | | Chin | Chin |
| 8 | Burman250* | M84 | 093 223 258d 272 519 | 73 185 263 315+C 489 | 16012-16569/1-574 | | | | | | | | | Chin | Chin |
| 8 | Burman251 | D | 223 362 | 73 263 309+C 315+C 489 522-523d | 16013-16569/1-573 | | - | - | | | | | | Chin | Chin |
| 8 | Burman252 | Z | 185 189 193d 223 260 298 311 519 | 73 152 249d 263 315+C 489 | 16022-16569/1-574 | | | | | | | | 8821-9220=8860 9090 | Chin | Chin |
| 8 | Burman253* | M72a | 129 166d 213 214 223 342 526 | 73 263 315+C 489 | 16011-16569/1-574 | | | | | | | | | Chin | Chin |
| 8 | Burman254 | G | 213 223 246T 311 362 519 | 73 195 263 315+C 489 | 16009-16569/1-572 | | + | + | | | | | | Chin | Chin |
| 8 | Burman255 | G | 213 223 246T 311 362 519 | 73 195 263 315+C 489 | 16016-16569/1-574 | | + | + | | | | | | Chin | Chin |
| 8 | Burman256 | F1 | 086 111 304 519 | 73 152 234 249d 263 309+C 315+C 522-523d | 16017-16569/1-573 | | | | | - | | | | Chin | Chin |
| 8 | Burman257 | Z | 185 189 193d 223 260 298 311 362 519 | 73 152 249d 263 315+C 489 | 16033-16569/1-574 | | | | | | | | 8824-9220=8860 9090 | Chin | Chin |
| 8 | Burman258 | Z | 093 185 223 260 298 311 357 | 73 152 204 207 249d 263 309+C 315+C 489 522-523d | 16006-16569/1-324/423-565 | | | | | | | | 8815-9220=8860 9090 | Chin | Chin |
| 8 | Burman259 | M9a1b1 | 158 223 234 362 519 | 73 150 152 153 263 315+C 489 | 16004-16569/1-574 | | | | | | | | 4302-4616=4491 | Chin | Chin |
| 8 | Burman260 | F1 | 183C 189 294 304 311 519 | 73 143 146 249d 263 309+C 315+C | 16018-16569/1-316 | | | | | - | | | | Chin | Chin |
| 8 | Burman261 | M74 | 223 311 362 400 519 | 73 146 185 263 309+C 315+C 489 | 16007-16569/1-574 | | | | | | | | 4519-5317=4769 5054; 15012-15753=15043 15301 15326 | Chin | Chin |
| 8 | Burman262 | D | 223 362 | 73 263 309+C 315+C | 16011-16569/1-321 | | | | | | | | | Chin | Chin |
| 8 | Burman263 | Z | 185 189 193d 223 260 298 311 362 519 | 73 152 249d 263 315+C 489 | 16006-16569/1-574 | | | | | | | | 15622-16188=15784 | Chin | Chin |
| 8 | Burman264 | D | 223 362 | 73 263 309+C 315+C 489 522-523d | 16006-16569/1-573 | | - | - | | | | | | Chin | Chin |
| 8 | Burman265 | Z | 185 189 223 260 298 311 519 | 73 152 249d 263 315+C 489 | 16010-16569/1-574 | | | | | | | | 8827-9136=8860 9090 | Chin | Chin |
| 8 | Burman266 | F1 | 086 111 304 519 | 73 152 234 249d 263 309+C 315+C 522-523d | 16008-16569/1-573 | | | | | - | | | | Chin | Chin |
| 8 | Burman267 | D | 209 223 362 519 | 73 263 315+C 489 522-523d | 16007-16569/1-574 | | - | - | | | | | | Chin | Chin |
| 8 | Burman268 | Z | 185 189 193d 223 260 298 311 519 | 73 152 249d 263 315+C 489 | 16016-16569/1-574 | | | | | | | | 8831-9220=8860 9090 | Chin | Chin |
| 8 | Burman269 | F1 | 093 189 304 311 519 | 73 143 146 249d 263 309+C 315+C 522-523d | 16015-16569/1-573 | | | | | - | | | | Chin | Chin |
| 8 | Burman270 | M33a1 | 172 223 261 519 | 73 263 309+C 489 | 16017-16569/1-574 | | | | | | | | | Chin | Chin |
| 8 | Burman271 | G | 213 223 246T 311 362 519 | 73 195 263 315+C 489 | 16012-16569/1-574 | | | + | | | | | | Chin | Chin |
| 8 | Burman272 | M9a1b1 | 158 223 234 362 519 | 73 150 152 153 263 309+C 315+C 489 | 16022-16569/1-573 | | | | | | | | 4237-4691=4491 | Chin | Chin |
| 8 | Burman273 | G | 213 223 246T 311 362 519 | 73 195 263 315+C 489 | 16017-16569/1-574 | | | + | | | | | | Chin | Chin |
| 8 | Burman274 | A11 | 093 223 234 290 293C 319 519 | 73 152 235 263 315+C 522-523d | 16004-16569/1-574 | | | | | | | | | Chin | Chin |
| 8 | Burman275 | F1 | 086 111 304 519 | 73 152 234 249d 263 309+C 315+C 522-523d | 16004-16569/1-573 | | | | | - | | | | Chin | Chin |
| 8 | Burman276 | A | 223 290 319 362 | 73 151 152 235 263 309+C 315+C | 16021-16569/1-409 | | | | | | | | | Chin | Chin |
| 8 | Burman277 | F1 | 086 111 304 519 | 73 152 234 249d 263 522-523d | 16005-16569/1-575 | | | | | - | | | | Chin | Chin |
| 8 | Burman278 | Z | 185 189 193d 223 260 298 311 519 | 73 152 249d 263 315+C 489 | 16007-16569/1-574 | | | | | | | | 8814-9221=8860 9090 | Chin | Chin |
| 8 | Burman279 | F1 | 086 111 304 519 | 73 152 234 249d 263 309+C 315+C 522-523d | 16011-16569/1-573 | | | | | - | | | | Chin | Chin |
| 8 | Burman280 | D4 | 223 256 311 362 519 | 73 146 200 263 309+C 315+C 489 | 16007-16569/1-574 | | | - | | | | | | Chin | Chin |
| 8 | Burman281 | Z | 185 189 193d 223 260 298 311 519 | 73 152 249d 263 315+C 489 | 16005-16569/1-574 | | | | | | | | 8827-9217=8860 9090 | Chin | Chin |
| 8 | Burman282 | F1 | 086 111 304 519 | 73 152 234 249d 263 309+C 315+C 522-523d | 16016-16569/1-573 | | | | | - | | | | Chin | Chin |
| 8 | Burman283 | G | 213 223 246T 311 362 519 | 73 195 263 315+C 489 | 16004-16569/1-574 | | | + | | | | | | Chin | Chin |
| 8 | Burman284 | F1 | 086 111 304 519 | 73 152 234 249d 263 309+C 315+C 522-523d | 16006-16569/1-575 | | | | | - | | | | Chin | Chin |
| 8 | Burman285 | Z | 185 189 223 260 298 519 | 73 152 249d 263 315+C 489 | 16008-16569/1-574 | | | | | | | | 15622-15947=15784 | Chin | Chin |
| 8 | Burman286 | F1 | 189 304 311 519 | 73 146 249d 263 309+C 315+C 522-523d | 16016-16569/1-573 | | | | | - | | | | Chin | Chin |
| 8 | Burman287 | Z | 185 189 223 260 298 519 | 73 152 249d 263 315+C 489 | 16009-16569/1-574 | | | | | | | | 8827-9472=8860 9090 9215A | Chin | Chin |

| Code | Sample nam | Haplogroup | HVS-I (16000+) | HVS-II | Readable Region | 9bp | 4831*HhaI* | 5176*AluI* | 9820*HinfI* | 12406*HpaI* | 13262*AluI* | 14465*AccI* | Coding-Region Polymorphisms | Location | Nation |
|---|---|---|---|---|---|---|---|---|---|---|---|---|---|---|---|
| 8 | Burman288 | A5a | 187H 223 290 319 362 | 73 151 152 200 235 263 315+C 522-523d | 16015-16569/1-574 | | | | | | | | 417-1298=522-523d 663 735 750 | Chin | Chin |
| 8 | Burman289 | Z | 185 189 193d 223 260 298 519 | 73 152 249d 263 315+C 489 | 16010-16569/1-574 | | | | | | | | | Chin | Chin |
| 8 | Burman290 | F1 | 189 294 304 311 519 | 73 143 146 249d 263 309+C 315+C | 16008-16569/1-352 | | | | | - | | | | Chin | Chin |
| 8 | Burman291 | F1 | 183C 189 304 311 519 | 73 146 150 249d 263 309+C 315+C | 16004-16569/1-316 | | | | | - | | | 6367-6818=6620 | Chin | Chin |
| 8 | Burman292 | F1 | 086 111 304 519 | 73 152 234 249d 263 309+C 315+C 522-523d | 19004-16569/1-573 | | | | | - | | | | Chin | Chin |
| 8 | Burman293 | F1a1 | 129 162 172 304 327 519 | 73 249d 263 309+CC 315+C 522-523d 548 | 16007-16569/1-572 | | | | | - | | | | Chin | Chin |
| 8 | Burman294 | A11 | 093 223 234 290 293C 319 519 | 73 152 235 263 315+C 522-523d | 16004-16569/1-574 | | | | | | | | | Chin | Chin |
| 8 | Burman295 | F1 | 189 304 311 519 | 73 146 249d 263 309+C 315+C 522-523d | 16006-16569/1-573 | | | | | - | | | | Chin | Chin |
| 8 | Burman296 | M55 | 136 217 223 319 381 | 73 94 173 204 263 315+C 482 489 | 16007-16569/1-574 | | | | | | | | 9240-9731=9447 9540 | Chin | Chin |
| 8 | Burman297 | F1 | 189 294 304 311 519 | 73 143 146 249d 263 309+C 315+C 522-523d | 16022-16569/1-573 | | | | | - | | | | Chin | Chin |
| 8 | Burman298 | R21 | 172 182C 183C 189 356 399 519 | 73 152 263 309+C 315+C 522-523d | 16028-16569/1-575 | | | | | | | | | Chin | Chin |
| 8 | Burman299 | F1 | 189 284 304 362 519 | 73 146 152 249d 263 309+C 315+C 522-523d | 16015-16569/1-573 | | | | | - | | | | Chin | Chin |
| 8 | Burman300 | F1 | 189 304 311 519 | 73 146 152 249d 263 309+C 315+C 522-523d | 16008-16569/1-573 | | | | | - | | | | Chin | Chin |
| 8 | Burman301 | G | 192 223 295 519 | 73 150 195 263 315+C 489 | 16017-16569/1-568 | | | + | | | | | | Chin | Chin |
| 8 | Burman302 | M9a1b1 | 158 223 234 362 519 | 73 150 152 153 263 315+C 489 | 16017-16569/1-574 | | | | | | | | 4237-4942=4491 4769 | Chin | Chin |
| 8 | Burman303 | A5a | 187 223 290 319 362 | 73 151 152 200 235 263 315+C 522-523d | 16019-16569/1-574 | | | | | | | | | Chin | Chin |
| 8 | Burman304 | A11 | 093 223 234 290 293C 319 519 | 73 152 235 263 315+C 522-523d | 16018-16569/1-574 | | | | | | | | | Chin | Chin |
| 8 | Burman305 | G | 213 223 246T 311 362 519 | 73 195 263 315+C 489 | 16019-16569/1-574 | | | + | | | | | | Chin | Chin |
| 8 | Burman306 | U2 | 051 318 | 73 146 263 315+C 523+CA | 16017-16569/1-574 | | | | | | | | 12150-12472=12308 12372 | Chin | Chin |
| 8 | Burman307 | A11 | 093 223 234 290 293C 319 519 | 73 152 235 263 315+C 522-523d | 16019-16569/1-574 | | | | | | | | | Chin | Chin |
| 8 | Burman308 | Z | 185 189 193d 223 260 298 311 519 | 73 152 249d 263 315+C 489 | 16015-16569/1-574 | | | | | | | | 8820-9217=8860 9090 | Chin | Chin |
| 8 | Burman309 | F1 | 182C 183C 189 304 311 519 | 73 146 152 249d 263 309+CC? 315+C | 16032-16569/1-318 | | | | | - | | | | Chin | Chin |
| 8 | Burman310 | G | 213 223 246T 311 362 519 | 73 195 263 315+C 489 | 16015-16569/1-574 | | | + | | | | | | Chin | Chin |
| 8 | Burman311 | A | 223 246 290 319 362 | 73 151 152 200 235 263 315+C 522-523d | 16014-16569/1-574 | | | | | | | | | Chin | Chin |
| 8 | Burman312 | M9a1b1 | 158 223 234 362 519 | 73 150 152 153 263 315+C 489 | 16027-16569/1-574 | | | | | | | | 4224-4822=4491 4769 | Chin | Chin |
| 8 | Burman313 | F1 | 189 284 304 362 519 | 73 146 152 249d 263 309+C 315+C 522-523d | 16019-16569/1-574 | | | | | - | | | | Chin | Chin |
| 8 | Burman314 | M9a1b1 | 093 158 223 234 311 362 519 | 73 150 152 153 263 309+C 315+C 489 | 16017-16569/1-574 | | | | | | | | 4238-4997=4491 4769 | Chin | Chin |
| 8 | Burman315 | A11 | 093 223 234 290 293C 319 519 | 73 152 235 263 315+C 522-523d | 16018-16569/1-574 | | | | | | | | | Chin | Chin |
| 8 | Burman316 | G | 213 223 246T 311 362 519 | 73 195 263 315+C 489 | 16018-16569/1-574 | | | + | | | | | | Chin | Chin |
| 8 | Burman317 | Z | 185 189 193d 223 260 298 519 | 73 152 249d 263 315+C 489 | 16014-16569/1-574 | | | | | | | | 8815-9476=8860 9090 9215A | Chin | Chin |
| 8 | Burman318 | G | 213 223 246T 311 362 519 | 73 195 263 315+C 489 | 16018-16569/1-574 | | | + | | | | | | Chin | Chin |
| 8 | Burman319 | G | 213 223 246T 311 362 519 | 73 195 263 315+C 489 | 16017-16569/1-574 | | | + | | | | | | Chin | Chin |
| 8 | Burman320 | G | 213 223 246T 311 362 519 | 73 195 263 315+C 489 | 16018-16569/1-574 | | | + | | | | | | Chin | Chin |
| 8 | Burman321* | M33a1 | 172 223 261 519 | 73 263 315+C 489 | 16017-16569/1-574 | | | | | | | | | Chin | Chin |
| 8 | Burman322 | A5a | 187 223 290 319 362 | 73 151 152 200 235 263 315+C 522-523d | 16009-16569/1-574 | | | | | | | | | Chin | Chin |
| 8 | Burman323 | C | 051 189 223 298 327 519 | 73 249d 263 315+C 489 | 16009-16569/1-574 | | | | | | + | | | Chin | Chin |
| 8 | Burman324 | Z | 185 189 193d 223 260 298 311 362 519 | 73 152 249d 263 315+C 489 | 16017-16569/1-574 | | | | | | | | | Chin | Chin |
| 8 | Burman325 | F1 | 182C 183C 189 304 311 519 | 73 146 150 249d 263 309+C 315+C | 16017-16569/1-315 | | | | | - | | | | Chin | Chin |
| 8 | Burman326 | Z | 185 189 193d 223 260 298 311 519 | 73 152 249d 263 315+C 489 | 16014-16569/1-574 | | | | | | | | 15624-16187=15784 16185 | Chin | Chin |
| 8 | Burman327 | M9a1b1 | 158 223 234 362 519 | 73 150 152 153 263 315+C 489 | 16018-16569/1-574 | | | | | | | | 4232-4946=4491 4769 | Chin | Chin |
| 8 | Burman328 | A | 223 290 319 362 | 73 151 152 200 235 263 315+C 522-523d | 16018-16569/1-574 | | | | | | | | | Chin | Chin |
| 8 | Burman329 | M72a | 129 166d 213 214 223 342 526 | 73 263 315+C 489 | 16014-16569/1-574 | | | | | | | | | Chin | Chin |
| 8 | Burman330 | M33b | 223 259 264 324 362 519 | 73 185 204 263 309+C 315+C 489 | 16017-16569/1-574 | | | | | | | | 3200-3570=3221 | Chin | Chin |
| 8 | Burman331 | U2 | 051 318 | 73 146 263 315+C 523+CA | 16018-16569/1-574 | | | | | | | | 12059-12422=12106 12308 12372 | Chin | Chin |
| 8 | Burman332 | G | 213 223 246T 311 362 519 | 73 195 263 315+C 489 | 16019-16569/1-574 | | | + | | | | | | Chin | Chin |
| 8 | Burman333 | A11 | 093 223 234 290 293C 319 519 | 73 152 235 263 315+C 522-523d | 16014-16569/1-574 | | | | | | | | | Chin | Chin |
| 8 | Burman334 | A | 223 290 319 362 | 73 151 152 200 235 263 315+C 522-523d | 16017-16569/1-574 | | | | | | | | | Chin | Chin |
| 8 | Burman335 | C | 223 298 327 519 | 73 146 152 249d 263 309+C 315+C | 16021-16569/1-347 | | | | | | + | | | Chin | Chin |
| 8 | Burman336 | A11 | 223 234 290 293C 319 519 | 73 152 235 263 309+CC 315+C 522-523d | 16017-16569/1-574 | | | | | | | | | Chin | Chin |
| 8 | Burman337 | Z | 185 189 193d 223 260 298 519 | 73 152 249d 263 315+C 489 | 16017-16569/1-574 | | | | | | | | 15626-16122=15784 | Chin | Chin |
| 8 | Burman338 | M51 | 223 278 519 | 73 150 152 263 309+C 315+C 489 522-523d | 16024-16569/1-574 | | | | | | | | 14081-14582=14110 14161 14356 14527 | Chin | Chin |
| 8 | Burman339 | F1 | 189 304 311 519 | 73 146 249d 263 309+C 315+C 522-523d | 16019-16569/1-573 | | | | | - | | | | Chin | Chin |
| 8 | Burman340 | M9a1b1 | 158 223 234 362 519 | 73 150 152 153 263 315+C 489 | 16007-16569/1-574 | | | | | | | | 4237-4961=4491 4769 | Chin | Chin |
| 8 | Burman341 | A | 223 290 319 362 | 73 151 152 200 235 263 309+C 315+C 522-523d | 16008-16569/1-574 | | | | | | | | | Chin | Chin |
| 8 | Burman342 | M84 | 223 258d 272 519 | 73 185 263 315+C 489 | 16017-16569/1-574 | | | | | | | | 1538-1892=1719 1809 | Chin | Chin |
| 8 | Burman343 | A | 223 290 311 319 362 | 73 151 152 200 235 263 315+C 522-523d | 16017-16569/1-574 | | | | | | | | | Chin | Chin |
| 8 | Burman344 | F1a | 129 172 304 519 | 73 249d 263 309+C 315+C 522-523d | 16032-16569/1-574 | | | | | - | | | | Chin | Chin |
| 8 | Burman345 | G | 213 223 246T 311 362 519 | 73 195 263 315+C 489 | 16019-16569/1-574 | | | + | | | | | | Chin | Chin |
| 8 | Burman346* | M55 | 136 217 223 319 381 | 73 94 173 204 263 315+C 482 489 522-523d | 16020-16569/1-574 | | | | | | | | | Chin | Chin |
| 8 | Burman347 | A11 | 093 223 234 290 293C 319 519 | 73 152 235 263 315+C 522-523d | 16024-16569/1-348 | | | | | | | | | Chin | Chin |
| 8 | Burman348 | M9a1a2 | 145 223 234 316 | 73 153 263 309+CC 315+C 489 513 | 16016-16569/1-574 | | | | | | | | 4238-4956=4491 4769 | Chin | Chin |
| 8 | Burman349 | M74 | 223 311 362 400 519 | 73 146 185 263 309+C 315+C 489 | 16018-16569/1-574 | | | + | | | | | | Chin | Chin |
| 8 | Burman350 | M72a | 129 166d 214 223 526 | 63 73 263 315+C 489 | 16032-16569/1-574 | | | | | | | | 15416-16024=15497 15644 15820 | Chin | Chin |
| 8 | Burman351 | G | 129 213 223 246T 311 362 519 | 73 195 263 315+C 489 | 16017-16569/1-574 | | | + | | | | | | Chin | Chin |
| 8 | Burman352 | G | 192 223 295 519 | 73 150 195 263 315+C 489 573+CC | 16023-16569/1-574 | | | + | | | | | | Chin | Chin |

| Code | Sample nam | Haplogroup | HVS-I (16000+) | HVS-II | Readable Region | 9bp | 4831*HhaI* | 5176*AluI* | 9820*HinfI* | 12406*HpaI* | 13262*AluI* | 14465*AccI* | Coding-Region Polymorphisms | Location | Nation |
|---|---|---|---|---|---|---|---|---|---|---|---|---|---|---|---|
| 8 | Burman353 | F1 | 189 294 304 311 519 | 73 143 146 249d 263 309+C 315+C 522-523d | 16026-16569/1-573 | | | + | | - | | | | Chin | Chin |
| 8 | Burman354 | M49 | 223 234 390 519 | 73 152 249d 263 279 309+CC 315+C 489 | 16017-16569/1-574 | | | | | | | | 3668-4236=3780 | Chin | Chin |
| 8 | Burman355 | F1 | 086 111 304 519 | 73 152 234 249d 263 309+C 315+C 522-523d | 16018-16569/1-573 | | | | | - | | | | Chin | Chin |
| 8 | Burman356 | D | 126 223 362 | 73 263 309+C 315+C 489 522-523d | 16021-16569/1-574 | | | - | | | | | | Chin | Chin |
| 8 | Burman357 | D | 126 223 362 | 73 263 309+C 315+C 489 522-523d | 16018-16569/1-574 | | | - | | | | | | Chin | Chin |
| 8 | Burman358 | D | 092 223 311 362 | 73 94 189 207 214 263 315+C 489 | 16018-16569/1-574 | | | | | | | | 4521-5531=4769 4883 5178A | Chin | Chin |
| 8 | Burman359 | F2a | 092A 182C 183C 189 291 304 311 | 73 249d 263 315+C | 16025-16569/1-316 | | | | | + | | | | Chin | Chin |
| 8 | Burman360 | A11 | 093 223 234 290 293C 319 519 | 73 152 235 263 315+C 522-523d | 16032-16569/1-574 | | | | | | | | | Chin | Chin |
| 8 | Burman361 | A11 | 093 223 234 290 293C 319 519 | 73 152 235 263 315+C 522-523d | 16017-16569/1-574 | | | | | | | | | Chin | Chin |
| 8 | Burman362 | A | 223 290 319 362 | 73 151 152 200 235 263 315+C 522-523d | 16017-16569/1-574 | | | | | | | | | Chin | Chin |
| 8 | Burman363 | Z | 114 185 189 193d 223 260 298 519 | 73 152 249d 263 315+C 489 | 16017-16569/1-574 | | | | | | | | 15623-15908=15784 | Chin | Chin |
| 8 | Burman364 | G | 213 223 246T 311 362 519 | 73 195 263 315+C 489 | 16017-16569/1-574 | | | + | | | | | | Chin | Chin |
| 8 | Burman365 | G | 213 223 246T 311 362 519 | 73 195 263 315+C 489 | 16010-16569/1-574 | | | + | | | | | | Chin | Chin |
| 8 | Burman366 | M84 | 093 223 258d 272 519 | 73 185 263 315+C 489 | 16032-16569/1-574 | | | | | | | | 1532-1882=1719 1809 | Chin | Chin |
| 8 | Burman367 | F1 | 183C 189 304 311 519 | 73 146 249d 263 309+C 315+C 522-523d | 16018-16569/1-574 | | | | | - | | | | Chin | Chin |
| 8 | Burman368 | F1 | 183C 189 304 311 519 | 73 146 150 249d 263 309+C 315+C 522-523d | 16017-16569/1-574 | | | | | - | | | | Chin | Chin |
| 8 | Burman369 | D | 223 362 | 73 263 309+C 315+C 489 522-523d 573+C | 16017-16569/1-574 | | | - | | | | | | Chin | Chin |
| 8 | Burman370 | A | 223 290 319 362 | 73 151 152 200 235 263 315+C 522-523d | 16017-16569/1-574 | | | | | | | | | Chin | Chin |
| 8 | Burman371 | A | 223 246 290 319 362 | 73 151 152 200 235 263 315+C 522-523d | 16018-16569/1-574 | | | | | | | | | Chin | Chin |
| 8 | Burman372 | G | 213 223 246T 311 362 519 | 73 195 263 315+C 489 | 16018-16569/1-574 | | | + | | | | | | Chin | Chin |
| 8 | Burman373 | G | 213 223 246T 311 362 519 | 73 195 263 315+C 489 | 16017-16569/1-574 | | | + | | | | | | Chin | Chin |
| 8 | Burman374 | F1a1 | 129 162 172 304 519 | 73 249d 263 315+C 522-523d 548 | 16033-16569/1-574 | | | | | - | | | | Chin | Chin |
| 8 | Burman375 | R31 | 218 289 304 526 | 73 152 183 184 185 204 263 315+CC | 16017-16569/1-574 | | | | | | | | 1502-1890=1531 | Chin | Chin |
| 8 | Burman376* | M72a | 129 166d 214 223 526 | 63 73 263 315+C 489 | 16017-16569/1-574 | | | | | | | | | Chin | Chin |
| 8 | Burman377 | M72a | 129 166d 214 223 526 | 63 64 73 263 315+C 489 | 16001-16569/1-574 | | | | | | | | 15409-16024=15497 15644 15820 | Chin | Chin |
| 8 | Burman378 | Z | 185 189 223 260 298 519 | 73 152 249d 263 315+C 489 | 16018-16569/1-574 | | | | | | | | 8811-9506=8860 9090 9215A | Chin | Chin |
| 8 | Burman379 | C | 223 298 327 519 | 73 146 153 249d 263 309+C 315+C 489 | 16013-16569/1-574 | | | | | | + | | | Chin | Chin |
| 8 | Burman380 | Z | 185 189 223 260 298 519 | 73 152 249d 263 315+C 489 | 16015-16569/1-574 | | | | | | | | 8822-9506=8860 9090 9215A | Chin | Chin |
| 8 | Burman381 | D | 223 362 | 73 263 309+C 315+C 489 522-523d 573+C | 16017-16569/1-574 | | | - | | | | | | Chin | Chin |
| 8 | Burman382 | A | 223 290 319 362 | 73 151 152 200 235 263 315+C 522-523d | 16017-16569/1-574 | | | | | | | | | Chin | Chin |
| 8 | Burman383 | F1 | 189 304 311 519 | 73 146 249d 263 309+C 315+C | 16017-16569/1-316 | | | | | - | | | | Chin | Chin |
| 8 | Burman384 | D | 223 362 | 73 263 309+C 315+C 489 522-523d 573+C | 16017-16569/1-574 | | | - | | | | | | Chin | Chin |
| 8 | Burman385 | M9a1b1 | 158 223 234 362 519 | 73 150 152 153 263 315+C 489 | 16009-16569/1-480 | | | | | | | | 4237-4961=4491 4769 | Chin | Chin |
| 8 | Burman386* | M55 | 136 189 217 223 319 381 | 73 94 173 204 263 315+C 482 489 522-523d | 16001-16569/1-574 | | | | | | | | 9230-9728=9477 9540 | Chin | Chin |
| 8 | Burman387 | F1a1 | 129 162 172 304 327 519 | 73 249d 263 309+CC 315+C | 16017-16569/1-316 | | | | | - | | | | Chin | Chin |
| 8 | Burman388 | A | 223 290 319 362 | 73 151 152 200 235 263 315+C 456 522-523d | 16011-16569/1-574 | | | | | | | | | Chin | Chin |
| 8 | Burman389 | G | 192 223 295 519 | 73 150 195 263 315+C 489 573+CC | 16024-16569/1-574 | | | + | | | | | | Chin | Chin |
| 8 | Burman390 | Z | 185 189 193d 223 260 298 519 | 73 152 249d 263 315+C 489 | 16019-16569/1-574 | | | | | | | | 15614-16382=15784 | Chin | Chin |
| 8 | Burman391 | M33b | 223 259 264 324 362 519 | 73 185 204 263 309+C 315+C 489 | 16014-16569/1-574 | | | | | | | | 3203-3675=3221 | Chin | Chin |
| 8 | Burman392 | F1 | 086 111 304 519 | 73 152 234 249d 263 309+C 315+C | 16033-16569/1-316 | | | | | - | | | | Chin | Chin |
| 8 | Burman393 | M55 | 136 217 223 319 381 | 73 94 173 204 263 315+C 482 489 522-523d | 16017-16569/1-574 | | | | | | | | 9228-9592=9447 9540 | Chin | Chin |
| 8 | Burman394 | A | 223 290 319 362 | 73 151 152 200 235 263 315+C 522-523d | 16017-16569/1-574 | | | | | | | | | Chin | Chin |
| 8 | Burman395 | M33b | 051 093 223 266 324 355 362 391 519 | 73 146 263 309+C 315+C 489 | 16017-16569/1-574 | | | | | | | | 3206-3342=3221 | Chin | Chin |
| 8 | Burman396 | G | 213 223 246T 311 362 519 | 73 195 263 315+C 489 | 16017-16569/1-574 | | | + | | | | | | Chin | Chin |
| 8 | Burman397 | Z | 185 189 193d 223 260 298 311 362 519 | 73 152 249d 263 315+C 489 | 16022-16569/1-574 | | | | | | | | 15626-15945=15784 | Chin | Chin |
| 8 | Burman398 | M55 | 136 217 223 319 381 | 73 94 173 204 263 315+C 482 489 522-523d | 16024-16569/1-574 | | | | | | | | 9241-9765=9447 9540 | Chin | Chin |
| 8 | Burman399* | M51 | 223 278 519 | 73 150 152 263 309+C 315+C 489 522-523d | 16023-16569/1-574 | | | | | | | | | Chin | Chin |
| 8 | Burman400 | A | 223 290 319 362 | 73 151 152 200 235 263 315+C 456 522-523d | 16030-16569/1-574 | | | | | | | | | Chin | Chin |
| 8 | Burman401 | F1 | 189 304 311 519 | 73 146 249d 263 309+C 315+C 522-523d | 16018-16569/1-574 | | | | | - | | | | Chin | Chin |
| 8 | Burman402 | F1c | 111 129 220 304 519 | 73 152 234 249d 263 309+CC 315+C | 16018-16569/1-316 | | | | | - | | | | Chin | Chin |
| 8 | Burman403 | A | 223 290 319 362 | 73 151 152 200 235 263 315+C 523d | 16019-16569/1-574 | | | | | | | | | Chin | Chin |
| 8 | Burman404 | M33b | 051 093 223 266 324 355 362 391 519 | 73 146 199 263 309+C 315+C | 16017-16569/1-316 | | | | | | | | 3205-3666=3221 | Chin | Chin |
| 8 | Burman405 | M84 | 093 223 258d 272 519 | 73 185 263 315+C 489 | 16017-16569/1-574 | | | | | | | | 1518-2018=1719 1809 | Chin | Chin |
| 8 | Burman406 | F1 | 086 111 304 519 | 73 152 234 249d 263 309+C 315+C 522-523d | 16017-16569/1-573 | | | | | - | | | | Chin | Chin |
| 8 | Burman407* | M33b | 051 093 223 266 324 355 362 391 519 | 73 146 263 309+C 315+C 489 | 16017-16569/1-574 | | | | | | | | | Chin | Chin |
| 8 | Burman408 | M49 | 223 234 390 519 | 73 152 249d 263 279 309+CC 315+C | 16017-16569/1-316 | | | | | | | | 3676-4400=3780 | Chin | Chin |
| 8 | Burman409 | Z | 185 223 249 260 298 | 73 143 152 249d 263 315+C 489 573+CCCCC | 16032-16569/1-574 | | | | | | | | 8818-9525=8860 9090 9477 | Chin | Chin |
| 8 | Burman410 | F1 | 189 304 311 519 | 73 146 249d 263 309+C 315+C 522-523d | 16023-16569/1-573 | | | | | - | | | | Chin | Chin |
| 8 | Burman411 | N21 | 182 193 223 260 519 | 73 150 195 263 309+C 315+C 337d | 16015-16569/1-574 | | | | | | | | 13101-13723=13392 13437 | Chin | Chin |
| 8 | Burman412 | N21 | 182 193 223 260 519 | 73 150 195 263 309+C 315+C 337d | 16010-16569/1-574 | | | | | | | | 13097-13764=13437 | Chin | Chin |
| 8 | Burman413 | N21 | 182 193 223 260 519 | 73 150 195 263 309+C 315+C 337d | 16016-16569/1-574 | | | | | | | | 13309-13504=13437 | Chin | Chin |
| 9 | Burman637 | F1c | 111 129 304 519 | 73 152 234 249d 263 315+C 522-523d | 16013-16569/1-574 | | | | | - | | | | Magway | Chin |
| 9 | Burman638 | F1c | 111 129 304 519 | 73 152 234 249d 263 315+C 522-523d | 16013-16569/1-574 | | | | | - | | | | Magway | Chin |
| 9 | Burman639 | D | 092 223 311 362 | 73 94 262 263 315+C 489 | 16018-16569/1-512 | | | | | | | | 4520-5107=4769 4883; 15011-15762=15043 15119 15301 15326 15663 | Magway | Chin |
| 9 | Burman640* | M84 | 223 258d 272 519 | 73 185 263 315+C 489 | 16012-16569/1-574 | | | | | | | | | Magway | Chin |

| Code | Sample nam | Haplogroup | HVS-I (16000+) | HVS-II | Readable Region | 9bp | 4831*HhaI* | 5176*AluI* | 9820*HinfI* | 12406*HpaI* | 13262*AluI* | 14465*AccI* | Coding-Region Polymorphisms | Location | Nation |
|---|---|---|---|---|---|---|---|---|---|---|---|---|---|---|---|
| 9 | Burman641 | M84 | 223 258d 272 519 | 73 185 263 315+C 489 | 16014-16569/1-574 | | | | | | | | 1516-1881=1719 1809 | Magway | Chin |
| 9 | Burman642 | F1a1a | 108 129 162 172 304 368 519 | 73 249d 263 309+C 315+C 522-523d | 16013-16569/1-574 | | | | | - | | | | Magway | Chin |
| 9 | Burman643 | D5a2 | 092 164 172 182C 183C 189 223 266 362 519 | 73 150 263 309+C 315+C | 16013-16569/1-315 | | | | | | | | | Magway | Chin |
| 9 | Burman644 | M55 | 136 217 223 319 381 | 73 94 173 204 263 315+C 482 489 | 16013-16569/1-574 | | | | | | | | 9228-9673=9447 9540 | Magway | Chin |
| 9 | Burman645* | M84 | 193 223 258d 272 519 | 73 185 263 315+C 489 | 16013-16569/1-564 | | | | | | | | 1530-1860=1719 1809 | Magway | Chin |
| 9 | Burman646* | R22a | 169 224 249 265C 288 291 304 519 | 73 152 199 263 309+C 315+C 329 | 16012-16569/1-574 | | | | | | | | | Magway | Chin |
| 9 | Burman647 | D | 092 223 311 362 | 73 94 262 263 315+C 489 | 16012-16569/1-574 | | | | | | | | 4519-5223=4769 4883 5178A; 15011-15782=15043 15301 15326 | Magway | Chin |
| 9 | Burman648 | M9a1a2 | 145 223 234 316 519 | 73 153 263 309+C 315+C 489 513 | 16017-16569/1-523 | | | | | | | | 4229-4899=4491 4769 | Magway | Chin |
| 9 | Burman649 | M84 | 093 223 258d 272 519 | 73 185 188 195 263 315+C | 16009-16569/1-364 | | | | | | | | 1532-1871=1719 1809 | Magway | Chin |
| 10 | Burman414 | M38 | 189 223 242A 249 519 | 73 153 246 263 309+C 315+C | 16012-16569/1-316 | | | | | | | | 15011-15650=15043 15301 15326 15314 15326 15487 | Sagaing | Naga |
| 10 | Burman415 | G2a1 | 093 126 223 227 278 362 | 73 263 309+C 315+C 489 | 16061-16569/1-574 | | + | | | | | | | Sagaing | Naga |
| 10 | Burman416* | M24 | 223 311 519 | 73 146 152 195 204 263 315+C 489 | 16042-16569/1-574 | | | | | | | | 15012-15749=15043 15301 15326 15601 | Sagaing | Naga |
| 10 | Burman417 | M33b | 223 259 324 362 389 519 | 73 263 309+C 315+C 489 | 16017-16569/1-574 | | | | | | | | 3200-3663=3221 | Sagaing | Naga |
| 10 | Burman418 | M33b | 223 259 324 362 389 519 | 73 263 309+C 315+C 489 | 16001-16569/1-574 | | | | | | | | 3203-3676=3221 | Sagaing | Naga |
| 10 | Burman419* | M55 | 217 223 319 381 519 | 73 94 173 204 263 315+C 482 489 | 16043-16569/1-574 | | | | | | | | 9241-9570=9447 9540 | Sagaing | Naga |
| 10 | Burman420 | M24 | 223 311 519 | 73 146 152 195 204 263 315+C 489 | 16043-16569/1-574 | | | | | | | | 15013-15800=15043 15301 15326 15601 | Sagaing | Naga |
| 10 | Burman421 | M24 | 223 311 519 | 73 146 152 195 204 263 6315+C 489 | 16043-16569/1-574 | | | | | | | | 15011-15890=15043 15301 15326 15601 | Sagaing | Naga |
| 10 | Burman422 | M33b | 223 259 324 362 389 519 | 73 263 309+C 315+C 489 | 16021-16569/1-574 | | | | | | | | 3198-3571=3221 | Sagaing | Naga |
| 10 | Burman423 | M33b | 223 259 324 362 389 519 | 73 263 309+C 315+C 489 | 16014-16569/1-328 | | | | | | | | 3198-3678=3221 | Sagaing | Naga |
| 10 | Burman424 | M11 | 104 223 | 73 198 200 215 263 309+C 315+C | 16023-16569/1-317 | | | | | | | | 7375-8231=7642 8108 13074 | Sagaing | Naga |
| 10 | Burman425 | F1c | 111 129 304 519 | 73 152 228 234 249d 263 315+C 522-523d | 16032-16569/1-574 | | | | | - | | | | Sagaing | Naga |
| 10 | Burman426 | M33b | 223 259 324 362 389 519 | 73 263 309+C 315+C 489 | 16071-16569/1-491 | | | | | | | | 3201-3589=3221 | Sagaing | Naga |
| 10 | Burman427 | G2a1 | 093 126 223 227 278 362 | 73 263 309+C 315+C 489 | 16043-16569/1-574 | | + | | | | | | | Sagaing | Naga |
| 10 | Burman428 | M24 | 223 311 519 | 73 146 152 195 204 263 315+C 489 | 16061-16569/1-574 | | | | | | | | 15010-15780=15043 15301 15326 15601 | Sagaing | Naga |
| 10 | Burman429 | M24 | 223 311 519 | 73 146 152 195 204 263 315+C 489 | 16017-16569/1-574 | | | | | | | | 15012-15999=15043 15301 15326 15601 | Sagaing | Naga |
| 10 | Burman430 | D | 223 311 362 519 | 73 263 315+C 489 | 16043-16569/1-574 | | | | | | | | 4519-4980=4769 4883; 15012-15735=15043 15301 15326 | Sagaing | Naga |
| 10 | Burman431 | G | 223 294 362 | 73 183 263 309+C 315+C 489 | 16035-16569/1-574 | | | + | | | | | | Sagaing | Naga |
| 10 | Burman432 | M55 | 217 223 319 381 519 | 73 94 173 204 263 315+C 482 489 | 16067-16569/1-574 | | | | | | | | 9223-9660=9447 9540 | Sagaing | Naga |
| 10 | Burman433 | M24 | 223 311 519 | 73 146 152 195 204 263 315+C 489 | 16065-16569/1-574 | | | | | | | | 15011-15800=15043 15301 15326 15601 | Sagaing | Naga |
| 10 | Burman434 | M11 | 104 223 | 73 198 200 215 263 309+C 315+C 318 326 489 | 16017-16569/1-493 | | | | | | | | 940-1557=1095 1438 | Sagaing | Naga |
| 10 | Burman435* | M33b | 223 259 324 362 389 519 | 73 263 309+C 315+C 489 | 16043-16569/1-574 | | | | | | | | | Sagaing | Naga |
| 10 | Burman436 | M24 | 223 311 519 | 73 146 152 195 204 263 315+C 489 | 16012-16569/1-574 | | | | | | | | 15412-15965=15601 | Sagaing | Naga |
| 10 | Burman437 | F1c | 111 129 304 519 | 73 152 234 249d 263 315+C 522-523d | 16014-16569/1-574 | | | | | - | | | | Sagaing | Naga |
| 10 | Burman438 | G | 223 294 362 | 73 183 263 309+C 315+C 489 | 16012-16569/1-574 | | + | + | | | | | | Sagaing | Naga |
| 10 | Burman439 | G | 223 294 362 | 73 183 263 309+C 315+C 489 | 16021-16569/1-548 | | + | + | | | | | | Sagaing | Naga |
| 10 | Burman440 | G | 223 294 362 | 73 183 263 309+C 315+C 489 | 16012-16569/1-490 | | | | | | | | 15015-15534=15043 15301 15326 | Sagaing | Naga |
| 10 | Burman441 | M24 | 223 311 519 | 73 146 152 195 204 263 315+C 489 | 16043-16569/1-574 | | | | | | | | 15011-15928=15043 15301 15326 15601 | Sagaing | Naga |
| 10 | Burman442 | G | 223 294 362 | 73 183 263 309+C 315+C 489 | 16043-16569/1-574 | | + | + | | | | | | Sagaing | Naga |
| 10 | Burman443 | M33b | 223 259 324 362 389 519 | 73 263 309+C 315+C 489 | 16043-16569/1-555 | | | | | | | | 3203-3447=3221 | Sagaing | Naga |
| 10 | Burman444 | M33b | 223 259 324 362 389 519 | 73 263 309+C 315+C 489 | 16043-16569/1-574 | | | | | | | | 3198-3678=3221 | Sagaing | Naga |
| 10 | Burman445 | M24 | 223 311 519 | 73 146 152 195 204 263 315+C 489 | 16021-16569/1-574 | | | | | | | | 15011-15725=15043 15301 15326 15601 | Sagaing | Naga |
| 11 | Burman446* | HV | 129 242 356 | 263 309+CC 315+C | 16019-16569/1-316 | | | | | | | | | Sagaing | Naga |
| 11 | Burman447 | M10 | 066 223 311 | 73 263 315+C 489 | 16011-16569/1-573 | | | | | | - | | 15017-15509=15040 15043 15071 15218 15301 15326 | Sagaing | Naga |
| 11 | Burman448 | M10 | 066 223 311 | 73 263 315+C 489 | 16043-16569/1-550 | | | | | | | | 15002-15677=15040 15043 15071 15218 15301 15326 | Sagaing | Naga |
| 11 | Burman449 | M9a1b1 | 158 223 234 519 | 73 150 152 153 263 315+C 489 | 16013-16569/1-574 | | | | | | | | 4237-4912=4491 4769 | Sagaing | Naga |
| 11 | Burman450 | M10 | 066 223 311 | 73 263 315+C 489 | 16032-16569/1-506 | | | | | | | | 15011-15822=15040 15043 15071 15218 15301 15326 | Sagaing | Naga |
| 11 | Burman451 | M10 | 066 223 311 | 73 263 315+C 489 | 16043-16569/1-509 | | | | | | | | 15011-15686=15040 15043 15071 15218 15301 15326 | Sagaing | Naga |
| 11 | Burman452 | F1 | 183C 189 304 519 | 73 249d 263 309+C 315+C | 16043-16569/1-316 | | | | | - | | | | Sagaing | Naga |
| 11 | Burman453 | M9a | 223 234 362 519 | 73 150 152 153 263 315+C | 16016-16569/1-434 | | | | | | | | 4238-4961=4491 4769 | Sagaing | Naga |
| 11 | Burman454 | D5a2 | 092 164 182C 183C 189 223 266 362 | 73 146 150 263 315+C 489 522-523d | 16043-16569/1-574 | | - | - | | | | | | Sagaing | Naga |
| 11 | Burman455 | C | 223 298 327 357 519 | 47 73 214 249d 263 309+CC 315+C | 16043-16569/1-316 | | | | | | + | | | Sagaing | Naga |
| 11 | Burman456 | C | 051 223 298 519 | 73 249d 263 315+C 489 | 16018-16569/1-574 | | | | | | + | | | Sagaing | Naga |
| 11 | Burman457 | G | 223 294 362 | 73 151 183 263 309+C 315+C 489 | 16018-16569/1-574 | + | | + | | | | | | Sagaing | Naga |
| 11 | Burman458 | C | 093 129 223 298 327 519 | 73 249d 263 315+C 489 | 16033-16569/1-574 | | | | | | + | | | Sagaing | Naga |
| 11 | Burman459* | M54 | 188 192 223 304 311 519 | 73 146 263 315+C 489 | 16043-16569/1-574 | | | | | | | | | Sagaing | Naga |
| 11 | Burman460 | C | 093 129 223 298 327 519 | 73 249d 263 315+C 489 | 16020-16569/1-574 | | | | | | + | | | Sagaing | Naga |
| 11 | Burman461 | F1b | 183C 189 232A 249 304 311 519 | 73 146 204 249d 263 309+C 315+C 522-523d | 16043-16569/1-574 | | | | | - | | | | Sagaing | Naga |
| 11 | Burman462 | F1b | 183C 189 232A 249 304 311 519 | 73 146 204 249d 263 309+C 315+C 522-523d | 16043-16569/1-574 | | | | | - | | | | Sagaing | Naga |
| 11 | Burman463 | M10 | 066 223 311 | 73 263 315+C 489 573+CCC | 16013-16569/1-572 | | | | | | | | 15012-15648=15040 15043 15071 15218 15301 15326 | Sagaing | Naga |
| 11 | Burman464 | C | 086 223 298 327 519 | 73 249d 263 309+C 315+C 489 | 16009-16569/1-542 | | | | | | + | | | Sagaing | Naga |
| 11 | Burman465 | C | 086 223 298 327 519 | 73 249d 263 309+C 315+C 489 | 16043-16569/1-574 | | | | | | + | | | Sagaing | Naga |
| 11 | Burman466 | F1b | 183C 189 232A 249 304 311 519 | 73 146 204 249d 263 309+C 315+C 522-523d | 16008-16569/1-574 | | | | | - | | | | Sagaing | Naga |
| 11 | Burman467 | C | 086 223 298 327 519 | 73 249d 263 309+C 315+C 489 | 16013-16569/1-574 | | | | | + | | | 13085-13440=13263 | Sagaing | Naga |
| 11 | Burman468 | M49 | 223 225 234 390 519 | 73 199 249d 263 309+CC 315+C 489 | 16043-16569/1-574 | | | | | | | | 3677-4310=3780 | Sagaing | Naga |
| 11 | Burman469 | M10 | 066 223 311 | 73 263 315+C 489 | 16012-16569/1-490 | | | | | | | | 15011-15698=15040 15043 15071 15218 15301 15326 | Sagaing | Naga |

| Code | Sample nam | Haplogroup | HVS-I (16000+) | HVS-II | Readable Region | 9bp | 4831*Hha*I | 5176*Alu*I | 9820*Hinf*I | 12406*Hpa*I | 13262*Alu*I | 14465*Acc*I | Coding-Region Polymorphisms | Location | Nation |
|---|---|---|---|---|---|---|---|---|---|---|---|---|---|---|---|
| 11 | Burman470 | B5a | 140 183C 189 261 266A 519 | 73 152 189 210 263 309+C 315+C 522-523d | 16061-16569/1-574 | + | | | | | | | | Sagaing | Naga |
| 11 | Burman471 | M24 | 223 311 519 | 73 146 152 195 204 263 315+C 489 | 16043-16569/1-574 | | | | | | | | 15012-15900=15043 15301 15326 15601 | Sagaing | Naga |
| 11 | Burman472 | M9a1b1 | 158 223 234 362 519 | 73 150 152 263 315+C 489 | 16075-16569/1-574 | | | | | | | | 4237-4950=4491 4769 | Sagaing | Naga |
| 11 | Burman473 | F2a | 092A 093 124 291 304 | 73 249d 263 309+C 315+C 522-523d | 16007-16569/1-574 | | | | | | | | 6364-6803=6392 6782; 15011-15783=15326 | Sagaing | Naga |
| 11 | Burman474 | D5a2 | 092 164 182C 183C 189 223 266 362 | 73 146 150 263 315+C 489 522-523d | 16043-16569/1-574 | | - | - | | | | | | Sagaing | Naga |
| 11 | Burman475 | G | 223 294 362 | 73 151 183 263 309+C 315+C 489 | 16043-16569/1-574 | + | | + | | | | | | Sagaing | Naga |
| 12 | Burman476 | B5a | 140 183C 189 261 266A 519 | 73 152 210 263 309+C 315+C 522-523d | 16043-19569/1-574 | + | | | | | | | | Sagaing | Naga |
| 12 | Burman477 | M9a1b1 | 158 223 234 362 519 | 73 150 152 263 309+C 315+C 489 | 16063-16569/1-574 | | | | | | | | 4257-4961=4491 4769 | Sagaing | Naga |
| 12 | Burman478 | G | 124 223 244C 246T 295 356 362 519 | 73 200 263 315+CC 468 489 521 | 16027-16569/1-574 | | + | + | | | | | | Sagaing | Naga |
| 12 | Burman479 | D4 | 223 256 311 362 519 | 73 200 263 309+C 315+C 489 | 16012-16569/1-574 | | | | | | | | | Sagaing | Naga |
| 12 | Burman480 | M13b | 145 168 188 223 257 311 519 | 73 152 263 315+C 489 513 | 16043-16569/1-574 | | | | | | | | 10186-10735=10373 10398 10400 10411 | Sagaing | Naga |
| 12 | Burman481 | M13b | 145 168 188 223 257 311 519 | 73 152 263 315+C 489 513 | 16043-16569/1-574 | | | | | | | | 10185-10669=10373 10398 10400 10411 | Sagaing | Naga |
| 12 | Burman482 | M83 | 223 320 | 73 146 207 263 315+C 356+C 489 | 16043-16569/1-490 | | | | | | | | 7908-8442=8143 8271 8307 | Sagaing | Naga |
| 12 | Burman483 | B5a | 140 183C 189 261 266A 519 | 73 152 210 263 309+C 315+C 522-523d | 16017-16569/1-573 | + | | | | | | | | Sagaing | Naga |
| 12 | Burman484* | M83 | 129 223 320 | 73 146 207 263 309+C 315+C 356+C 489 | 16017-16569/1-490 | | | | | | | | | Sagaing | Naga |
| 12 | Burman485* | M51 | 223 263 278 | 73 150 152 263 309+C 315+C 489 522-523d | 16067-16569/1-574 | | | | | | | | | Sagaing | Naga |
| 12 | Burman486 | M9a | 129 223 234 362 519 | 73 150 152 153 263 315+C 489 | 16017-16569/1-544 | | | | | | | | 4237-4915=4491 4769 | Sagaing | Naga |
| 12 | Burman487 | F1c | 111 129 183C 189 304 519 | 73 152 234 249d 263 315+C 522-523d | 16067-16569/1-574 | | | | | - | | | | Sagaing | Naga |
| 12 | Burman488 | M49 | 223 225 234 291 390 519 | 73 199 249d 263 309+C 315+C 489 | 16008-16569/1-574 | | | | | | | | 3682-4199=3780 | Sagaing | Naga |
| 12 | Burman489 | M9a1b1 | 158 223 234 362 519 | 73 150 152 263 309+C 315+C 489 | 16043-16569/1-574 | | | | | | | | 4237-4915=4491 4769 | Sagaing | Naga |
| 12 | Burman490 | D4g2a | 223 274 309 362 519 | 73 263 298 309+C 315+C 489 | 16001-16569/1-574 | | - | - | | | | | | Sagaing | Naga |
| 12 | Burman491 | G | 124 223 244C 246T 295 356 362 519 | 73 200 263 315+CC 468 489 521 | 16063-16569/1-573 | | + | + | | | | | | Sagaing | Naga |
| 12 | Burman492 | M49 | 223 225 234 291 390 519 | 73 199 249d 263 309+C 315+C 489 | 16043-16569/1-574 | | | | | | | | 3682-4290=3780 | Sagaing | Naga |
| 12 | Burman493 | M9a | 223 234 362 519 | 73 150 152 153 263 315+C 489 | 16033-16569/1-574 | | | | | | | | 4238-4962=4491 4769 | Sagaing | Naga |
| 12 | Burman494 | G | 124 223 244C 246T 295 356 362 519 | 73 200 263 315+CC 468 489 521 | 16043-16569/1-574 | | + | + | | | | | | Sagaing | Naga |
| 12 | Burman495 | M9a1b1 | 158 223 234 362 519 | 73 150 152 263 309+C 315+C | 16043-16569/1-437 | | | | | | | | 4230-4947=4491 4769 | Sagaing | Naga |
| 12 | Burman496 | M9a | 129 223 234 362 519 | 73 150 152 153 263 315+C 489 | 16018-16569/1-574 | | | | | | | | 4234-4863=4491 4769 | Sagaing | Naga |
| 12 | Burman497 | M9a | 129 223 234 362 519 | 73 150 152 153 263 315+C 489 | 16044-16569/1-574 | | | | | | | | 4239-4961=4491 4769 | Sagaing | Naga |
| 12 | Burman498 | M9a | 223 234 362 519 | 73 150 152 153 263 315+C 489 | 16040-16569/1-574 | | | | | | | | 4236-4961=4491 4769 | Sagaing | Naga |
| 12 | Burman499 | B5a | 140 183C 189 261 266A 519 | 73 152 210 263 309+C 315+C | 16012-16569/1-316 | | | | | | | | 7913-8471=8281-8289d | Sagaing | Naga |
| 12 | Burman500 | D | 223 311 362 | 73 146 263 309+C 315+C 316 489 | 16043-16569/1-574 | | | | | | | | 4521-5072=4769 4883; 15013-15632=15043 15301 15326 | Sagaing | Naga |
| 12 | Burman501* | M83 | 223 320 | 73 146 207 263 315+C | 16032-16569/1-316 | | | | | | | | | Sagaing | Naga |
| 12 | Burman502* | R31 | 218 289 304 526 | 73 152 183 185 263 315+CC | 16040-16569/1-574 | | | | | | | | | Sagaing | Naga |
| 12 | Burman503 | M9a | 223 234 243 362 519 | 73 150 152 153 263 315+C 489 | 16043-16569/1-526 | | | | | | | | 4237-4961=4491 4769 | Sagaing | Naga |
| 12 | Burman504* | M55 | 148 217 223 319 381 519 | 73 94 173 204 263 309+C 315+C 482 489 | 16043-16569/1-507 | | | | | | | | | Sagaing | Naga |
| 12 | Burman505 | M9a1b1 | 158 223 234 362 519 | 73 150 152 263 309+C 315+C 489 | 16024-16569/1-574 | | | | | | | | 4228-4775=4491 4769 | Sagaing | Naga |
| 12 | Burman506 | G | 124 223 244C 246T 295 356 362 519 | 73 200 263 315+CC 489 521 | 16040-16569/1-574 | | + | + | | | | | | Sagaing | Naga |
| 12 | Burman507 | M55 | 148 217 223 319 381 519 | 73 94 173 204 263 309+C 315+C 482 489 | 16061-16569/1-574 | | | | | | | | 9223-9650=9447 9540 | Sagaing | Naga |
| 12 | Burman508 | B5a | 140 183C 189 261 266A 519 | 73 152 210 263 309+C 315+C | 16061-16569/1-319 | + | | | | | | | | Sagaing | Naga |
| 12 | Burman509 | B5a | 140 183C 189 261 266A 519 | 73 152 189 210 263 309+CC 315+C | 16014-16569/1-319 | + | | | | | | | | Sagaing | Naga |
| 12 | Burman510 | M31 | 093 136 223 | 73 146 152 263 315+C 489 | 16043-16569/1-504 | | | | | | | | | Sagaing | Naga |
| 12 | Burman511 | B5a | 140 183C 189 261 266A 519 | 73 152 210 263 309+C 315+C 522-523d | 16042-16569/1-574 | + | | | | | | | | Sagaing | Naga |
| 12 | Burman512 | M83 | 129 223 320 | 73 146 263 309+CC 315+C 489 | 16043-16569/1-316/374-574 | | | | | | | | 7904-8479=8143 8271 8307 | Sagaing | Naga |
| 12 | Burman513 | M83 | 223 320 | 73 146 207 263 315+C 356+C 489 | 16023-16569/1-573 | | | | | | | | 7909-8607=8143 8271 8307 | Sagaing | Naga |
| 12 | Burman514 | M9a | 223 234 362 519 | 73 150 152 153 263 315+C 489 | 16042-16569/1-574 | | | | | | | | 4236-4888=4491 4769 | Sagaing | Naga |
| 13 | Burman001 | M74 | 093 146 223 311 362 519 | 73 263 315+C 489 | 16013-16569/1-574 | | | | | | | | 4519-5175=4769 5054; 15010-15547=15043 15301 15326 | Magway | Rakhine |
| 13 | Burman002 | F1c | 111 129 189 304 519 | 73 152 185 234 249d 263 315+C 522-523d | 16013-16569/1-575 | | | | | - | | | | Magway | Rakhine |
| 13 | Burman003 | M7b | 129 223 297 319 | 73 150 195 199 263 315+C 489 | 16007-16569/1-574 | | | | + | | | | | Magway | Rakhine |
| 13 | Burman004 | M9a1b1 | 158 223 234 362 519 | 73 146 150 152 153 263 315+C 489 | 16018-16569/1-575 | | | | - | | | | 4229-4833=4491 4769 | Magway | Rakhine |
| 13 | Burman005 | A | 223 256 290 319 362 | 73 151 152 200 235 263 315+C 522-523d | 16007-16569/1-574 | | | | | | | | | Magway | Rakhine |
| 13 | Burman006 | M6 | 218 223 231 356 362 519 | 73 263 315+C 461 489 522-523d | 16014-16569/1-575 | | | | | | | | 4925-5328=5082 5301 | Magway | Rakhine |
| 13 | Burman007 | D5 | 189 223 362 519 | 73 150 309+CC 315+C | 16007-16569/1-396 | | - | - | | | | | | Magway | Rakhine |
| 13 | Burman008 | M6 | 218 223 231 356 362 519 | 73 263 315+C 461 489 522-523d | 16008-16569/1-574 | | | | | | | | 4960-5323=5082? 5301 | Magway | Rakhine |
| 13 | Burman009 | F1c | 111 129 304 519 | 73 152 234 249d 263 315+C | 16008-16569/1-522 | | | | | - | | | | Magway | Rakhine |
| 13 | Burman010 | N21 | 093 182 193 223 260 519 | 73 150 195 263 309+C 315+C 337d | 16008-16569/1-574 | | | | | | | | 13084-13627=13437 | Magway | Rakhine |
| 13 | Burman011 | F1a | 129 172 295 304 519 | 73 200 249d 263 315+C 522-523d | 16008-16569/1-540 | | | | | - | | | | Magway | Rakhine |
| 13 | Burman012* | M84 | 183d 223 224 258d 272 519 | 73 146 185 263 309+C 315+C | 16006-16569/1-408 | | | | | | | | | Magway | Rakhine |
| 13 | Burman013* | M49 | 223 234 302 519 | 73 263 309+C 315+C 489 498d | 16007-16569/1-508 | | | | | | | | | Magway | Rakhine |
| 13 | Burman014 | M7b | 129 223 297 319 | 73 150 195 199 263 315+C 489 | 16008-16569/1-574 | | | | + | | | | | Magway | Rakhine |
| 13 | Burman015 | M49 | 223 234 302 519 | 73 263 309+C 315+C 489 498d | 16007-16569/1-574 | | | | | | | | 3683-4399=3780 | Magway | Rakhine |
| 13 | Burman016 | R11 | 182C 183C 189 311 390 399 519 | 73 185 189 263 309+CC 315+C | 16008-16569/1-365 | | | | | | | | 12598-12959=12950 | Magway | Rakhine |
| 13 | Burman017* | M38 | 189 223 242A | 73 146 152 246 263 309+C 315+C 455+T 489 513 | 16007-16569/1-575 | | | | | | | | | Magway | Rakhine |
| 13 | Burman018 | M7b | 129 223 297 357 | 73 150 152 199 263 315+C 489 | 16007-16569/1-574 | | | | + | | | | | Magway | Rakhine |
| 13 | Burman019 | M30 | 223 234 356 362 519 | 73 152 195A 263 315+C 489 522-523d | 16029-16569/1-575 | | + | + | | | | | | Magway | Rakhine |
| 13 | Burman020 | A | 223 290 319 | 64 73 146 151 235 263 309+C 315+C | 16013-16569/1-319 | | | | | | | | | Magway | Rakhine |

| Code | Sample nam | Haplogroup | HVS-I (16000+) | HVS-II | Readable Region | 9bp | 4831*Hha*1 | 5176*Alu*1 | 9820*Hinf*1 | 12406*Hpa*1 | 13262*Alu*1 | 14465*Acc*1 | Coding-Region Polymorphisms | Location | Nation |
|---|---|---|---|---|---|---|---|---|---|---|---|---|---|---|---|
| 13 | Burman021 | M7b | 129 189 213 223 297 519 | 73 150 199 263 309+C 315+C | 16005-16569/1-316 | | | | + | | | | | Magway | Rakhine |
| 13 | Burman022 | B5a | 140 182C 183C 189 250 261 266A 519 | 73 152 210 263 309+CCC 315+C | 16008-16569/1-316 | + | | | | | | | | Magway | Rakhine |
| 13 | Burman023 | D | 223 362 519 | 73 199 263 309+C 315+C | 16014-16569/1-316 | | - | - | | | | | | Magway | Rakhine |
| 13 | Burman024 | F1a1a | 093 108 129 162 172 259A 304 519 | 73 249d 263 315+C 482 522-523d | 16007-16569/1-574 | | | | | - | | | | Magway | Rakhine |
| 14 | Burman792 | D | 092 188 223 311 362 | 73 94 189 214 263 315+C 489 | 16014-16569/1-574 | | | | | | | | 15012-15730=15043 15301 15326 | Rakhine | Rakhine |
| 14 | Burman793 | M7a | 093 145 169 209 223 266 317T 324 362 519 | 73 146 199 263 315+C 489 520 | 16018-16569/1-574 | | | | + | | | | | Rakhine | Rakhine |
| 14 | Burman794 | M7h | 086 129 223 311 | 73 263 315+C 489 | 16001-16569/1-574 | | | | | | | | 9817-10041=9824; 12060-12440=12405 | Rakhine | Rakhine |
| 14 | Burman795 | B5a | 140 182C 183C 189 234 266A 519 | 73 150 210 263 309+CC 315+C | 16016-16569/1-316 | + | | | | | | | | Rakhine | Rakhine |
| 14 | Burman796 | B5a | 140 182C 183C 189 234 266A 519 | 73 150 210 263 309+CC 315+C | 16013-16569/1-318 | + | | | | | | | | Rakhine | Rakhine |
| 14 | Burman797 | B5a | 140 182C 183C 189 234 266A 519 | 73 150 210 263 309+CC 315+C | 16008-16569/1-323 | + | | | | | | | | Rakhine | Rakhine |
| 14 | Burman798 | D | 092 188 223 311 362 | 73 94 189 214 263 315+C 489 | 16012-16569/1-574 | | | | | | | | 4520-5275=4769 4883 5178A; 15013-15731=15043 15301 15326 | Rakhine | Rakhine |
| 14 | Burman799 | G | 174 223 239 362 | 73 214 263 315+C 356+C 489 | 16012-16569/1-574 | | | | | | | | | Rakhine | Rakhine |
| 14 | Burman800 | M7h | 086 129 223 311 | 73 263 315+C 489 | 16018-16569/1-575 | | | | | | | | 9817-9986=9824; 12059-12543=12405; 15011-15815=15043 15301 15326 15676 | Rakhine | Rakhine |
| 14 | Burman801* | M83 | 311 319 357 | 73 152 263 315+C 356+C 489 | 16012-16569/1-504 | | | | | | | | | Rakhine | Rakhine |
| 14 | Burman802 | M7h | 086 129 223 311 | 73 263 315+C 489 | 16012-16569/1-599 | | | | | | | | 9818-10197=9824; 12059-12410=12405; 15011-15746=15043 15301 15326 15676 | Rakhine | Rakhine |
| 14 | Burman803 | F1a1 | 129 162 172 189 304 488 519 | 73 249d 263 309+C 315+C | 16009-16569/1-323 | | | | | - | | | | Rakhine | Rakhine |
| 14 | Burman804 | M7h | 086 129 223 311 | 73 263 315+C 489 | 16012-16569/1-508 | | | | | | | | 12065-12511=12405; 15012-15854=15043 15301 15326 15676 | Rakhine | Rakhine |
| 14 | Burman805 | D | 092 188 223 311 362 | 73 94 189 214 263 315+C | 16012-16569/1-328 | | | | | | | | | Rakhine | Rakhine |
| 14 | Burman806 | M7h | 086 129 223 311 | 73 263 315+C 489 | 16012-16569/1-576 | | | | | | | | 9812-10304=9824; 12061-12430=12405; 15011-15728=15043 15301 15326 15676 | Rakhine | Rakhine |
| 14 | Burman807 | M7h | 086 129 223 311 | 73 263 315+C 489 | 16015-16569/1-575 | | | | | | | | 9817-10305=9824; 12059-12480=12405; 15008-15765=15043 15301 15326 15676 | Rakhine | Rakhine |
| 14 | Burman808 | M9a1b1 | 158 223 234 362 519 | 73 150 152 153 263 315+C 318 489 | 16017-16569/1-600 | | | | | | | | 4237-4770=4491 4769 | Rakhine | Rakhine |
| 14 | Burman809 | M7h | 086 129 223 311 | 73 263 315+C 489 | 16022-16569/1-593 | | | | | | | | 9817-10305=9824; 12060-12420=12405; 15011-15705=15043 15301 15326 15676 | Rakhine | Rakhine |
| 14 | Burman810* | M7h | 086 129 223 311 | 73 263 315+C 489 | 16017-16569/1-560 | | | | | | | | 15012-15734=15043 15301 15326 15676 | Rakhine | Rakhine |
| 14 | Burman811 | M83 | 311 319 357 | 73 152 263 315+C 356+C 489 | 16024-16569/1-512 | | | | | | | | 15407-16052=15670 15941 | Rakhine | Rakhine |
| 14 | Burman812 | B5a | 140 182C 183C 189 261 266A 304 519 | 73 152 210 263 309+CC 315+C | 16009-16569/1-319 | + | | | | | | | | Rakhine | Rakhine |
| 14 | Burman813 | M7h | 086 129 223 301 311 | 73 263 315+C 489 | 16017-16569/1-598 | | | | | | | | 9818-10300=9824; 12060-12430=12405; 12058-12540=12106 12308 12372; 15009-15800=15043 15301 15326 15676 | Rakhine | Rakhine |
| 14 | Burman814* | M35b | 223 519 | 73 199 263 315+C 489 | 16012-16569/1-599 | | | | | | | | | Rakhine | Rakhine |
| 14 | Burman815 | D5a2 | 092 172 182C 183C 189 223 266 362 | 73 150 263 309+C 315+C | 16033-16569/1-323 | | - | - | | | | | | Rakhine | Rakhine |
| 14 | Burman816 | M7h | 086 129 223 311 | 73 263 315+C 489 | 16018-16569/1-497 | | | | | | | | 9817-10170=9824; 12064-12430=12405; 15010-15782=15043 15301 15326 15676 | Rakhine | Rakhine |
| 14 | Burman817 | F1c | 111 129 304 519 | 73 152 234 249d 263 315+C | 16022-16569/1-360 | | | | | - | | | | Rakhine | Rakhine |
| 14 | Burman818 | M83 | 311 319 357 | 73 152 263 315+C 356+C 489 | 16009-16569/1-509 | | | | | | | | 15426-15710=15670 | Rakhine | Rakhine |
| 14 | Burman819 | B4c2 | 183C 184A 189 217 235 519 | 73 263 309+C | 16015-16569/1-310 | + | | | | | | | | Rakhine | Rakhine |
| 14 | Burman820* | M2c | 182C 183C 189 223 227 258C 263 | 73 263 447G 489 | 16031-16569/1-504 | | | | | | | | | Rakhine | Rakhine |
| 14 | Burman821 | G | 174 223 239 362 | 73 214 263 315+C 356+C 489 | 16015-16569/1-595 | | + | + | | | | | | Rakhine | Rakhine |
| 14 | Burman822* | M30c | 166d 167 223 519 | 73 146 195A 263 315+C 489 522-523d | 16022-16521/44-596 | | | | | | | | | Rakhine | Rakhine |
| 14 | Burman823 | B5a | 140 182C 183C 189 261 266A 304 519 | 73 152 210 263 309+CC | 16017-16569/1-313 | + | | | | | | | | Rakhine | Rakhine |
| 14 | Burman824 | B5a | 140 182C 183C 189 261 266A 304 519 | 73 152 210 263 309+CCC | 16019-16569/1-313 | + | | | | | | | | Rakhine | Rakhine |
| 14 | Burman825 | M24 | 086 223 278 519 | 73 146 195 263 309+C 315+C 489 | 16021-16569/1-508 | | | | | | | | 5811-6480=rCRS | Rakhine | Rakhine |
| 14 | Burman826 | B4c2 | 183C 184A 189 217 235 519 | 73 263 309+CC 315+C | 16016-16569/1-316 | | | | | | | | | Rakhine | Rakhine |
| 14 | Burman827 | N21 | 182 193 223 260 519 | 73 150 195 263 309+C 315+C 337d 750 | 16012-16569/1-784 | | | | | | | | 13084-13522=13437 | Rakhine | Rakhine |
| 14 | Burman828 | M20 | 086 129 209 223 272 519 | 73 152 225 249d 263 315+C 316 | 16011-16569/1-329 | | | | | | | | 12054-12395=12354; 14086-14291=14110? | Rakhine | Rakhine |
| 14 | Burman829 | D | 092 189 223 311 362 | 73 94 263 | 16012-16569/1-269 | | - | - | | | | | | Rakhine | Rakhine |
| 14 | Burman830 | D | 092 188 223 311 362 | 73 94 189 214 263 315+C 489 | 16013-16569/1-599 | | | | | | | | 4520-5404=4769 4883 5178A | Rakhine | Rakhine |
| 14 | Burman831 | M20 | 086 129 209 223 272 519 | 73 152 225 | 16011-16569/1-235 | | | | | | | | 12053-12637=12354; 14081-14616=14110 | Rakhine | Rakhine |
| 14 | Burman832 | M7a | 093 145 169 209 223 266 317T 324 362 519 | 73 146 199 263 309+C 315+C | 16012-16569/1-316 | | | | + | | | | | Rakhine | Rakhine |
| 14 | Burman833 | M7b | 223 297 | 73 150 199 204 263 309+C 315+C 489 | 16014-16569/1-499 | | | | + | | | | | Rakhine | Rakhine |
| 14 | Burman834 | M9a1b1 | 158 223 234 362 519 | 73 150 152 153 263 315+C | 16012-16569/1-316 | | | | | | | | 4236-4707=4491 4769 | Rakhine | Rakhine |
| 14 | Burman835 | N9a | 223 250 257A 261 311 | 73 150 194 263 309+C | 16017-16569/1-310 | | | | | | | | 4906-5409=5231 | Rakhine | Rakhine |
| 14 | Burman836 | D | 092 189 223 311 362 | 73 94 263 315+C 489 | 16016-16569/1-505 | | - | - | | | | | 6364-6905=rCRS | Rakhine | Rakhine |
| 14 | Burman837 | M12 | 129 172 223 290 519 | 73 | 16010-16569/1-85 | | | | | | | | 14613-15029=14727 14766 14783 15010 | Rakhine | Rakhine |
| 14 | Burman838 | M7a | 093 145 169 209 223 266 317T 324 362 519 | 73 146 199 263 309+C 315+C | 16027-16569/1-316 | | | | | | | | 6364-6907=rCRS; 9594-10136=rCRS | Rakhine | Rakhine |
| 14 | Burman839 | M7a | 093 145 169 209 223 266 317T 324 362 519 | 73 146 199 263 309+C 315+C | 16008-16569/1-316 | | | | + | | | | | Rakhine | Rakhine |
| 14 | Burman840* | M82 | 086 126 186 189 223 304 519 527 | 10 73 146 185 189 195 234 263 309+C 315+C | 16012-16569/1-467 | | | | | | | | | Rakhine | Rakhine |
| 14 | Burman841 | D4g2a | 169 223 266 274 362 | 73 263 298 309+C 315+C | 16027-16569/1-365 | | - | - | | | | | | Rakhine | Rakhine |
| 14 | Burman842 | M20 | 086 129 209 223 272 519 | 73 152 225 249d 263 309+C 315+C 316 489 | 16024-16569/1-522 | | | | | | | | 12036-12396=12354; 14091-14290=14110? | Rakhine | Rakhine |
| 14 | Burman843* | M49 | 153 213 223 234 257 294 519 527 | 73 263 309+C 315+C | 16025-16569/1-316 | | | | | | | | | Rakhine | Rakhine |
| 14 | Burman844 | M7h | 086 129 223 311 | 73 263 315+C 489 | 16033-16569/1-571 | | | | | | | | 9817-10197=9824; 12058-12500=12405; 15011-15649=15043 15301 15326 | Rakhine | Rakhine |
| 14 | Burman845 | M38 | 092 111 184 223 519 | 73 246 263 315+C | 16016-16569/1-474 | | | | | | | | 936-1603=1438; 3682-4308=4099 | Rakhine | Rakhine |
| 14 | Burman846 | M12 | 129 172 223 290 519 | 73 152 198 263 309+CC 315+C | 16010-16569/1-316 | | | | | | | | 14668-14924=14727 14766 14783 | Rakhine | Rakhine |
| 14 | Burman847 | M2c | 182C 183C 189 223 227 258C 263 | 73 263 447G 489 | 16018-16569/1-575 | | | | | | | | 15410-15949=15670 15929 | Rakhine | Rakhine |
| 14 | Burman848 | U2a | 051 206C 230 265T 304 311 456 519 | 73 263 309+C 315+C | 16003-16569/1-600 | | | | | - | | | 12059-12450=12308 12372 | Rakhine | Rakhine |
| 14 | Burman849* | M38 | 092 111 184 223 519 | 73 246 263 315+C 489 | 16021-16569/1-492 | | | | | | | | | Rakhine | Rakhine |
| 14 | Burman850 | M38 | 092 111 184 223 519 | 73 246 263 315+C 489 | 16043-16569/1-583 | | | | | | | | 15012-15571=15043 15301 15314 15326 15487 | Rakhine | Rakhine |
| 14 | Burman851 | D4g2a | 174 223 274 304 362 519 | 73 263 298 309+CC 315+C | 16017-16569/1-316 | | - | - | | | | | | Rakhine | Rakhine |

| Code | Sample nam | Haplogroup | HVS-I (16000+) | HVS-II | Readable Region | 9bp | 4831*Hha*1 | 5176*Alu*1 | 9820*Hinf*1 | 12406*Hpa*1 | 13262*Alu*1 | 14465*Acc*1 | Coding-Region Polymorphisms | Location | Nation |
|---|---|---|---|---|---|---|---|---|---|---|---|---|---|---|---|
| 14 | Burman852 | U2 | 051 114A | 73 146 215 263 309+C 315+C | 16042-16569/1-474 | | | | | | | | 12043-12354=12106 12308 | Rakhine | Rakhine |
| 14 | Burman853 | B4c2 | 183C 184A 189 217 235 519 | 73 263 309+CC 315+C | 16019-16569/1-316 | + | | | | | | | | Rakhine | Rakhine |
| 14 | Burman854 | M7b | 223 297 | 73 150 199 204 263 309+C 315+C 489 | 16018-16569/1-504 | | | | + | | | | | Rakhine | Rakhine |

Note:
1. Population code is consistent with those in Table S6.
2. Suffixe "*" means the mtDNA of the sample has been completely sequenced.

**Table S3. AMOVA results based on mtDNA haplogroup frequencies.**

| Groups | Number of populations | Number of groups | Molecular variation | | | | | | | | |
|---|---|---|---|---|---|---|---|---|---|---|---|
| | | | Among populations | | | Among populations within groups | | | Among groups | | |
| | | | % | Fst | P | % | Fsc | P | % | Fct | P |
| Language family (AA vs. TK vs. TB vs. HM) | 129 | 4 | 91.33 | 0.08871 | $<10^{-5}$ | 6.95 | 0.07090 | $<10^{-5}$ | 1.92 | 0.01917 | $<10^{-5}$ |

**Table S4. Admixture analysis of the Myanmar populations by comparing with their potential parental populations.**

| | Hybrid populations | | | | | | | |
|---|---|---|---|---|---|---|---|---|
| Parental groups | Burmans_1 | Burmans_2 | Burmans_3 | Burmans_4 | Burmans_5 | Burmans_6 | Naga_1 | Naga_2 |
| TB_Bangladesh | 0.000±0.106 | 0.247±0.097 | 0.252±0.130 | 0.063±0.102 | 0.338±0.080 | 0.212±0.094 | 0.000±0.178 | 0.144±0.150 |
| TB_India | 0.000±0.134 | 0.116±0.121 | 0.212±0.163 | 0.379±0.128 | 0.189±0.100 | 0.000±0.118 | 0.903±0.223 | 0.129±0.189 |
| TB_Thailand | 0.000±0.188 | 0.000±0.170 | 0.065±0.229 | 0.079±0.180 | 0.032±0.141 | 0.000±0.166 | 0.000±0.314 | 0.000±0.265 |
| TB_Tibet and QH | 0.001±0.151 | 0.000±0.137 | 0.182±0.184 | 0.050±0.145 | 0.016±0.113 | 0.001±0.133 | 0.000±0.251 | 0.194±0.213 |
| TB_SWC | 0.000±0.332 | 0.259±0.301 | 0.000±0.405 | 0.146±0.318 | 0.189±0.249 | 0.000±0.293 | 0.000±0.554 | 0.533±0.469 |
| AA_Cambodia | 0.000±0.117 | 0.000±0.106 | 0.000±0.143 | 0.000±0.112 | 0.000±0.087 | 0.000±0.103 | 0.000±0.195 | 0.000±0.165 |
| AA_India | 0.344±0.133 | 0.000±0.120 | 0.057±0.162 | 0.048±0.127 | 0.000±0.099 | 0.002±0.117 | 0.000±0.221 | 0.000±0.187 |
| AA_Malaysia | 0.000±0.083 | 0.094±0.075 | 0.067±0.102 | 0.132±0.080 | 0.081±0.062 | 0.000±0.073 | 0.019±0.139 | 0.000±0.118 |
| AA_Thailand | 0.000±0.151 | 0.000±0.137 | 0.000±0.184 | 0.000±0.145 | 0.000±0.113 | 0.000±0.133 | 0.003±0.252 | 0.000±0.213 |
| AA_Vietnam | 0.398±0.365 | 0.000±0.331 | 0.000±0.446 | 0.000±0.351 | 0.000±0.274 | 0.000±0.322 | 0.000±0.610 | 0.000±0.516 |
| AA_Yunnan | 0.201±0.122 | 0.218±0.110 | 0.025±0.149 | 0.000±0.117 | 0.155±0.091 | 0.000±0.108 | 0.000±0.203 | 0.000±0.172 |
| HM_South China | 0.057±0.444 | 0.000±0.403 | 0.000±0.543 | 0.000±0.426 | 0.000±0.333 | 0.000±0.392 | 0.000±0.742 | 0.000±0.627 |
| HM_SWC | 0.000±0.187 | 0.052±0.170 | 0.140±0.229 | 0.000±0.180 | 0.000±0.140 | 0.000±0.165 | 0.000±0.313 | 0.000±0.265 |
| TK_SWC | 0.000±0.208 | 0.015±0.189 | 0.000±0.255 | 0.104±0.200 | 0.000±0.156 | 0.785±0.184 | 0.075±0.348 | 0.000±0.294 |
| TK_Thailand | 0.000±0.403 | 0.000±0.366 | 0.000±0.493 | 0.000±0.387 | 0.000±0.302 | 0.000±0.356 | 0.000±0.674 | 0.000±0.570 |

| | Hybrid populations | | | | | | | |
|---|---|---|---|---|---|---|---|---|
| Parental groups | Naga_3 | Chin_1 | Chin_2 | Chin_3 | Rakhine_1 | Rakhine_2 | Bamar_S | Karen_S |
| TB_Bangladesh | 0.000±0.129 | 0.476±0.151 | 0.463±0.121 | 0.083±0.178 | 0.106±0.097 | 0.026±0.091 | 0.186±0.047 | 0.000±0.122 |
| TB_India | 0.000±0.162 | 0.341±0.190 | 0.285±0.152 | 0.397±0.224 | 0.201±0.121 | 0.000±0.115 | 0.104±0.059 | 0.000±0.153 |
| TB_Thailand | 0.000±0.227 | 0.183±0.267 | 0.057±0.214 | 0.156±0.315 | 0.000±0.170 | 0.000±0.161 | 0.081±0.083 | 0.000±0.215 |
| TB_Tibet and QH | 0.531±0.182 | 0.000±0.214 | 0.191±0.172 | 0.057±0.252 | 0.046±0.137 | 0.000±0.129 | 0.048±0.066 | 0.000±0.172 |
| TB_SWC | 0.000±0.401 | 0.000±0.472 | 0.000±0.379 | 0.000±0.556 | 0.000±0.301 | 0.000±0.285 | 0.103±0.146 | 0.162±0.379 |
| AA_Cambodia | 0.000±0.141 | 0.000±0.166 | 0.000±0.133 | 0.000±0.195 | 0.000±0.106 | 0.000±0.100 | 0.000±0.051 | 0.033±0.133 |
| AA_India | 0.414±0.160 | 0.000±0.189 | 0.000±0.151 | 0.004±0.222 | 0.071±0.120 | 0.139±0.114 | 0.016±0.058 | 0.000±0.152 |
| AA_Malaysia | 0.016±0.101 | 0.000±0.118 | 0.000±0.095 | 0.000±0.139 | 0.029±0.075 | 0.000±0.071 | 0.035±0.037 | 0.074±0.095 |
| AA_Thailand | 0.000±0.182 | 0.000±0.214 | 0.000±0.172 | 0.071±0.253 | 0.000±0.137 | 0.000±0.129 | 0.000±0.066 | 0.007±0.172 |
| AA_Vietnam | 0.039±0.442 | 0.000±0.519 | 0.000±0.417 | 0.000±0.612 | 0.000±0.331 | 0.000±0.313 | 0.000±0.161 | 0.000±0.417 |
| AA_Yunnan | 0.000±0.147 | 0.000±0.173 | 0.004±0.139 | 0.231±0.204 | 0.107±0.111 | 0.000±0.105 | 0.034±0.054 | 0.444±0.139 |
| HM_South China | 0.000±0.537 | 0.000±0.631 | 0.000±0.507 | 0.000±0.744 | 0.000±0.403 | 0.000±0.381 | 0.057±0.196 | 0.000±0.508 |
| HM_SWC | 0.000±0.227 | 0.000±0.267 | 0.000±0.214 | 0.000±0.314 | 0.000±0.170 | 0.000±0.161 | 0.010±0.083 | 0.000±0.214 |
| TK_SWC | 0.000±0.252 | 0.000±0.296 | 0.000±0.238 | 0.000±0.349 | 0.439±0.189 | 0.835±0.179 | 0.000±0.092 | 0.000±0.238 |
| TK_Thailand | 0.000±0.488 | 0.000±0.573 | 0.000±0.460 | 0.000±0.676 | 0.000±0.366 | 0.000±0.346 | 0.326±0.178 | 0.280±0.461 |

Note: TB: Tibeto-Burman; AA: Austro-Asiatic; HM:Hmong-Mien; TK: Tai-Kadai; QH: Qinghai province, China; SWC: southwestern China. Bamar_S and Karen_S came from Summerer's study[1].

Reference for Table S4

1. Summerer M, *et al*. Large-scale mitochondrial DNA analysis in Southeast Asia reveals evolutionary effects of cultural isolation in the multi-ethnic population of Myanmar. *BMC Evol. Biol.* **14,** 17 (2014).

**Supplementary Table S6. Information of 129 populations analysed in the present study.**

| Code | Population | Location | Country | Longitude | Latitude | Language | Size | Reference |
|---|---|---|---|---|---|---|---|---|
| 1 | Burmans_1 | Sagaing | Myanmar | 95.43 | 21.83 | Tibeto-Burman | 32 | This study |
| 2 | Burmans_2 | Sagaing | Myanmar | 94.05 | 23.36 | Tibeto-Burman | 53 | This study |
| 3 | Burmans_3 | Sagaing | Myanmar | 94.56 | 23.84 | Tibeto-Burman | 51 | This study |
| 4 | Burmans_4 | Magway | Myanmar | 94.58 | 20.56 | Tibeto-Burman | 122 | This study |
| 5 | Burmans_5 | Bago | Myanmar | 95.74 | 17.78 | Tibeto-Burman | 69 | This study |
| 6 | Burmans_6 | Ayeyarwady | Myanmar | 95.25 | 18.35 | Tibeto-Burman | 72 | This study |
| 7 | Chin_1 | Chin | Myanmar | 93.69 | 23.48 | Tibeto-Burman | 58 | This study |
| 8 | Chin_2 | Chin | Myanmar | 93.45 | 21.56 | Tibeto-Burman | 187 | This study |
| 9 | Chin_3 | Magway | Myanmar | 94.26 | 20.15 | Tibeto-Burman | 13 | This study |
| 10 | Naga_1 | Sagaing | Myanmar | 95.30 | 25.90 | Tibeto-Burman | 32 | This study |
| 11 | Naga_2 | Sagaing | Myanmar | 94.92 | 25.50 | Tibeto-Burman | 30 | This study |
| 12 | Naga_3 | Sagaing | Myanmar | 94.98 | 25.18 | Tibeto-Burman | 39 | This study |
| 13 | Rakhine_1 | Magway | Myanmar | 94.10 | 21.70 | Tibeto-Burman | 24 | This study |
| 14 | Rakhine_2 | Rakhine | Myanmar | 94.78 | 18.69 | Tibeto-Burman | 63 | This study |
| 15 | Adi | Assam | India | 92.92 | 26.30 | Tibeto-Burman | 45 | 1 |
| 16 | Apatani | Arunachal Pradesh | India | 94.06 | 28.31 | Tibeto-Burman | 26 | 1 |
| 17 | Apatani | Tripura | India | 91.99 | 23.99 | Tibeto-Burman | 21 | 1 |
| 18 | Naga | Nagaland | India | 94.72 | 26.19 | Tibeto-Burman | 43 | 1 |
| 19 | Nishi | Tripura | India | 91.99 | 23.99 | Tibeto-Burman | 44 | 1 |
| 20 | Tipperah | Tripura | India | 91.99 | 23.99 | Tibeto-Burman | 20 | 2 |

| Code | Population | Location | Country | Longitude | Latitude | Language | Size | Reference |
|---|---|---|---|---|---|---|---|---|
| 21 | Garo | Meghlaya | India | 90.55 | 25.58 | Tibeto-Burman | 76 | 3 |
| 22 | Lyngngam | Meghlaya | India | 90.92 | 25.38 | Austro-Asiatic | 74 | 3 |
| 23 | Nongtrai | Meghlaya | India | 91.13 | 25.85 | Austro-Asiatic | 27 | 3 |
| 24 | Maram | Meghlaya | India | 91.23 | 25.50 | Austro-Asiatic | 60 | 3 |
| 25 | Bhoi | Meghlaya | India | 91.75 | 25.96 | Austro-Asiatic | 29 | 3 |
| 26 | Khynriam | Meghlaya | India | 91.79 | 25.54 | Austro-Asiatic | 82 | 3 |
| 27 | War_Khas | Meghlaya | India | 91.94 | 25.21 | Austro-Asiatic | 29 | 3 |
| 28 | Pnar | Meghlaya | India | 92.31 | 25.47 | Austro-Asiatic | 51 | 3 |
| 29 | War_Jaint | Meghlaya | India | 92.32 | 25.20 | Austro-Asiatic | 17 | 3 |
| 30 | Tibetan | Nagqu, Tibet | China | 92.04 | 31.64 | Tibeto-Burman | 168 | 4 |
| 31 | Tibetan | Rikaze, Tibet | China | 88.90 | 29.40 | Tibeto-Burman | 220 | 4 |
| 32 | Tibetan | Liangshan, Sichuan | China | 102.27 | 27.89 | Tibeto-Burman | 62 | 4 |
| 33 | Tibetan | Guide, Qinghai | China | 101.43 | 36.05 | Tibeto-Burman | 76 | 4 |
| 34 | Tibetan | Chamdo, Tibet | China | 97.17 | 31.27 | Tibeto-Burman | 61 | 5 |
| 35 | Tibetan | Garze, Sichuan | China | 101.96 | 30.06 | Tibeto-Burman | 55 | 5 |
| 36 | Tibetan | Lhasa, Tibet | China | 91.14 | 29.65 | Tibeto-Burman | 59 | 5 |
| 37 | Tibetan | Nagqu, Tibet | China | 92.04 | 31.75 | Tibeto-Burman | 58 | 5 |
| 38 | Monba | Nyingchi, Tibet | China | 94.39 | 29.71 | Tibeto-Burman | 51 | 5 |
| 39 | Tibetan | Nyingchi, Tibet | China | 94.28 | 29.43 | Tibeto-Burman | 53 | 5 |
| 40 | Lhoba | Shannan, Tibet | China | 91.75 | 29.28 | Tibeto-Burman | 20 | 5 |
| 41 | Tibetan | Shannan, Tibet | China | 91.78 | 28.84 | Tibeto-Burman | 56 | 5 |

| Code | Population | Location | Country | Longitude | Latitude | Language | Size | Reference |
|---|---|---|---|---|---|---|---|---|
| 42 | Tibetan | Rikaze, Tibet | China | 88.62 | 28.87 | Tibeto-Burman | 59 | 5 |
| 43 | Hani-YN | Xishuangbanna, Yunnan | China | 100.80 | 22.04 | Tibeto-Burman | 80 | 6 |
| 44 | Bai-YN1 | Dali | China | 100.23 | 25.73 | Tibeto-Burman | 69 | 6, 7 |
| 45 | Bai-YN2 | Xishuangbanna | China | 100.81 | 22.05 | Tibeto-Burman | 19 | 6 |
| 46 | Yi-YN1 | Xishuangbanna | China | 100.80 | 22.06 | Tibeto-Burman | 16 | 6 |
| 47 | Yi-YN2 | Chuxiong | China | 101.53 | 25.05 | Tibeto-Burman | 40 | 6 |
| 48 | Jino-YN | Xishuangbanna | China | 100.80 | 22.05 | Tibeto-Burman | 18 | 6 |
| 49 | Lahu-YN | Simao, Xishuangbanna | China | 100.97 | 22.80 | Tibeto-Burman | 37 | 6, 8, 9 |
| 50 | Pumi-YN | Ninglang | China | 100.85 | 27.31 | Tibeto-Burman | 35 | 6 |
| 51 | Naxi-YN | lijiang | China | 100.23 | 26.88 | Tibeto-Burman | 45 | 6 |
| 52 | Zang-YN1 | Diqing | China | 99.70 | 27.82 | Tibeto-Burman | 88 | 4, 9 |
| 53 | Zang-YN2 | Zhongdian | China | 99.70 | 27.86 | Tibeto-Burman | 35 | 6 |
| 54 | Lisu-YN | Gongshan | China | 98.66 | 27.76 | Tibeto-Burman | 30 | 7 |
| 55 | Va-1 | Simao | China | 100.98 | 22.80 | Austro-Asiatic | 22 | 9 |
| 56 | Va-1 | Gengma,Ximeng | China | 99.40 | 23.57 | Austro-Asiatic | 36 | 8 |
| 57 | Bugan | Xichou | China | 104.67 | 23.45 | Austro-Asiatic | 32 | 10 |
| 58 | Dai-YN1 | Jinghong | China | 100.80 | 22.05 | Tai-Kadai | 81 | 7, 10 |
| 59 | Dai-YN2 | Xishuangbanna | China | 100.79 | 22.04 | Tai-Kadai | 21 | 9 |
| 60 | Buyang-YN | Guangnan | China | 105.06 | 24.07 | Tai-Kadai | 31 | 10 |
| 61 | Lachi-YN | Maguan | China | 104.40 | 23.03 | Tai-Kadai | 30 | 10 |
| 62 | OT-YN | Malipo | China | 104.70 | 23.14 | Tai-Kadai | 25 | 10 |

| Code | Population | Location | Country | Longitude | Latitude | Language | Size | Reference |
|---|---|---|---|---|---|---|---|---|
| 63 | GL-YN | Malipo | China | 104.71 | 23.15 | Tai-Kadai | 14 | 10 |
| 64 | Miao-YN | Wenshan | China | 104.25 | 23.37 | Hmong-Mien | 39 | 11 |
| 65 | Yao-YN1 | Mengla | China | 101.57 | 21.49 | Hmong-Mien | 37 | 11 |
| 66 | Yao-YN2 | Malipo | China | 104.72 | 23.16 | Hmong-Mien | 40 | 11 |
| 67 | BY-GZ1 | Libo | China | 107.89 | 25.42 | Tai-Kadai | 33 | 10 |
| 68 | BY-GZ2 | Pingtang | China | 107.32 | 25.85 | Tai-Kadai | 30 | 10 |
| 69 | GL-GZ1 | Bijie | China | 105.28 | 27.33 | Tai-Kadai | 12 | 10 |
| 70 | GL-GZ2 | Majiang | China | 107.59 | 26.50 | Tai-Kadai | 29 | 10 |
| 71 | GL-GZ3 | Dafang | China | 105.61 | 27.16 | Tai-Kadai | 31 | 10 |
| 72 | Yao-HN1 | Jishou | China | 109.70 | 28.27 | Hmong-Mien | 103 | 11 |
| 73 | Yao-HN2 | Jianghua | China | 111.58 | 25.20 | Hmong-Mien | 24 | 11 |
| 74 | ML-GX | Luocheng | China | 108.90 | 24.80 | Tai-Kadai | 66 | 10, 12 |
| 75 | Maonan-GX | Huanjiang | China | 108.25 | 24.84 | Tai-Kadai | 32 | 10 |
| 76 | Caolan-GX | Fangcheng | China | 108.35 | 21.79 | Tai-Kadai | 30 | 10 |
| 77 | Sui-GX | Rongshui | China | 109.26 | 25.08 | Tai-Kadai | 30 | 10 |
| 78 | Zhuang-GX1 | Tianlin | China | 106.23 | 24.32 | Tai-Kadai | 25 | 10 |
| 79 | Zhuang-GX2 | Hezhou | China | 111.56 | 24.41 | Tai-Kadai | 55 | 12 |
| 80 | WS-GX | Rongshui | China | 109.26 | 25.09 | Tai-Kadai | 33 | 10 |
| 81 | Yerong-GX | Napo | China | 105.83 | 23.40 | Tai-Kadai | 15 | 10 |
| 82 | GL-GX | Longlin | China | 105.36 | 24.90 | Tai-Kadai | 30 | 10 |
| 83 | Dong-GX | Sanjiang | China | 109.60 | 25.79 | Tai-Kadai | 72 | 12 |

| Code | Population | Location | Country | Longitude | Latitude | Language | Size | Reference |
|---|---|---|---|---|---|---|---|---|
| 84 | Yao-GX1 | Dahua | China | 108.00 | 23.77 | Hmong-Mien | 19 | 11 |
| 85 | Yao-GX2 | Tianlin | China | 106.22 | 24.31 | Hmong-Mien | 64 | 11 |
| 86 | Yao-GX3 | Fuchuan | China | 111.28 | 24.82 | Hmong-Mien | 102 | 11, 12 |
| 87 | Yao-GX4 | Fangcheng | China | 108.38 | 21.80 | Hmong-Mien | 30 | 11 |
| 88 | Yao-GX5 | Hezhou | China | 111.58 | 24.43 | Hmong-Mien | 41 | 11 |
| 89 | Yao-GX6 | Shangsi | China | 107.98 | 22.17 | Hmong-Mien | 32 | 11 |
| 90 | Yao-GX7 | Jinxiu | China | 110.19 | 24.14 | Hmong-Mien | 67 | 12 |
| 91 | Yao-GD | Liannan | China | 107.98 | 22.17 | Hmong-Mien | 35 | 12 |
| 92 | Danga-HI | Lingshui | China | 110.04 | 18.51 | Tai-Kadai | 40 | 10 |
| 93 | Lingao-HI | Lingao | China | 109.69 | 19.91 | Tai-Kadai | 31 | 10 |
| 94 | Hlai-Qi-HI | Tongza | China | 109.52 | 18.78 | Tai-Kadai | 34 | 10 |
| 95 | Jiamao-HI | Baoting | China | 109.70 | 18.64 | Tai-Kadai | 27 | 10 |
| 96 | Cun-HI | Dongfang | China | 108.65 | 19.11 | Tai-Kadai | 30 | 10 |
| 97 | Kinh | Hanoi | Vietnam | 105.85 | 21.03 | Austro-Asiatic | 139 | 13 |
| 98 | Middle Viet | Middle Vietnam | Vietnam | 107.58 | 16.47 | Austro-Asiatic | 62 | 10 |
| 99 | Northern VIE1 | Hanoi | Vietnam | 105.90 | 21.10 | Austro-Asiatic | 187 | 14 |
| 100 | Northern VIE2 | Vietnam | Vietnam | 105.00 | 21.20 | Austro-Asiatic | 42 | 15 |
| 101 | Viet-South | South Vietnamese from California | Vietnam | 104.99 | 10.49 | Austro-Asiatic | 35 | 16 |
| 102 | Thai-KK | Khon Kaen | Thailand | 102.62 | 16.02 | Tai-Kadai | 44 | 17 |
| 103 | Phuthai-THA | Nakhon Pathom | Thailand | 100.17 | 13.99 | Tai-Kadai | 25 | 17 |
| 104 | LSg-THA | Suphan Buri | Thailand | 99.99 | 14.56 | Tai-Kadai | 25 | 17 |

| Code | Population | Location | Country | Longitude | Latitude | Language | Size | Reference |
|---|---|---|---|---|---|---|---|---|
| 105 | Thai-CM | Chiang Mai | Thailand | 98.67 | 18.88 | Tai-Kadai | 220 | 17, 18 |
| 106 | Thai-Jin | Thailand | Thailand | 99.67 | 17.25 | Tai-Kadai | 40 | 15 |
| 107 | Thai-Yao | Northern Thailand | Thailand | 99.34 | 18.85 | Tai-Kadai | 34 | 7 |
| 108 | Thai-Korat | Thailand | Thailand | 99.69 | 17.26 | Tai-Kadai | 32 | 19 |
| 109 | Akha | Chiang Rai | Thailand | 99.72 | 19.91 | Tibeto-Burman | 91 | 20 |
| 110 | Lahu | Chiang Mai | Thailand | 99.72 | 19.91 | Tibeto-Burman | 39 | 20 |
| 111 | Lisu | Chiang Mai | Thailand | 99.72 | 19.91 | Tibeto-Burman | 54 | 20 |
| 112 | Lisu | Chiang Rai | Thailand | 99.72 | 19.91 | Tibeto-Burman | 41 | 20 |
| 113 | Lisu | Mae Hong Son | Thailand | 97.87 | 18.78 | Tibeto-Burman | 25 | 20 |
| 114 | Mussur | Chiang Mai | Thailand | 99.72 | 19.91 | Tibeto-Burman | 21 | 17 |
| 115 | Lisu_4 | Chiang Mai | Thailand | 97.87 | 18.78 | Tibeto-Burman | 25 | 17 |
| 116 | Sakai | Trang | Thailand | 99.64 | 7.61 | Austro-Asiatic | 20 | 17 |
| 117 | Chong | Chanthaburi | Thailand | 102.16 | 12.82 | Austro-Asiatic | 25 | 17 |
| 118 | Khm | Northeast Thailand | Thailand | 102.16 | 12.82 | Austro-Asiatic | 22 | 19 |
| 119 | ChB | Northeast Thailand | Thailand | 102.25 | 14.93 | Austro-Asiatic | 20 | 19 |
| 120 | Cambodia | Siem Reap, NW cambodia | Cambodia | 103.86 | 13.37 | Austro-Asiatic | 31 | 21 |
| 121 | Semang | West Malaysia | Malaysia | 101.80 | 4.80 | Austro-Asiatic | 112 | 22 |
| 122 | Senoi | West Malaysia | Malaysia | 101.88 | 5.06 | Austro-Asiatic | 52 | 22 |
| 123 | Semelai | West Malaysia | Malaysia | 102.25 | 2.90 | Austro-Asiatic | 61 | 22 |
| 124 | Nicobarese | Eastern India | India | 92.46 | 10.64 | Austro-Asiatic | 46 | 23, 24 |
| 125 | Barma | Myanmar | Myanmar | 98.06 | 16.65 | Tibeto-Burman | 116 | 25 |

| Code | Population | Location | Country | Longitude | Latitude | Language | Size | Reference |
|---|---|---|---|---|---|---|---|---|
| 126 | Karen | Myanmar | Myanmar | 97.97 | 17.05 | Tibeto-Burman | 155 | 25 |
| 127 | Chakma | Chittagong hill tract | Bangladesh | 91.83 | 22.40 | Tibeto-Burman | 108 | 26 |
| 128 | Marma | Chittagong hill tract | Bangladesh | 91.80 | 22.37 | Tibeto-Burman | 97 | 26 |
| 129 | Tripura | Chittagong hill tract | Bangladesh | 91.82 | 22.29 | Tibeto-Burman | 97 | 26 |
| **Totally** | | | | | | | **6,752** | |

**References for Table S6**


1. Cordaux, R. *et al*. Mitochondrial DNA analysis reveals diverse histories of tribal populations from India. *Eur. J. Hum. Genet.* **11,** 253-264 (2003).
2. Roychoudhury, S *et al.* Genomic structures and population histories of linguistically distinct tribal groups of India. *Hum. genet.* **109,** 339-350 (2001).
3. Reddy, B. M. *et al.* Austro-Asiatic tribes of Northeast India provide hitherto missing genetic link between South and Southeast Asia. *PLoS ONE* **2,** e1141 (2007).
4. Zhao, M. *et al.* Mitochondrial genome evidence reveals successful Late Paleolithic settlement on the Tibetan Plateau. *Proc. Natl. Acad. Sci. USA* **106,** 21230-21235 (2009).
5. Qin, Z. D. *et al.* A Mitochondrial Revelation of Early Human Migrations to the Tibetan Plateau Before and After the Last Glacial Maximum. *Am. J. Phys. Anthropol.* **143,** 555-569 (2010).
6. Wen, B. *et al*. Analyses of genetic structure of Tibeto-Burman populations reveals sex-biased admixture in southern Tibeto-Burmans. *Am. J. Hum. Genet.* **74,** 856-865 (2004).
7. Yao, Y. G. *et al.* Genetic relationship of Chinese ethnic populations revealed by mtDNA sequence diversity. *Am. J. Phys. Anthropol.* **118,** 63-76 (2002).
8. Yao, Y. G. & Zhang, Y. P. Phylogeographic analysis of mtDNA variation in four ethnic populations from Yunnan Province: new data and a reappraisal. *J. Hum. Genet.* **47,** 311-318 (2002).
9. Qian, Y. P. *et al.* Mitochondrial DNA polymorphisms in Yunnan nationalities in China. *J. Hum. Genet.* **46,** 211-220 (2001).
10. Li, H. *et al.* Mitochondrial DNA diversity and population differentiation in southern East Asia. *Am. J. Phys. Anthropol.* **134,** 481-488 (2007).
11. Wen, B. *et al.* Genetic structure of Hmong-Mien speaking populations in East Asia as revealed by mtDNA lineages. *Mol. Biol. Evol* **22,** 725-734 (2005).

12. Gan, R. J. *et al.* Pinghua population as an exception of Han Chinese's coherent genetic structure. *J. Hum. Genet.* **53,** 303-313 (2008).
13. Peng, M. S., *et al.* Tracing the Austronesian Footprint in Mainland Southeast Asia: A Perspective from Mitochondrial DNA. *Mol. Biol. Evol.* **27,** 2417-2430 (2010).
14. Irwin, J. A. *et al.* Mitochondrial control region sequences from a Vietnamese population sample. *Int. J. Legal. Med.* **122,** 257-259 (2008).
15. Jin, H. J., Tyler-Smith, C. & Kim, W. The peopling of Korea revealed by analyses of mitochondrial DNA and Y-chromosomal markers. *PLoS ONE* **4,** e4210 (2009).
16. Oota, H. *et al.* Extreme mtDNA homogeneity in continental Asian populations. *Am. J. Phys. Anthropol.* **118,** 146-153 (2002).
17. Fucharoen, G., Fucharoen, S. & Horai, S. Mitochondrial DNA polymorphisms in Thailand. *J. Hum. Genet.* **46,** 115-125 (2001).
18. Zimmermann, B. *et al.* Forensic and phylogeographic characterization of mtDNA lineages from northern Thailand (Chiang Mai). *Int. J. Legal. Med.* **123,** 495-501 (2009).
19. Lertrit, P. *et al.* Genetic history of Southeast Asian populations as revealed by ancient and modern human mitochondrial DNA analysis. *Am. J. Phys. Anthropol.* **137,** 425-440 (2008).
20. Oota, H., Settheetham-Ishida, W., Tiwawech, D., Ishida, T. & Stoneking, M. Human mtDNA and Y-chromosome variation is correlated with matrilocal versus patrilocal residence. *Nat. Genet.* **29,** 20-21 (2001).
21. Black, M. L., Dufall, K., Wise, C., Sullivan, S. & Bittles, A. H. Genetic ancestries in northwest Cambodia. *Ann. Hum. Biol.* **33,** 620-627 (2006).
22. Hill, C. *et al.* Phylogeography and ethnogenesis of aboriginal Southeast Asians. *Mol. Biol. Evol.* **23,** 2480-2491 (2006).
23. Prasad, B. V. *et al.* Mitochondrial DNA variation in Nicobarese Islanders. *Hum. Biol.* **73,** 715-725 (2001).
24. Thangaraj, K. *et al.* Genetic affinities of the Andaman Islanders, a vanishing human population. *Curr. Biol.* **13,** 86-93 (2003).
25. Summerer, M. *et al.* Large-scale mitochondrial DNA analysis in Southeast Asia reveals evolutionary effects of cultural isolation in the multi-ethnic population of Myanmar. *BMC Evol. Biol.* **14,** 17 (2014).
26. Gazi, N. N. *et al.* Genetic Structure of Tibeto-Burman Populations of Bangladesh: Evaluating the Gene Flow along the Sides of Bay-of-Bengal. *PLoS ONE* **8,** e75064 (2013).

**Supplementary Table S7 (A total of 777 populations, 47,873 individuals)**

| Population, Region/Country | Sample Size | References |
|---|---|---|
| ***East Asia*** | | |
| Manchurian, China | 40 | 1 |
| Korean-Chinese, South Korea | 51 | 1 |
| Korean, South Korea | 185 | 1 |
| Chinese Han, Beijing, China | 40 | 1 |
| Hong Kong people, Hong Kong, China | 377 | 2 |
| Ancient workers, China | 19 | 3 |
| Japanese, Japan | 4 | 4 |
| Chinese, China | 5 | 4 |
| Tibetan, China | 168 | 5 |
| Tibetan, China | 220 | 5 |
| Tibetan, China | 71 | 5 |
| Tibetan, China | 62 | 5 |
| Tibetan, China | 61 | 6 |
| Tibetan, China | 55 | 6 |
| Tibetan, China | 59 | 6 |
| Tibetan, China | 58 | 6 |
| Tibetan, China | 46 | 6 |
| Monba, China | 51 | 6 |
| Tibetan, China | 53 | 6 |
| Lhoba, China | 20 | 6 |
| Tibetan, China | 56 | 6 |
| Tibetan, China | 59 | 6 |
| Tibetan, China | 44 | 6 |
| Tibetan, China | 40 | 7 |
| Bai, China | 40 | 8 |
| Hani, China | 33 | 8 |
| Lahu, China | 15 | 8 |
| Naxi, China | 45 | 8 |
| Pumi, China | 35 | 8 |
| Tibetan, China | 35 | 8 |
| Yi, China | 56 | 8 |
| Lahu, China | 35 | 9 |
| Lahu, China | 32 | 10 |
| Tibetan, China | 24 | 10 |
| Va, China | 36 | 9 |
| Va, China | 22 | 10 |
| Hui, China | 45 | 11 |
| Kazak, China | 53 | 11 |
| Mongoloid, China | 49 | 11 |
| Uygur, China | 47 | 11 |

| | | |
|---|---|---|
| Uzbek, China | 58 | 11 |
| Bonan, China | 95 | 12 |
| Dongxiang, China | 96 | 13 |
| Salar, China | 99 | 14 |
| Salar, China | 10 | 15 |
| Yugur, China | 100 | 16 |
| Dongxiang, China | 10 | 15 |
| Xiaohe cemetery, China | 20 | 17 |
| Xiaohe cemetery , China | 17 | 18 |
| Daheyan, Xinjiang, China | 58 | 19 |
| Niya Site, Xinjiang, China | 14 | 20 |
| Yuansha Ruins, Xinjiang, China | 15 | 20 |
| Zagunluke Cemetery, Xinjiang, China | 13 | 20 |
| Ancient samples from Xinjiang, China | 11 | 21 |
| Taiyuan, Shanxi, China | 2 | 18 |
| Daur, China | 45 | 22 |
| Ewenki, China | 47 | 22 |
| Kor, China | 48 | 22 |
| Mongoloid, China | 48 | 22 |
| Oro, China | 44 | 22 |
| Kor-S, China | 55 | 23 |
| H-Anhui, China | 42 | 24 |
| H-Fujian, China | 51 | 24 |
| H-Gansu, China | 45 | 24 |
| H-Guangxi, China | 26 | 24 |
| H-Hunan, China | 16 | 24 |
| H-Jiangsu, China | 67 | 24 |
| H-Jiangxi, China | 23 | 24 |
| H-Liaoning, China | 51 | 24 |
| H-Neimeng, China | 45 | 24 |
| H-Qinhai, China | 44 | 24 |
| H-Shanghai, China | 56 | 24 |
| H-Shannxi, China | 53 | 24 |
| H-Sichuan, China | 70 | 24 |
| H-Yunnan, China | 58 | 24 |
| H-Zhejiang, China | 61 | 24 |
| H-GD, China | 30 | 25 |
| H-LN, China | 51 | 25 |
| H-QD, China | 50 | 25 |
| H-WH, China | 42 | 25 |
| H-XJ, China | 47 | 25 |
| H-YN, China | 43 | 25 |
| H-SD, China | 76 | 26 |
| Han, Yan Bian, Jilin, China | 51 | 23 |

| | | |
|---|---|---|
| Ab-Taiwanese, China | 180 | 27 |
| TW-Han, China | 155 | 28 |
| H-GD, China | 69 | 29 |
| C-SH, China | 120 | 30 |
| H-CS, China | 82 | 31 |
| H-Xi'an, China | 85 | 31 |
| Aini, China | 47 | 8 |
| BAI, China | 19 | 8 |
| Jino, China | 18 | 8 |
| QH-Tib, China | 56 | 8 |
| Tujia, China | 66 | 8 |
| Tujia, China | 31 | 8 |
| YN-Dai, China | 21 | 10 |
| YN-Dai, China | 38 | 7 |
| MHN, China | 103 | 32 |
| MYN, China | 39 | 32 |
| YBN, China | 19 | 32 |
| YBP, China | 35 | 32 |
| YDB, China | 10 | 32 |
| YGS, China | 24 | 32 |
| YHT, China | 19 | 32 |
| YKM, China | 40 | 32 |
| YLO, China | 42 | 32 |
| YLT, China | 26 | 32 |
| YMB, China | 6 | 32 |
| YMI, China | 32 | 32 |
| YPA, China | 32 | 32 |
| YTU, China | 41 | 32 |
| YWU, China | 31 | 32 |
| YXB, China | 11 | 32 |
| YYM, China | 27 | 32 |
| Q_Tib, China | 76 | 5 |
| G_Tib, China | 83 | 5 |
| XZ-Lhoba, China | 50 | Our unpublished data |
| XZ-Monba, China | 31 | Our unpublished data |
| wanyan, China | 46 | Our unpublished data |
| Japanese, Japan | 50 | 33 |
| Japanese, Japan | 162 | 34 |
| Japanese, Japan | 211 | 35 |
| Gifu, Japan | 137 | 36 |
| Japanese, Japan | 150 | 30 |
| Japanese, Japan | 100 | 37 |
| Japanese, Japan | 124 | 38 |
| Japanese, Japan | 62 | 39 |

| | | |
|---|---|---|
| Ainu, Japan | 50 | 39 |
| Pyukyuan, Japan | 50 | 39 |
| Achang-YN, China | 60 | Our unpublished data |
| Bai-YN, China | 21 | Our unpublished data |
| Bai-YN, China | 19 | Our unpublished data |
| Blang-YN, China | 90 | Our unpublished data |
| Dai-YN, China | 46 | Our unpublished data |
| Dai-YN, China | 24 | Our unpublished data |
| Dai-YN, China | 30 | Our unpublished data |
| Dai-YN, China | 27 | Our unpublished data |
| Deang-YN, China | 52 | Our unpublished data |
| Drung -YN, China | 35 | Our unpublished data |
| Hani-YN, China | 9 | Our unpublished data |
| Hani-YN, China | 39 | Our unpublished data |
| Hani-YN, China | 18 | Our unpublished data |
| Jingpo-YN, China | 60 | Our unpublished data |
| Jino-YN, China | 51 | Our unpublished data |
| Lahu-YN, China | 26 | Our unpublished data |
| Lahu-YN, China | 36 | Our unpublished data |
| Lisu-YN, China | 31 | Our unpublished data |
| Lisu-YN, China | 20 | Our unpublished data |
| Lisu-YN, China | 18 | Our unpublished data |
| Naxi-YN, China | 62 | Our unpublished data |
| Naxi-YN, China | 6 | Our unpublished data |
| Nu-YN, China | 40 | Our unpublished data |
| Pumi-YN, China | 19 | Our unpublished data |
| Pumi-YN, China | 25 | Our unpublished data |
| Tibetan-YN, China | 11 | Our unpublished data |
| Tibetan-YN, China | 54 | Our unpublished data |
| Wa-YN, China | 14 | Our unpublished data |
| Yao-YN, China | 8 | Our unpublished data |
| Yi-YN, China | 28 | Our unpublished data |
| Yi-YN, China | 67 | Our unpublished data |
| Blang-YN, China | 48 | Our unpublished data |
| DL-YN, China | 48 | Our unpublished data |
| Dongxiang-GS, China | 63 | Our unpublished data |
| GL-GZ, China | 102 | Our unpublished data |
| GX-Dong, China | 100 | Our unpublished data |
| GX-Maonan, China | 48 | Our unpublished data |
| GX-Mulam, China | 48 | Our unpublished data |
| GX-Yao, China | 29 | Our unpublished data |
| GZ-Bouyei, China | 85 | Our unpublished data |
| GZ-Miao, China | 35 | Our unpublished data |
| GZ-She, China | 49 | Our unpublished data |

| | | |
|---|---|---|
| GZ-Shui, China | 64 | Our unpublished data |
| GZ-Yao, China | 40 | Our unpublished data |
| Han-GS, China | 43 | Our unpublished data |
| Han-JS, China | 1 | Our unpublished data |
| Han-NX, China | 111 | Our unpublished data |
| Han-SX, China | 2 | Our unpublished data |
| Hainan-Li, China | 59 | Our unpublished data |
| HN-Miao, China | 49 | Our unpublished data |
| HN-Tujia, China | 46 | Our unpublished data |
| HN-Yao, China | 52 | Our unpublished data |
| Hui-QH, China | 33 | Our unpublished data |
| Hui-GS, China | 21 | Our unpublished data |
| IM-Buryat, China | 59 | Our unpublished data |
| IM-Man, China | 122 | Our unpublished data |
| IM-Xibe, China | 49 | Our unpublished data |
| Li-HI, China | 216 | Our unpublished data |
| LN-Man, China | 31 | Our unpublished data |
| Han-BJ, China | 45 | Our unpublished data |
| Han-QH, China | 142 | Our unpublished data |
| QH-Tu, China | 64 | Our unpublished data |
| QJ-YN, China | 40 | Our unpublished data |
| Sala-QH, China | 16 | Our unpublished data |
| XM, China | 40 | Our unpublished data |
| YN-Bai, China | 66 | Our unpublished data |
| YN-Blang, China | 37 | Our unpublished data |
| YN-Dai, China | 85 | Our unpublished data |
| YN-Hani, China | 44 | Our unpublished data |
| YN-Jino, China | 31 | Our unpublished data |
| YN-Kucong, China | 34 | Our unpublished data |
| YN-Lisu, China | 43 | Our unpublished data |
| YN-Mosuo, China | 64 | Our unpublished data |
| YN-Naxi, China | 56 | Our unpublished data |
| YN-Pumi, China | 59 | Our unpublished data |
| YN-Yi, China | 90 | Our unpublished data |
| Zhuang-GX, China | 79 | Our unpublished data |
| Han-GD, China | 105 | 40 |
| HHT-NM, China | 107 | 41 |
| LQ-NM, China | 48 | 41 |
| TH-YN, China | 46 | 41 |
| AC, China | 6 | 42 |
| BG, China | 32 | 42 |
| BU, China | 31 | 42 |
| CL, China | 30 | 42 |
| CU, China | 30 | 42 |

| | | |
|---|---|---|
| CX, China | 25 | 42 |
| CY, China | 12 | 42 |
| DA, China | 56 | 42 |
| DG, China | 40 | 42 |
| DN, China | 10 | 42 |
| GA, China | 42 | 42 |
| HL, China | 34 | 42 |
| HS, China | 30 | 42 |
| JM, China | 27 | 42 |
| LC, China | 30 | 42 |
| LG, China | 31 | 42 |
| LL, China | 4 | 42 |
| LQ, China | 25 | 42 |
| MK, China | 33 | 42 |
| ML, China | 39 | 42 |
| MN, China | 32 | 42 |
| MO, China | 29 | 42 |
| MQ, China | 17 | 42 |
| PO, China | 34 | 42 |
| PY, China | 30 | 42 |
| RG, China | 31 | 42 |
| TN, China | 30 | 42 |
| WG, China | 14 | 42 |
| WS, China | 33 | 42 |
| YR, China | 15 | 42 |
| HAN- Chaoshan, GD, China | 102 | 43 |
| HAN- Meizhou, GD, China | 170 | 43 |
| Northern-Han, China | 60 | 44 |
| Japanese_Honshu, Japan | 82 | 44 |
| Indonesian, Indonesian | 54 | 44 |
| Koryak-KMAN, Koryak | 110 | 44 |
| Japanese_Kyushu, Japan | 104 | 44 |
| Malaysian-MAL, Malaysian | 52 | 44 |
| Nivkhi, Nivkhi | 57 | 44 |
| Okinawa, Japan | 45 | 44 |
| Philippine, Philippine | 29 | 44 |
| Philippine, Philippine | 30 | 44 |
| Kam, China | 72 | 45 |
| Laka, China | 67 | 45 |
| Mien, China | 29 | 45 |
| Mulam, China | 27 | 45 |
| Pinghua_Han, China | 39 | 45 |
| Pinghua_Han, China | 48 | 45 |
| Pinghua_Han, China | 111 | 45 |

| | | |
|---|---|---|
| Zhuang_GB, China | 54 | 45 |
| Zhuang_GB, China | 4 | 45 |
| Zhuang_GB, China | 9 | 45 |
| Zhuang_GB, China | 10 | 45 |
| Bunun, China | 18 | 46 |
| Atayal, China | 18 | 46 |
| Amis, China | 21 | 46 |
| Paiwan, China | 21 | 46 |
| Undefined, Taiwan China | 64 | 47 |
| Han-JS, China | 99 | Our unpublished data |
| HSK-XJ, China | 44 | Our unpublished data |
| Kirgiz-XJ, China | 60 | Our unpublished data |
| Mg-XJ, China | 96 | Our unpublished data |
| Tajik-XJ, China | 56 | Our unpublished data |
| Uyu-XJ, China | 274 | Our unpublished data |
| Uzbek-XJ, China | 46 | Our unpublished data |
| Han-HLJ, China | 180 | Our unpublished data |
| Han-SX, China | 220 | Our unpublished data |
| Han-JL, China | 199 | Our unpublished data |
| Han-LN, China | 191 | Our unpublished data |
| Han-HN, China | 1502 | 48 |
| Han-YN, China | 396 | Our unpublished data |
| Han-SN, China | 398 | Our unpublished data |
| Han-AH, China | 375 | Our unpublished data |
| Han cancer of the esophagus, China | 190 | 49 |
| Han cancer of the esophagus, China | 106 | Our unpublished data |
| Han-SC, China | 556 | Our unpublished data |
| Han-SC, China | 214 | Our unpublished data |
| Han-SC, China | 312 | Our unpublished data |
| Koreans 103, South Korea | 103 | 50 |
| ZA_Chamdo_Ji, China | 29 | 51 |
| ZA_Lhasa_Ji, China | 44 | 51 |
| ZA_Nyingchi_Ji, China | 52 | 51 |
| ZA_Shigatse_Ji, China | 29 | 51 |
| ZA_Shannan_Ji, China | 55 | 51 |
| ZA_Nakchu_Ji, China | 5 | 51 |
| Tib_Ji, China | 73 | 51 |
| Tibetan, China | 156 | 52 |
| Korean, South Korea | 694 | 53 |
| HA, Shandong, China | 253 | Our unpublished data |
| HA, Neimeng, China | 141 | Our unpublished data |
| Dongbei, China | 249 | Our unpublished data |
| QI, Sichuan, China | 60 | Our unpublished data |
| ***Southeast Asia*** | | |

| | | |
|---|---|---|
| Vietnamese, Vietnam | 42 | 1 |
| Thais, Thailand | 40 | 1 |
| Vietnamese, Vietnam | 187 | 54 |
| Burman, Karen, Myanmar | 327 | 55 |
| Thai LHON, Thailand | 42 | Tharaphan et al. GenBank DQ149024-DQ149065 |
| Indonesian, Sumatra, Pekanbaru, Indonesia | 55 | 56 |
| Indonesian, Sumatra, Medan, Indonesia | 44 | 56 |
| Malaysian, Semang, Malaysia | 263 | 56 |
| Malaysian, Melayu, Malaysia | 4 | 56 |
| Vietnamese, Vietnam | 2 | 4 |
| Samoan, Samoa | 9 | 4 |
| Philippines, Philippines | 3 | 4 |
| Borneo, Borneo | 13 | 4 |
| Java, Java | 2 | 4 |
| Burmans, Myanmar | 32 | This study |
| Burmans, Myanmar | 54 | This study |
| Burmans, Myanmar | 51 | This study |
| Burmans, Myanmar | 122 | This study |
| Burmans, Myanmar | 69 | This study |
| Burmans, Myanmar | 72 | This study |
| Chin, Myanmar | 58 | This study |
| Chin, Myanmar | 13 | This study |
| Chin, Myanmar | 187 | This study |
| Naga, Myanmar | 30 | This study |
| Naga, Myanmar | 39 | This study |
| Naga, Myanmar | 32 | This study |
| Rakhine, Myanmar | 24 | This study |
| Rakhine, Myanmar | 63 | This study |
| Akha, Thailand | 91 | 57 |
| Lahu, Thailand | 39 | 57 |
| Lisu, Thailand | 25 | 57 |
| Lisu, Thailand | 42 | 57 |
| Lisu, Thailand | 53 | 57 |
| Mussur, Thailand | 21 | 57 |
| Cham, Vietnam | 168 | 58 |
| Kinh, Vietnam | 139 | 58 |
| Thai, Thailand | 190 | 59 |
| Thai, Thailand | 32 | 7 |
| Cambodia, Cambodia | 31 | 60 |
| Thai, Thailand | 30 | 61 |
| Thai, Thailand | 44 | 61 |

| | | |
|---|---|---|
| Lao Song, Thailand | 25 | 61 |
| ChB, Thailand | 20 | 62 |
| Khm, Thailand | 22 | 62 |
| Th_K, Thailand | 32 | 62 |
| Phuthai, Thailand | 25 | 61 |
| Chong, Thailand | 25 | 61 |
| Bangka, Sumatra, Indonesia | 34 | 63 |
| Batek, Malaysia | 45 | 63 |
| Jahai, Malaysia | 39 | 63 |
| Medan, Sumatra, Indonesia | 42 | 63 |
| Melayu, Malaysia | 6 | 63 |
| Mendriq, Malaysia | 31 | 63 |
| Padang, Malaysia | 24 | 63 |
| Palembang, Sumatra, Indonesia | 50 | 63 |
| Pekanbaru, Sumatra, Indonesia | 52 | 63 |
| Semai, Malaysia | 6 | 63 |
| Semelai, Malaysia | 61 | 63 |
| Temiar, Malaysia | 46 | 63 |
| Temuan, Malaysia | 33 | 63 |
| Philippine, Philippines | 61 | 46 |
| Java, Indonesia | 46 | 46 |
| Banjarmasin, Indonesia | 89 | 46 |
| Kota Kinabalu, Malaysia | 61 | 46 |
| Manado, Indonesia | 89 | 46 |
| Palu, Indonesia | 38 | 46 |
| Ujung Padang, Indonesia | 46 | 46 |
| Toraja, Indonesia | 64 | 46 |
| Bali, Indonesia | 82 | 46 |
| Mataram, Indonesia | 44 | 46 |
| Flores, Indonesia | 2 | 46 |
| Waingapu, Indonesia | 50 | 46 |
| Alor, Malaysia | 45 | 46 |
| Ambon, Indonesia | 43 | 46 |
| Luzon, Philippines | 168 | 47 |
| Visayas, Philippines | 103 | 47 |
| Mindanao, Philippines | 70 | 47 |
| Mal, Malaysia | 124 | 64 |
| HA, Singapore | 205 | 65 |
| Sakai, Thailand | 20 | 61 |
| Mussur, Thailand | 21 | 61 |
| White Karen, Thailand | 40 | 57 |
| Red Karen, Thailand | 39 | 57 |
| BK, Thailand | 13 | 62 |
| NUL, Thailand | 17 | 62 |

| | | |
|---|---|---|
| Benu, Malaysia | 10 | 66 |
| Gopeng, Malaysia | 22 | 66 |
| Kuala Kurau, Malaysia | 17 | 66 |
| Lembah Bujang, Malaysia | 26 | 66 |
| Lenggeng, Malaysia | 12 | 66 |
| Machang, Malaysia | 24 | 66 |
| Kota Bharu, Malaysia | 5 | 66 |
| Muar, Malaysia | 17 | 66 |
| Parit Buntar, Malaysia | 16 | 66 |
| Parit Buntar, Malaysia | 1 | 66 |
| Pontian, Malaysia | 20 | 66 |
| Rantau Panjang, Malaysia | 32 | 66 |
| Semerah, Malaysia | 13 | 66 |
| Sri Menanti, Malaysia | 22 | 66 |
| Yan, Malaysia | 11 | 66 |
| Laos, Laos | 214 | 67 |
| ***South Asia*** | | |
| Tipperah, India | 20 | 68 |
| Indians, Jammu and Kashmir, India | 7 | Bhat et al. GenBank AN. AY642034-AY642040 |
| Indian, India | 12 | Darvishi et al. GenBank DQ143184-DQ143195 |
| Indian, north India | 4 | Darvishi et al. GenBank DQ176757-DQ176760 |
| Chakma, India | 92 | Our unpublished data |
| Ralt, India | 104 | Our unpublished data |
| Kuki, India | 45 | Our unpublished data |
| Adi, India | 45 | 69 |
| Apatani, India | 26 | 69 |
| Apatani, India | 21 | 69 |
| Naga, India | 43 | 69 |
| Nishi, India | 44 | 69 |
| Garo, India | 76 | 70 |
| Lyngnga, India | 74 | 70 |
| Nongtrai, India | 27 | 70 |
| Maram, India | 60 | 70 |
| Bhoi, India | 29 | 70 |
| Khynriam, India | 82 | 70 |
| War_Khas, India | 29 | 70 |
| Pnar, India | 51 | 70 |
| War_Jaint, India | 17 | 70 |
| Pakistani, Pakistan | 100 | 71 |
| Baluch, Pakistan | 39 | 71 |
| Brahui, Pakistan | 38 | 71 |

| | | |
|---|---|---|
| Gujarati, India | 34 | 71 |
| Hazara, Pakistan | 23 | 71 |
| Hunza Burusho, Pakistan | 44 | 71 |
| Kalash, Pakistan | 44 | 71 |
| Makrani, Pakistan | 33 | 71 |
| Mazandarian, Pakistan | 44 | 71 |
| Pathan, Pakistan | 44 | 71 |
| Sindhi, Pakistan | 23 | 71 |
| Moor, Sri Lanka | 43 | 72 |
| Sinhalese, Sri Lanka | 59 | 72 |
| Indo-European, India | 94 | 73 |
| Dravidian, India | 17 | 73 |
| Kadar, India | 7 | 74 |
| Mixed MD, MDU, TH, India | 96 | Our unpublished data |
| Mixed DG, AMK, ARA, CNP, ERI, KAM, India | 400 | Our unpublished data |
| Mixed KAN, KAS, NAT, TNP, ODC, India | 263 | Our unpublished data |
| Mixed NGL, VRU,VEM, VKD, TN, MX, India | 190 | Our unpublished data |
| BadugaBad, India | 2 | 75 |
| Irula, India | 16 | 68, 75 |
| Kota, India | 25 | 69 |
| Kurumba, India | 8 | 69 |
| Kurumba Betta, India | 19 | 69 |
| Kurumba Mullu, Mulla Krumba, India | 15 | 69 |
| OoraliOo, India | 3 | 75 |
| SakkiliSak, India | 6 | 75 |
| Soligas, India | 12 | 69 |
| Malayalam , India | 3 | 76 |
| Cochin, India | 52 | 72 |
| Cochin Jews, India | 37 | 72 |
| Kattunaiken, India | 16 | 69 |
| Kuruchian, India | 46 | 69 |
| Kuruman Mullu, Mullu Kurunan, India | 37 | 69 |
| Paniya, India | 11 | 69 |
| Mixed TVM, India | 64 | Our unpublished data |
| Havik, India | 38 | 74 |
| Mukri, India | 42 | 74 |
| KannadaKA, India | 5 | 76 |
| Koragas, India | 31 | 69 |
| Kuruva, India | 25 | 77 |
| Kurumba Jenu, India | 6 | 69 |
| Yerava, India | 53 | 69 |

| | | |
|---|---|---|
| Bhovi, India | 30 | 78 |
| Gowda, India | 37 | 78 |
| Brahmin, India | 29 | 78 |
| Lingayat, India | 17 | 78 |
| Christians, India | 21 | 78 |
| Muslim, India | 24 | 78 |
| Siddi, India | 7 | 79 |
| TeluguAndhra Pradesh, Ind, TE, India | 15 | 76, 80, 81 |
| Lambadi, India | 75 | 80 |
| Andh, AndhSA, AndhX, India | 95 | 69, 82 |
| BrahminBV, BN, India | 34 | 80 |
| KapuKT, KTK, KP, India | 67 | 80, 83 |
| Madiga, India | 24 | 80 |
| Yadava, India | 40 | 80 |
| Wadabahija, India | 8 | 80 |
| Mala, India | 23 | 80 |
| Kshatriya, India | 9 | 80 |
| Relli, India | 20 | 80 |
| Jalari, India | 7 | 80 |
| Vysya, India | 9 | 80 |
| Chenchu, India | 96 | 84 |
| Koya, India | 81 | 84 |
| ErukulaEr, India | 5 | 75 |
| YanadiYn, India | 10 | 75 |
| ThogataveeraT, India | 84 | Our unpublished data |
| ReddyR, India | 68 | Our unpublished data |
| Pardhi, India | 38 | 69 |
| Thoti, India | 39 | 69 |
| AkhutotaAK, India | 22 | 83 |
| PantaWatkins, et al., India | 29 | 83 |
| PokanatiPO, India | 36 | 83 |
| VanneVN, India | 26 | 83 |
| Irula, India | 19 | 85 |
| Chenchu, India | 75 | 86 |
| Kolam, India | 128 | 86 |
| Gond, India | 75 | 86 |
| NaikpodN, NP, Z, India | 83 | 82 |
| PardhanD, I, J, S, W, India | 170 | 82 |
| Chitpavan Brahmin, India | 17 | 87 |
| Desasth Brahmin, India | 15 | 87 |
| BrahminKBR, India | 9 | 88 |
| Konkanastha Brahmin, India | 50 | 72 |
| Dhangar, India | 16 | 87 |
| Maratha, India | 30 | 87, 88, 89 |

| | | |
|---|---|---|
| Maharashtra, MarathiMA, Mah, India | 29 | 76, 80 |
| Nav-Baudh, India | 17 | 88, 89 |
| Parsi, India | 37 | 72 |
| Bohra, India | 5 | 89 |
| Irani, India | 5 | 89 |
| Korku, India | 9 | 89 |
| Madia Gond, India | 13 | 89 |
| Kolam, India | 8 | 89 |
| KathodiKa, India | 1 | 75 |
| KoliKo, India | 6 | 75 |
| Sikh, India | 23 | 69 |
| GujaratGuj, GJ, India | 61 | 71, 72 |
| Brahmin, India | 29 | 90 |
| Khatri, India | 11 | 90 |
| Jat Singh, India | 27 | 72, 90 |
| Scheduled caste, India | 19 | 72, 90 |
| Lobana, India | 49 | 80 |
| BrahminPuj, India | 18 | 72 |
| Kshatriya, India | 22 | 72 |
| PunjabP, PBAN, PHC, PNIR, PP, PPUR, PPUS, PRAJ, PRAM, PSUR, PSUS, PUSH , India | 42 | 73, 81 |
| Bhoksa, India | 22 | 80 |
| Tharu, India | 31 | 72, 80 |
| MundaMun, India | 4 | 75 |
| KoriKor, India | 1 | 75 |
| RohidasRo, India | 1 | 75 |
| YadavaYd, India | 6 | 75 |
| BhargavaA, India | 165 | Our unpublished data |
| ChaturvediB, India | 82 | Our unpublished data |
| Brahmin MixedC, India | 146 | Our unpublished data |
| Uttar Pradesh Brahmin, India | 23 | 72 |
| RajputRj, India | 14 | 75 |
| LodheLo, India | 1 | 75 |
| HI, U, Uttar Pradesh, USIN, UKIR, UANI, UBHA, UBOB, UCHA, UD, UHC, UJEA, India | 93 | 73, 76, 80 |
| Delhi, India | 10 | 81 |
| Kanet, India | 29 | 72 |
| Rajput, Rajsthan, India | 33 | 72, 80 |
| Kashmir, India | 13 | 80 |
| Baluch, Pakistan | 14 | 71 |
| Brahui, Pakistan | 12 | 71 |
| Hazara, Pakistan | 12 | 71 |

| | | |
|---|---|---|
| Hunza Burusho, Pakistan | 23 | 71 |
| KarachiKAR, Pakistan, UR, Pakistan | 74 | 71, 76, 80 |
| Parsi, Pakistan | 23 | 71 |
| Pathan, Pakistan | 23 | 71 |
| SindhiSI, Pakistan | 14 | 71 |
| Pushtoons, Pakistan | 16 | 69 |
| Makrani, Pakistan | 8 | 69 |
| BI, BD, BHC, BSUY, BVIJ, Bihar, India | 43 | 73, 76, 80 |
| BhumijBhuJ, BM, India | 60 | 90, 91 |
| Ho, India | 20 | 91 |
| KhariaKhaJ, KHA, India | 39 | 83, 91 |
| KanwarKan, India | 6 | 75 |
| SatnamiSat, India | 3 | 75 |
| SanthalSa, SA, India | 45 | 75, 83 |
| AsurAS, India | 30 | 83 |
| MundaMU, India | 23 | 83 |
| BirhorBir, India | 189 | Our unpublished data |
| MundaMun, India | 169 | Our unpublished data |
| OranOR, India | 169 | Our unpublished data |
| PhariyaPha, India | 160 | Our unpublished data |
| BhariaBh, India | 14 | 75 |
| GondGo, Madia Gond, India | 4 | 75, 85 |
| OranOr, India | 16 | 75 |
| Muria, India | 12 | 68 |
| Brahmin, India | 16 | 92 |
| Gope, India | 17 | 92 |
| Juang, India | 20 | 92 |
| Karan, India | 14 | 92 |
| Khandayat, India | 14 | 92 |
| Paroja, India | 21 | 92 |
| Saora, India | 17 | 92 |
| Orisa, India | 2 | 80 |
| BE, Ben, Bengal, India | 15 | 75, 76, 80 |
| Bengal Brahmin, India | 36 | 72 |
| Bengal Sudra, India | 8 | 72 |
| RajbhansiSW, Ra, RJ, India | 249 | Our unpublished data |
| Kurmi, India | 55 | 72 |
| Lodha, Lodhar, India | 68 | 68, 72 |
| Munda, India | 6 | 68 |
| Santhal, India | 14 | 68 |
| DhimalDH, India | 75 | Our unpublished data |
| KolKL, India | 73 | Our unpublished data |
| LachungLA, India | 35 | Our unpublished data |

| | | |
|---|---|---|
| MechME, India | 45 | Our unpublished data |
| MundaMun, India | 40 | Our unpublished data |
| OranOR, India | 98 | Our unpublished data |
| PhariyaPha, India | 175 | Our unpublished data |
| RabhaRV, India | 49 | Our unpublished data |
| SanthalSA, India | 83 | Our unpublished data |
| TotoTo, India | 11 | Our unpublished data |
| MixedSc, MX, WBX, India | 409 | Our unpublished data |
| BodoBO, India | 65 | Our unpublished data |
| MuslimM, India | 71 | Our unpublished data |
| Bangladesh, Bangladesh | 1 | 80 |
| BangS, Bangladesh | 27 | 69 |
| MixedBN, Bangladesh | 143 | Our unpublished data |
| Great Andamanese, India | 20 | 93 |
| Jarawa, India | 4 | 93 |
| Onge, India | 62 | 93 |
| Nicobarese, India | 46 | 93, 94 |
| Ao, India | 19 | 75 |
| Mixed BH, Bhutan | 47 | Our unpublished data |
| Mixed N, Nepal | 61 | Our unpublished data |
| Ladakhis-Bud, Kashmir | 23 | 95 |
| Ladakhis-Mus, Kashmir | 9 | 95 |
| Shia Muslim, India | 120 | 96 |
| Sunni Muslim, India | 131 | 96 |
| Dawoodi BohraTN, India | 62 | 96 |
| Dawoodi BohraGUJ, India | 50 | 96 |
| Mappla, India | 61 | 96 |
| Iranian Shia, India | 48 | 96 |
| Tharu-C I, Nepal | 57 | 97 |
| Tharu-C II, Nepal | 76 | 97 |
| Tharu-E, Nepal | 40 | 97 |
| Nepal-Kathamandu, Nepal | 200 | Our unpublished data |
| Nepal-East Nepal, Nepal | 46 | Our unpublished data |
| Tamang, Nepal | 46 | 52 |
| Newar, Nepal | 67 | 52 |
| Kathmandu, Nepal | 77 | 52 |
| ***North Asia*** | | |
| Besermyans, Udmurtia | 41 | 98 |
| Mongolian, Mongolia | 47 | 1 |
| Volga-Turkic, Russia | 2 | Zaporozhchenko et al. GenBank AN. DQ656492-DQ656493 |
| Aznakaevo, Aznakaevo | 71 | 99 |
| Buinsk, Buinsk | 125 | 99 |
| Mongoloid, Mongolia | 103 | 100 |

| | | |
|---|---|---|
| AEG, Mongolia | 39 | 101 |
| PDEG, Mongolia | 124 | 101 |
| PDM, Mongolia | 104 | 101 |
| PDY, Mongolia | 44 | 101 |
| Mongoloid, Mongolian | 46 | 101 |
| Buryat, South Siberia | 40 | 102 |
| Tuvinian, South Siberia | 36 | 102 |
| Russian, north-eastern Siberia | 50 | 103 |
| Ukrainian, north-eastern Siberia | 18 | 103 |
| Altaian, South Siberia | 110 | 104 |
| Khakassian, South Siberia | 53 | 104 |
| Buryat, South Siberia | 91 | 104 |
| Sojot, South Siberia | 30 | 104 |
| Todjin, South Siberia | 48 | 104 |
| Tuvinian, South Siberia | 90 | 104 |
| Tofalar, South Siberia | 58 | 104 |
| Buryat, Ulan-Ude | 126 | 105 |
| Yakut, Yakutsk | 117 | 105 |
| Yakut, northeastern Siberia | 191 | 106 |
| Even, Russia | 65 | 107 |
| Koryak, Russia | 35 | 107 |
| Yakutian, Russia | 22 | 107 |
| Kets, Sulomai and Turukhansk | 38 | 108 |
| Ngana-sans, Dudinka | 24 | 108 |
| Ba, bashkir | 221 | 109 |
| Ch, chuvash | 55 | 109 |
| Er, erza-moksha | 102 | 109 |
| Ko, komi | 138 | 109 |
| Ma, mari | 139 | 109 |
| Ta, tatar | 228 | 109 |
| Ud, udmurt | 102 | 109 |
| TB, Southern Siberia | 72 | 110 |
| TV, Southern Siberia | 96 | 110 |
| BR, Southern Siberia | 25 | 110 |
| TF, Southern Siberia | 46 | 110 |
| EV, Southern Siberia | 37 | 110 |
| NG, Southern Siberia | 33 | 110 |
| UL, Southern Siberia | 87 | 110 |
| NV, Southern Siberia | 56 | 110 |
| UD, Southern Siberia | 46 | 110 |
| Mansi, Northwest Siberia | 98 | 108 |
| Nga, Arctic Siberians | 39 | 111 |
| Ind, Arctic Siberians | 82 | 111 |
| Kolyma, Arctic Siberians | 18 | 111 |

| | | |
|---|---|---|
| Chv, Arctic Siberians | 32 | 111 |
| Chu, Arctic Siberians | 182 | 111 |
| Sir, Arctic Siberians | 37 | 111 |
| Cha, Arctic Siberians | 50 | 111 |
| Nau, Arctic Siberians | 39 | 111 |
| Ale, Arctic Siberians | 36 | 111 |
| Buryat, Russia | 61 | 44 |
| Even, Russia | 35 | 44 |
| Chelkan, Northern Altaians | 91 | 112 |
| Kumandin, Northern Altaians | 52 | 112 |
| Tubalar, Northern Altaians | 71 | 112 |
| Altai-kizhi , Southern Altaians | 276 | 112 |
| Barghuts, Hulun Buir Aimak ,Inner Mongolia, China | 149 | 113 |
| Altaian Kazakhs, Kosh-Agach district of Altai Republic | 98 | 113 |
| Mongolians, Ulaanbaatar, Mongolia | 47 | 50 |
| Kalmyks, Kalmyk Republic | 110 | 50 |
| Buryats, Buryat Republic | 295 | 50 |
| Khamnigans, Buryat Republic | 99 | 50 |
| Tuvinians, Tuva Republic | 105 | 50 |
| East Evenks, Buryat Republic | 45 | 50 |
| West Evenks, Krasnoyarsk | 73 | 50 |
| Yakuts, Sakha | 36 | 50 |
| Shors, Kemerovo | 82 | 50 |
| Khakassians, Khakassian Republic | 57 | 50 |
| Altaians-Kizhi, South Altai | 90 | 50 |
| Telenghits, South Altai | 71 | 50 |
| Teleuts, Kemerovo | 53 | 50 |
| Chukchi, Anadyr, Chukotka Autonomous Okrug | 15 | 50 |
| Khanty, Lower Ob-river valley | 106 | 114 |
| Mansi, Lower Ob-river valley | 63 | 114 |
| Complete sequences of C and D, North Asia | 182 | 115 |
| Complete sequences of North Asia rare haplogroups | 55 | 113 |
| Tubalar, Altai region | 144 | 116 |
| Evens, northeastern Siberia | 87 | 116 |
| Ulchi, Russian Far East | 160 | 116 |
| ***Central Asia*** | | |
| Bukharan Arabs, Central Asia | 20 | 117 |
| Crimean Tatars, Central Asia | 20 | 117 |
| Dungans, Central Asia | 16 | 117 |

| | | |
|---|---|---|
| Iranians, Central Asia | 20 | 117 |
| Karakalpaks, Central Asia | 20 | 117 |
| Kazaks, Central Asia | 20 | 117 |
| Khoremian Uzbeks, Central Asia | 20 | 117 |
| Kyrgyz, Central Asia | 20 | 117 |
| Tajiks, Central Asia | 20 | 117 |
| Turkmen, Central Asia | 20 | 117 |
| Uighurs, Central Asia | 16 | 117 |
| Uzbeks, Central Asia | 20 | 117 |
| Kazakh, Kazakhstan | 55 | 117 |
| Kirghiz, Kirghizstan | 47 | 117 |
| Kirghiz, Kirghizstan | 48 | 117 |
| Uighur, Kazakhstan | 55 | 117 |
| Kurdish, Turkmenistan | 32 | 71 |
| Shugnan, Tajikistan | 44 | 71 |
| Turkmen, Turkmenistan | 41 | 71 |
| Uzbek, Uzbekistan | 42 | 71 |
| FER, Uzbekistan | 53 | 118 |
| KAR, Uzbekistan | 46 | 118 |
| KAZ, Kazakhstan | 256 | 118 |
| KYR, Kyrgyzstan | 249 | 118 |
| QAS, Uzbekistan | 75 | 118 |
| RUS, Russia | 151 | 118 |
| TAJ, Tajikistan | 244 | 118 |
| TAS, Uzbekistan | 55 | 118 |
| TUR, Turkmenistan | 249 | 118 |
| XOR, Uzbekistan | 99 | 118 |
| KAR, Uzbekistan | 55 | 119 |
| KAZ, Kazakhstan | 50 | 119 |
| OTU, Uzbekistan/Turkmenistan border | 53 | 119 |
| TUR, Uzbekistan/Turkmenistan border | 51 | 119 |
| UZB, Karakalpakia | 40 | 119 |
| Kazakhstan, Kazakhstan | 27 | 120 |
| Tajiks, Tajikistan | 44 | 50 |
| ***West Asia*** | | |
| Kurdish Muslims, Baghdad, Iraq | 15 | 121 |
| Assyrian Christians, Baghdad, Iraq | 22 | 121 |
| Arab Muslims, Baghdad, Iraq | 128 | 121 |
| Mandaean Arabs, Baghdad, Iraq | 17 | 121 |
| Kuwaiti, Kuwait | 381 | 122 |
| Yemeni, Yemen | 50 | 123 |
| Bedouin, Near East | 58 | 124 |
| Cherkes, Near East | 8 | 124 |
| Druze, Near East | 77 | 124 |

| | | |
|---|---|---|
| Palestinian, Near East | 110 | 124 |
| Turkish, Turkey | 50 | 71 |
| BAL, Georgia | 20 | 71 |
| CHE, Georgia | 18 | 71 |
| GEO, Georgia | 20 | 71 |
| Gilaki, Iran | 37 | 71 |
| Kurdish, Iran | 20 | 71 |
| Lur, Iran | 17 | 71 |
| Mazandarian, Iran | 21 | 71 |
| Persian, Iran | 42 | 71 |
| Turkish, Azerbaijan | 40 | 71 |
| AFG, Afghanistan | 98 | 118 |
| Persians, eastern Iran | 82 | 50 |
| Kurds, northwestern Iran | 25 | 50 |

**References**


1. Jin H-J, Tyler-Smith C, Kim W. The peopling of Korea revealed by analyses of mitochondrial DNA and Y-chromosomal markers. *PLoS ONE* **4**, e4210 (2009).
2. Irwin JA, *et al.* Investigation of heteroplasmy in the human mitochondrial DNA control region: a synthesis of observations from more than 5000 global population samples. *J. Mol. Evol.* **68**, 516-527 (2009).
3. Xu Z, *et al.* Mitochondrial DNA evidence for a diversified origin of workers building mausoleum for first emperor of China. . *PLoS ONE* **3**, e3275 (2008).
4. Koji Lum J, Cann RL. mtDNA lineage analyses: origins and migrations of Micronesians and Polynesians. *Am. J. Phys. Anthropol.* **113**, 151-168 (2000).
5. Zhao M, *et al.* Mitochondrial genome evidence reveals successful Late Paleolithic settlement on the Tibetan Plateau. *Proc. Natl. Acad. Sci.* **106**, 21230-21235 (2009).
6. Qin Z, *et al.* A mitochondrial revelation of early human migrations to the Tibetan Plateau before and after the last glacial maximum. *Am. J. Phys. Anthropol.* **143**, 555-569 (2010).
7. Yao Y-G, Nie L, Harpending H, Fu Y-X, Yuan Z-G, Zhang Y-P. Genetic relationship of Chinese ethnic populations revealed by mtDNA sequence diversity. *Am. J. Phys. Anthropol.* **118**, 63-76 (2002).
8. Wen B, *et al.* Analyses of genetic structure of Tibeto-Burman populations reveals sex-biased admixture in southern Tibeto-Burmans. *Am. J. Hum. Genet.***74**, 856-865 (2004).
9. Yao Y-G, Zhang Y-P. Phylogeographic analysis of mtDNA variation in four ethnic populations from Yunnan Province: new data and a reappraisal. *J. Hum. Genet.* **47**, 311-318 (2002).
10. Qian YP, *et al.* Mitochondrial DNA polymorphisms in Yunnan nationalities in China. *J. Hum. Genet.* **46**, 211-220 (2001).
11. Yao Y-G, Kong Q-P, Wang C-Y, Zhu C-L, Zhang Y-P. Different matrilineal contributions to genetic structure of ethnic groups in the Silk Road region in China. *Mol. Biol. Evol.* **21**, 2265-2280 (2004).
12. Liu X, Li S. Polymorphism of mitochondrial DNA D-loop region in Chinese Baoan ethnic group. *Journal of the Fourth Military Medical University* **20**, 004 (2003).
13. Liu X, Chen T, Li S. Sequence polymorphism of human mitochondrial DNA control region in Chinese Dongxiang unrelated individuals. *Journal of Medical Colleges of PLA* **19**, (2004).

14. Liu X, Li S. Study on polymorphisms of mitochondrial DNA D-loop region in the Sala population in China. *J Xi'an Jiaotong Univ* **25**, (2004).
15. Wang W, Wise C, Baric T, Black ML, Bittles AH. The origins and genetic structure of three co-resident Chinese Muslim populations: the Salar, Bo'an and Dongxiang. *Hum. Genet.* **113**, 244-252 (2003).
16. Liu X, Li S. Mitochondrial DNA Polymorphism in control region from Chinese Yugu population. *J Xi'an Jiaotong Univ* **16**, (2004).
17. Li C, *et al.* Evidence that a West-East admixed population lived in the Tarim Basin as early as the early Bronze Age. *BMC Biol.* **8**, 15 (2010).
18. Xie C-Z, *et al.* Quantification Polymerase Chain Reaction Designs to Analyze the Ancient Deoxyribonucleic Acid of Xiaohe Cemetery, Xinjiang. *Chinese Journal of Analytical Chemistry* **35**, 5 (2007).
19. Cui Y, Li C, Gao S, Xie C, Zhou H. Early Eurasian migration traces in the Tarim Basin revealed by mtDNA polymorphisms. *Am. J. Phys. Anthropol.* **142**, 558-564 (2010).
20. Cui Y, *et al.* 新疆塔里木盆地早期铁器时代人群的母系遗传结构分析. *Chinese Science Bulletin* **54**, 2912-2919 (2009).
21. He H, *et al.* Study on mtDNA polymorphism of ancient human bone from Hami of Xinjiang, Chian 3200BP. *Acta Anthropologica Sinica* **22**, (2003).
22. Kong Q-P, *et al.* Mitochondrial DNA sequence polymorphisms of five ethnic populations from northern China. *Hum. Genet.* **113**, 391-405 (2003).
23. Zhang YJ, Xu QS, Zheng ZJ, Lin HY, Lee JB. Haplotype diversity in mitochondrial DNA hypervariable region I, II and III in northeast China Han. *Forensic Sci Int* **149**, 267-269 (2005).
24. Wen B, *et al.* Genetic evidence supports demic diffusion of Han culture. *Nature* **431**, 302-305 (2004).
25. Yao Y-G, Kong Q-P, Bandelt H-J, Kivisild T, Zhang Y-P. Phylogeographic differentiation of mitochondrial DNA in Han Chinese. *Am. J. Hum. Genet.***70**, 635-651 (2002).
26. Yao Y-G, Kong Q-P, Man X-Y, Bandelt H-J, Zhang Y-P. Reconstructing the evolutionary history of China: a caveat about inferences drawn from ancient DNA. *Mol. Biol. Evol.* **20**, 214-219 (2003).
27. Tajima A, Sun C-S, Pan I-H, Ishida T, Saitou N, Horai S. Mitochondrial DNA polymorphisms in nine aboriginal groups of Taiwan: implications for the population history of aboriginal Taiwanese. *Hum. Genet.* **113**, 24-33 (2003).
28. Tsai L, Lin C, Lee J, Chang J, Linacre A, Goodwin W. Sequence polymorphism of mitochondrial D-loop DNA in the Taiwanese Han population. *Forensic. Sci. Int.* **119**, 239-247 (2001).
29. Kivisild T, *et al.* The emerging limbs and twigs of the East Asian mtDNA tree. *Mol. Biol. Evol.* **19**, 1737-1751 (2002).
30. Nishimaki Y, Sato K, Fang L, Ma M, Hasekura H, Boettcher B. Sequence polymorphism in the mtDNA HV1 region in Japanese and Chinese. *Leg. Med.* **1**, 238-249 (1999).
31. Oota H, *et al.* Extreme mtDNA homogeneity in continental Asian populations. *Am. J. Phys. Anthropol.* **118**, 146-153 (2002).
32. Wen B, *et al.* Genetic structure of Hmong-Mien speaking populations in East Asia as revealed by mtDNA lineages. *Mol. Biol. Evol.* **22**, 725-734 (2005).
33. Koyama H, *et al.* Mitochondrial sequence haplotype in the Japanese population. *Forensic. Sci. Int.* **125**, 93-96 (2002).
34. Imaizumi K, Parsons TJ, Yoshino M, Holland M. A new database of mitochondrial DNA

hypervariable regions I and II sequences from 162 Japanese individuals. *Int. J. Legal. Med.* **116**, 68-73 (2002).

35. Maruyama S, Minaguchi K, Saitou N. Sequence polymorphisms of the mitochondrial DNA control region and phylogenetic analysis of mtDNA lineages in the Japanese population. *Int. J. Legal. Med.* **117**, 218-225 (2003).
36. Nagai A, Nakamura I, Shiraki F, Bunai Y, Ohya I. Sequence polymorphism of mitochondrial DNA in Japanese individuals from Gifu Prefecture. *Leg. Med.* **5**, S210-S213 (2003).
37. Seo Y, Stradmann-Bellinghausen B, Rittner C, Takahama K, Schneider PM. Sequence polymorphism of mitochondrial DNA control region in Japanese. *Forensic. Sci. Int.* **97**, 155-164 (1998).
38. Mabuchi T, Susukida R, Kido A, Oya M. Typing the 1.1 kb control region of human mitochondrial DNA in Japanese individuals. *J .Forensic. Sci.* **52**, 355-363 (2007).
39. Horai S, *et al.* mtDNA polymorphism in East Asian Populations, with special reference to the peopling of Japan. *Am. J. Hum. Genet.***59**, 579 (1996).
40. Chen F, *et al.* Analysis of mitochondrial DNA polymorphisms in Guangdong Han Chinese. *Forensic. Sci. Int. Genet.* **2**, 150-153 (2008).
41. Cheng B, *et al.* Genetic imprint of the Mongol: signal from phylogeographic analysis of mitochondrial DNA. *J. Hum. Genet.* **53**, 905-913 (2008).
42. Li H, *et al.* Mitochondrial DNA diversity and population differentiation in southern East Asia. *Am. J. Phys. Anthropol.* **134**, 481-488 (2007).
43. Wang WZ, *et al.* Tracing the origins of Hakka and Chaoshanese by mitochondrial DNA analysis. *Am. J. Phys. Anthropol.* **141**, 124-130 (2010).
44. Tajima A, *et al.* Genetic origins of the Ainu inferred from combined DNA analyses of maternal and paternal lineages. *J. hum. genet.* **49**, 187-193 (2004).
45. Gan R-J, *et al.* Pinghua population as an exception of Han Chinese's coherent genetic structure. *J. Hum. Genet.* **53**, 303-313 (2008).
46. Underhill P, Kivisild T. Use of Y-chromosome and mitochondrial DNA population structure in tracing human migrations. *Ann. Rev. Genet.* **41**, 539 - 564 (2007).
47. Tabbada KA, *et al.* Philippine mitochondrial DNA diversity: a populated viaduct between Taiwan and Indonesia? *Mol. Biol. Evol.* **27**, 21-31 (2010).
48. Zhang W, *et al.* A Matrilineal Genetic Legacy from the Last Glacial Maximum Confers Susceptibility to Schizophrenia in Han Chinese. *J. Genet. Genomics.* **41,** 397-407 (2014).
49. Liu J, *et al.* Deciphering the signature of selective constraints on cancerous mitochondrial genome. *Mol. Biol. Evol.* **29**, 1255-1261 (2012).
50. Derenko M, *et al.* Phylogeographic Analysis of Mitochondrial DNA in Northern Asian Populations. *Am. J. Hum. Genet.***81**, 1025-1041 (2007).
51. Ji F, *et al.* Mitochondrial DNA variant associated with Leber hereditary optic neuropathy and high-altitude Tibetans. *Proc. Natl. Acad. Sci.* **109**, 7391-7396 (2012).
52. Gayden T, *et al.* The Himalayas: Barrier and conduit for gene flow. *Am. J. Phys. Anthropol*. **151,** 169-182 (2013).
53. Lee HY, Yoo J-E, Park MJ, Chung U, Kim C-Y, Shin K-J. East Asian mtDNA haplogroup determination in Koreans: Haplogroup-level coding region SNP analysis and subhaplogroup-level control region sequence analysis. *Electrophoresis* **27**, 4408-4418 (2006).
54. Irwin JA, *et al.* Mitochondrial control region sequences from a Vietnamese population sample. *Int*

*J Legal Med* **122**, 257-259 (2008).

55. Summerer M, *et al.* Large-scale mitochondrial DNA analysis in Southeast Asia reveals evolutionary effects of cultural isolation in the multi-ethnic population of Myanmar. *BMC Evol. Biol.* **14**, 17 (2014).
56. Macaulay V, *et al.* Single, Rapid Coastal Settlement of Asia Revealed by Analysis of Complete Mitochondrial Genomes. *Science* **308**, 1034-1036 (2005).
57. Oota H, Settheetham-Ishida W, Tiwawech D, Ishida T, Stoneking M. Human mtDNA and Y-chromosome variation is correlated with matrilocal versus patrilocal residence. *Nat. Genet.* **29**, 20-21 (2001).
58. Peng MS, *et al.* Tracing the Austronesian footprint in Mainland Southeast Asia: a perspective from mitochondrial DNA. *Mol. Biol. Evol.* **27**, 2417-2430 (2010).
59. Zimmermann B, *et al.* Forensic and phylogeographic characterization of mtDNA lineages from northern Thailand (Chiang Mai). *Int J Legal Med* **123**, 495-501 (2009).
60. Black M, Dufall K, Wise C, Sullivan S, Bittles A. Genetic ancestries in northwest Cambodia. *Ann. Hum. Biol.* **33**, 620-627 (2006).
61. Fucharoen G, Fucharoen S, Horai S. Mitochondrial DNA polymorphisms in Thailand. *J. Hum. Genet.* **46**, 115-125 (2001).
62. Lertrit P, *et al.* Genetic history of Southeast Asian populations as revealed by ancient and modern human mitochondrial DNA analysis. *Am. J. Phys. Anthropol.* **137**, 425-440 (2008).
63. Olivieri A, *et al.* The mtDNA legacy of the Levantine early Upper Palaeolithic in Africa. *Science* **314**, 1767-1770 (2006).
64. Maruyama S, Nohira-Koike C, Minaguchi K, Nambiar P. MtDNA control region sequence polymorphisms and phylogenetic analysis of Malay population living in or around Kuala Lumpur in Malaysia. *Int. J. Legal. Med.* **124**, 165-170 (2010).
65. Wong HY, *et al.* Sequence polymorphism of the mitochondrial DNA hypervariable regions I and II in 205 Singapore Malays. *Leg. Med.* **9**, 33-37 (2007).
66. Haslindawaty ARN, Panneerchelvam S, Edinur HA, Norazmi MN, Zafarina Z. Sequence polymorphisms of mtDNA HV1, HV2, and HV3 regions in the Malay population of Peninsular Malaysia. *Int. J. Legal. Med.* **124**, 415-426 (2010).
67. Bodner M, *et al.* Southeast Asian diversity: first insights into the complex mtDNA structure of Laos. *BMC Evol. Biol.* **11**, 49 (2011).
68. Roychoudhury S, *et al.* Genomic structures and population histories of linguistically distinct tribal groups of India. *Hum. Genet.* **109**, 339-350 (2001).
69. Cordaux R, Saha N, Bentley GR, Aunger R, Sirajuddin S, Stoneking M. Mitochondrial DNA analysis reveals diverse histories of tribal populations from India. *Eur J. Hum. Genet.* **11**, 253-264 (2003).
70. Reddy BM, *et al.* Austro-Asiatic tribes of Northeast India provide hitherto missing genetic link between South and Southeast Asia. *PLoS ONE* **2**, e1141 (2007).
71. Quintana-Murci L, *et al.* Where west meets east: the complex mtDNA landscape of the southwest and Central Asian corridor. *Am. J. Hum. Genet.***74**, 827-845 (2004).
72. Metspalu M, *et al.* Most of the extant mtDNA boundaries in south and southwest Asia were likely shaped during the initial settlement of Eurasia by anatomically modern humans. *BMC Genet.* **5**, 26 (2004).
73. Sharma S, Saha A, Rai E, Bhat A, Bamezai R. Human mtDNA hypervariable regions, HVR I and

II, hint at deep common maternal founder and subsequent maternal gene flow in Indian population groups. *J. Hum. Genet.* **50**, 497-506 (2005).

74. Mountain JL, *et al.* Demographic history of India and mtDNA-sequence diversity. *Am. J. Hum. Genet.***56**, 979-992 (1995).
75. Thangaraj K, *et al.* Different population histories of the Mundari-and Mon-Khmer-speaking Austro-Asiatic tribes inferred from the mtDNA 9-bp deletion/insertion polymorphism in Indian populations. *Hum. Genet.* **116**, 507-517 (2005).
76. Barnabas S, Shouche Y, Suresh C. High‐Resolution mtDNA Studies of the Indian Population: Implications for Palaeolithic Settlement of the Indian Subcontinent. *Ann Hum. Genet.* **70**, 42-58 (2006).
77. Rajkumar R, Kashyap V. Haplotype diversity in mitochondrial DNA hypervariable regions I and II in three communities of Southern India. *Forensic. Sci. Int.* **136**, 79-82 (2003).
78. Rajkumar R, Kashyap V. Mitochondrial DNA hypervariable region I and II sequence polymorphism in the Dravidian linguistic group of India. *J. Forensic. Sci.* **48**, 227 (2003).
79. Thangaraj K, Ramana GV, Singh L. Y‐chromosome and mitochondrial DNA polymorphisms in Indian populations. *Electrophoresis* **20**, 1743-1747 (1999).
80. Kivisild T, *et al.* Deep common ancestry of Indian and western-Eurasian mitochondrial DNA lineages. *Curr. Biol.* **9**, 1331-1334 (1999).
81. Quintana-Murci L, Semino O, Bandelt H-J, Passarino G, McElreavey K, Santachiara-Benerecetti AS. Genetic evidence of an early exit of Homo sapiens sapiens from Africa through eastern Africa. *Nat. Genet.* **23**, 437-441 (1999).
82. Thanseem I, *et al.* Genetic affinities among the lower castes and tribal groups of India: inference from Y chromosome and mitochondrial DNA. *BMC Genet.* **7**, 42 (2006).
83. Kumar V, *et al.* Global patterns in human mitochondrial DNA and Y-chromosome variation caused by spatial instability of the local cultural processes. *PLoS Genet.* **2**, e53 (2006).
84. Kivisild T, *et al.* The genetic heritage of the earliest settlers persists both in Indian tribal and caste populations. *Am. J. Hum. Genet.***72**, 313-332 (2003).
85. Watkins W, *et al.* Multiple origins of the mtDNA 9-bp deletion in populations of South India. *Am. J. Phys. Anthropol.* **109**, 147-158 (1999).
86. Mittal B, *et al.* Mitochondrial DNA variation and substructure among the tribal populations of Andhra Pradesh, India. *Am. J. Hum. Biol.* **20**, 683-692 (2008).
87. Gaikwad S, Kashyap V. Molecular insight into the genesis of ranked caste populations of western India based upon polymorphisms across non-recombinant and recombinant regions in genome. *Genome. Biol.* **6**, P10 (2005).
88. Roy S, Thakur C, Majumder PP. Mitochondrial DNA variation in ranked caste groups of Maharashtra (India) and its implication on genetic relationships and origins. *Ann. Hum. Biol.* **30**, 443-454 (2003).
89. Baig M, Khan A, Kulkarni K. Mitochondrial DNA diversity in tribal and caste groups of Maharashtra (India) and its implication on their genetic origins. *Ann. Hum. Genet.* **68**, 453-460 (2004).
90. Kaur I, *et al.* Genomic diversities and affinities among four endogamous groups of Punjab (India) based on autosomal and mitochondrial DNA polymorphisms. *Hum. Biol*. 819-836 (2002).
91. Banerjee J, Trivedi R, Kashyap V. Mitochondrial DNA control region sequence polymorphism in four indigenous tribes of Chotanagpur plateau, India. *Forensic Sci Int* **149**, 271-274 (2005).

92. Sahoo S, Kashyap V. Phylogeography of mitochondrial DNA and Y‐Chromosome haplogroups reveal asymmetric gene flow in populations of Eastern India. *Am. J. Phys. Anthropol.* **131**, 84-97 (2006).
93. Thangaraj K, *et al.* Genetic affinities of the Andaman Islanders, a vanishing human population. *Curr Biol* **13**, 86-93 (2003).
94. Ravi Prasad B, *et al.* Mitochondrial DNA variation in Nicobarese islanders. *Hum Biol* **73**, 715-725 (2001).
95. Wirth T, *et al.* Distinguishing human ethnic groups by means of sequences from Helicobacter pylori: lessons from Ladakh. *Proc. Natl. Acad. Sci.* **101**, 4746-4751 (2004).
96. Eaaswarkhanth M, *et al.* Traces of sub-Saharan and Middle Eastern lineages in Indian Muslim populations. *Eur J. Hum. Genet.* **18**, 354-363 (2009).
97. Fornarino S, *et al.* Mitochondrial and Y-chromosome diversity of the Tharus (Nepal): a reservoir of genetic variation. *BMC Evol Biol* **9**, 154 (2009).
98. Grosheva A, Shneider YV, Morozova IY, Zhukova O, Rychkov SY. Genetic diversity of Besermyans inferred from mitochondrial DNA polymorphism. *Russ J Genet* **49**, 1168-1174 (2013).
99. Malyarchuk B, Derenko M, Denisova G, Kravtsova O. Mitogenomic diversity in Tatars from the Volga-Ural region of Russia. *Mol. Biol. Evol.* **27**, 2220 (2010).
100. Kolman CJ, Sambuughin N, Bermingham E. Mitochondrial DNA analysis of Mongolian populations and implications for the origin of New World founders. *Genetics* **142**, 1321-1334 (1996).
101. Keyser‐Tracqui C, Crubezy E, Pamzsav H, Varga T, Ludes B. Population origins in Mongolia: genetic structure analysis of ancient and modern DNA. *Am. J. Phys. Anthropol.* **131**, 272-281 (2006).
102. Derenko MV, *et al.* Mitochondrial DNA variation in two South Siberian Aboriginal populations: implications for the genetic history of North Asia. *Hum Biol*, 945-973 (2000).
103. Malyarchuk B, Derenko M. Mitochondrial DNA variability in Russians and Ukrainians: Implication to the origin of the Eastern Slavs. *Ann. Hum. Genet.* **65**, 63-78 (2001).
104. Derenko M, *et al.* Diversity of mitochondrial DNA lineages in South Siberia. *Ann Hum. Genet.* **67**, 391-411 (2003).
105. Pakendorf B, *et al.* Mitochondrial DNA evidence for admixed origins of central Siberian populations. *Am. J. Phys. Anthropol.* **120**, 211-224 (2003).
106. Fedorova S, Bermisheva M, Villems R, Maksimova N, Khusnutdinova E. Analysis of mitochondrial DNA haplotypes in yakut population]. *Mol Biol (Mosk)* **37**, 643 (2003).
107. Derenko M, Shields G. Diversity of mitochondrial DNA nucleotide sequences in three groups of aboriginal inhabitants of Northern Asia]. *Mol Biol (Mosk)* **31**, 784 (1997).
108. Derbeneva O, Starikovskaia E, Volod'ko N, Wallace D, Sukernik R. Mitochondrial DNA variation in Kets and Nganasans and the early peoples of Northern Eurasia. *Genetika* **38**, 1554-1560 (2002).
109. Bermisheva M, Tambets K, Villems R, Khusnutdinova E. Diversity of mitochondrial DNA haplotypes in ethnic populations of the Volga-Ural region of Russia. *Mol Biol (Mosk)* **36**, 990-1001 (2001).
110. Starikovskaya EB, *et al.* Mitochondrial DNA diversity in indigenous populations of the southern extent of Siberia, and the origins of Native American haplogroups. *Ann Hum. Genet.* **69**, 67-89 (2005).
111. Volodko NV, *et al.* Mitochondrial genome diversity in arctic Siberians, with particular reference to

the evolutionary history of Beringia and Pleistocenic peopling of the Americas. *Am. J. Hum. Genet.***82**, 1084-1100 (2008).

112. Dulik Matthew C, *et al.* Mitochondrial DNA and Y Chromosome Variation Provides Evidence for a Recent Common Ancestry between Native Americans and Indigenous Altaians. *Am. J. Hum. Genet.***90**, 229-246 (2012).
113. Derenko M, *et al.* Complete Mitochondrial DNA Analysis of Eastern Eurasian Haplogroups Rarely Found in Populations of Northern Asia and Eastern Europe. *PLoS ONE* **7**, e32179 (2012).
114. Pimenoff VN, Comas D, Palo JU, Vershubsky G, Kozlov A, Sajantila A. Northwest Siberian Khanty and Mansi in the junction of West and East Eurasian gene pools as revealed by uniparental markers. *Eur J. Hum. Genet.* **16**, 1254-1264 (2008).
115. Derenko M, *et al.* Origin and Post-Glacial Dispersal of Mitochondrial DNA Haplogroups C and D in Northern Asia. *PLoS ONE* **5**, e15214 (2010).
116. Sukernik RI, Volodko NV, Mazunin IO, Eltsov NP, Dryomov SV, Starikovskaya EB. Mitochondrial genome diversity in the tubalar, even, and ulchi: Contribution to prehistory of native siberians and their affinities to native americans. *Am. J. Phys. Anthropol.* **148**, 123-138 (2012).
117. Comas D, *et al.* Admixture, migrations, and dispersals in Central Asia: evidence from maternal DNA lineages. *Eur J. Hum. Genet.* **12**, 495-504 (2004).
118. Irwin JA, *et al.* The mtDNA composition of Uzbekistan: a microcosm of Central Asian patterns. *Int. J. Legal. Med.* **124**, 195-204 (2010).
119. Chaix R, *et al.* From social to genetic structures in central Asia. *Curr Biol* **17**, 43-48 (2007).
120. Lalueza-Fox C, *et al.* Unravelling migrations in the steppe: mitochondrial DNA sequences from ancient Central Asians. *Proc R Soc Lond B Biol Sci* **271**, 941-948 (2004).
121. Al-Zahery N, Saunier J, Ellingson K, Parson W, Parsons TJ, Irwin JA. Characterization of mitochondrial DNA control region lineages in Iraq. *Int. J. Legal. Med.* **127**, 373-375 (2013).
122. Scheible M, Alenizi M, Sturk-Andreaggi K, Coble MD, Ismael S, Irwin JA. Mitochondrial DNA control region variation in a Kuwaiti population sample. *Forensic Sci Int Genet* **5**, e112-e113 (2011).
123. Non AL, Al‐Meeri A, Raaum RL, Sanchez LF, Mulligan CJ. Mitochondrial DNA reveals distinct evolutionary histories for Jewish populations in Yemen and Ethiopia. *Am. J. Phys. Anthropol.* **144**, 1-10 (2011).
124. Behar DM, *et al.* Counting the founders: the matrilineal genetic ancestry of the Jewish Diaspora. *PLoS ONE* **3**, e2062 (2008).

**Supplementary Table S8. HVS data for networks.**

| Sample name | HG | HVS-I (16000+) | HVS II | Some Coding-Region Polymorphisms | Location | Nation | Reference |
|---|---|---|---|---|---|---|---|
| Jarai08 | M24 | 223 | 73 146 195 263 310+C | G10398A C10400T T15601C | Cambodia | Jarai | 1 |
| Jarai20 | M24 | 223 | 73 146 195 263 310+C | G10398A C10400T T15601C | Cambodia | Jarai | 1 |
| Lao74 | M24 | 223 | 73 146 195 263 310+C | G10398A C10400T T15601C | Cambodia | Lao | 1 |
| Burman416* | M24 | 223 311 519 | 73 146 152 195 204 263 315+C 489 | 15012-15749=15043 15301 15326 15601 | Sagaing, Myanmar | Naga | This study |
| Burman420 | M24 | 223 311 519 | 73 146 152 195 204 263 315+C 489 | 15013-15800=15043 15301 15326 15601 | Sagaing, Myanmar | Naga | This study |
| Burman421 | M24 | 223 311 519 | 73 146 152 195 204 263 6315+C 489 | 15011-15890=15043 15301 15326 15601 | Sagaing, Myanmar | Naga | This study |
| Burman428 | M24 | 223 311 519 | 73 146 152 195 204 263 315+C 489 | 15010-15780=15043 15301 15326 15601 | Sagaing, Myanmar | Naga | This study |
| Burman429 | M24 | 223 311 519 | 73 146 152 195 204 263 315+C 489 | 15012-15999=15043 15301 15326 15601 | Sagaing, Myanmar | Naga | This study |
| Burman433 | M24 | 223 311 519 | 73 146 152 195 204 263 315+C 489 | 15011-15800=15043 15301 15326 15601 | Sagaing, Myanmar | Naga | This study |
| Burman436 | M24 | 223 311 519 | 73 146 152 195 204 263 315+C 489 | 15412-15965=15601 | Sagaing, Myanmar | Naga | This study |
| Burman441 | M24 | 223 311 519 | 73 146 152 195 204 263 315+C 489 | 15011-15928=15043 15301 15326 15601 | Sagaing, Myanmar | Naga | This study |
| Burman445 | M24 | 223 311 519 | 73 146 152 195 204 263 315+C 489 | 15011-15725=15043 15301 15326 15601 | Sagaing, Myanmar | Naga | This study |
| Burman471 | M24 | 223 311 519 | 73 146 152 195 204 263 315+C 489 | 15012-15900=15043 15301 15326 15601 | Sagaing , Myanmar | Naga | This study |
| Burman079 | M24 | 075 223 311 519 | 73 146 152 195 263 309+C 489 | 15012-15800=15043 15301 15326 15601 15607 | Sagaing , Myanmar | Burmans | This study |
| Burman702* | M24 | 075 223 311 327 519 | 73 146 152 195 263 315+C | | Bago, Myanmar | Burmans | This study |
| Khmer02* | M24 | 223 311 | 73 146 152 195 263 309+C 310 | G10398A C10400T G15043A | Cambodia | Khmer | 1 |
| Khmer44 | M24 | 223 311 | 73 146 152 195 263 309+C | A6581G G10398A C10400T G13359A G15043A G15314A | Cambodia | Khmer | 1 |
| Phnong33 | M24 | 223 311 | 73 146 152 195 263 309+C | A6581G G10398A C10400T G13359A G15043A G15314A | Cambodia | Phnong | 1 |
| Stieng26 | M24 | 223 311 | 73 146 152 195 263 | G10398A C10400T G15043A G15314A | Cambodia | Stieng | 1 |
| K23720 | M24 | 086 223 519 | 73 146 195 263 315+C 489 522-523d 750 | 4524-5105=4769 5176AluI+ | Naxay thong,Elay, Laos | lao loum | Our unpublished data |
| K24460 | M24 | 086 223 311 519 | 73 146 152 195 263 315+C 489 499 522-523d | 4918-5449=rCRS; 5774-6341=rCRS | Hadxay fong xiengkhuan, Laos | lao loum | Our unpublished data |
| K24485 | M24 | 086 223 519 | 73 146 195 263 315+C 489 522-523d 750 | 5774-6342=rCRS | Xaysed tha Thadluangonua, Laos | lao loum | Our unpublished data |
| K25049 | M24 | 223 311 519 | 73 146 152 195 263 309+2C 315+C 489 | | chanpasak, Laos | lao loum | Our unpublished data |
| K25287 | M24 | 223 311 519 | 73 143 146 152 195 263 309+C 315+C 414 445 489 | | Bokea, Laos | lao sung | Our unpublished data |
| DaiDP3* | M24 | 086 223 278 519 | 73 146 263 315+C (489 523 524d) | +5176AluI-4831HhaI | Lancang , China | Dai | Our unpublished data |
| Jarai06* | M24 | 086 223 519 | 73 146 195 263 310+C | G10398A C10400T T15601C | Cambodia | Jarai | 1 |
| Burman755* | M24 | 086 223 278 519 | 73 146 195 263 315+C | | Ayeyarwady, Myanmar | Burmans | Our unpublished data |
| TB 6* | M24 | 223 293C 311 | | | Palawan | Palawan Non-Negrito | 2 |
| TB 10 | M24 | 223 293C 311 | | | Palawan | Palawan Non-Negrito | 2 |
| TB 12 | M24 | 223 293C 311 | | | Palawan | Palawan Non-Negrito | 2 |
| TB 13 | M24 | 223 293C 311 | | | Palawan | Palawan Non-Negrito | 2 |
| TB 14 | M24 | 223 293C 311 | | | Palawan | Palawan Non-Negrito | 2 |
| DJY759 | M24 | 223 311 519 | 73 146 152 195 263 309+CC 315+C | | Dujiangyan,Sichuan, China | Han | Our unpublished data |
| 2390 | M24 | 223 311 519 | 73 146 152 195 263 309+C 315+C | | Dujiangyan,Sichuan, China | Han | Our unpublished data |
| Burman732* | M24 | 086 223 278 519 | 73 146 195 263 309+C 315+C | 5831-6103=rCRS | Ayeyarwady, Myanmar | Burmans | This study |
| Burman825 | M24 | 086 223 278 519 | 73 146 195 263 309+C 315+C 489 522-523d | 5811-6480=rCRS | Rakhine, Myanmar | Rakhine | This study |
| AS01* | M24 | 223 311 519 | | | Cambodian | | 3 |
| Burman543 | M91 | 129 223 287 311 327A | 64 73 93 146 189 200 263 309+C 315+C 485 489 | 9218-9700=9509 9540 9554 | Magway, Myanmar | Burmans | Our unpublished data |
| Burman575* | M91 | 129 223 287 311 327A | 64 73 93 146 189 200 263 309+CC 315+C 485 489 | | Magway, Myanmar | Burmans | Our unpublished data |
| Burman593 | M91 | 129 223 287 311 327A | 64 73 93 146 189 200 263 309+C 315+C 485 489 | 9256-9650=9509 9540 9554 | Magway, Myanmar | Burmans | Our unpublished data |
| Burman521 | M91 | 129 223 311 327A | 64 73 93 200 263 309+C 315+C 485 489 | 9278-9550=9509 9540 | Magway, Myanmar | Burmans | Our unpublished data |
| Burman528 | M91 | 129 223 311 327A | 64 73 93 200 263 309+C 315+C 485 489 | 9224-9700=9509 9540 9554 | Magway, Myanmar | Burmans | Our unpublished data |
| Burman531* | M91 | 129 223 311 327A | 64 73 93 200 263 309+C 315+C 485 489 | | Magway, Myanmar | Burmans | Our unpublished data |
| Burman594 | M91 | 129 223 311 327A | 64 73 93 200 263 309+C 315+C 485 489 | 9222-936=9509 9540 9554 | Magway, Myanmar | Burmans | Our unpublished data |

| Sample name | HG | HVS-I (16000+) | HVS II | Some Coding-Region Polymorphisms | Location | Nation | Reference |
|---|---|---|---|---|---|---|---|
| NQ-T2819 | M91 | 154 223 287 327A 355 | 73 93 146 152 200 234 249d 263 282 309+C 315+C 489 | 3391HaeIII- | Nagqu, Tibet, China | Tibetan | 4 |
| NuCW2 | M91 | 129 223 287 311 327A | 64 73 93 189 200 263 315+C | +10397AluI-10644RsaI | Nujiang , China | Nu | Our unpublished data |
| LP262 | M91 | 154 223 287 327A 355 | 73 93 152 200 234 249d 263 282 315+C 489 750 | | Yuxi, Yunnan, China | Patient | 5 |
| 12C* | M91 | 129 223 287 311 327A | 64 73 93 189 200 263 309+CC 315+C 485 489 | | Yunnan, China | Han | 6 |
| MMR026* | M91 | 223 287 327 | 73 93 95 200 263 315+C 489 | | Yangon Division, Myanmar | Bamar | 7 |
| MMR302* | M91 | 129 223 287 311 327A | 61 62 65+T 73 93 189 200 227 263 309+C 315+C 485 489 | | Tanintharyi Division, Myanmar | Da Wai | 7 |
| Stieng29* | M91 | 069 223 287 299 327A | 73 93 263 309+C 310+C | G10398A C10400T | Cambodian | Stieng | 1 |
| Stieng37* | M91 | 069 223 287 299 327A | 73 93 263 309+C 310+C | G10398A C10400T | Cambodian | Stieng | 1 |
| Stieng38* | M91 | 069 223 287 299 327A | 73 93 263 309+C 310+C | G10398A C10400T | Cambodian | Stieng | 1 |
| Stieng46* | M91 | 069 223 287 299 327A | 73 93 263 309+C 310+C | G10398A C10400T | Cambodian | Stieng | 1 |
| Burman606* | M90 | 086 223 381 390 | 73 150 227 263 309+C 315+C 489 | | Magway, Myanmar | Burmans | This study |
| Burman634 | M90 | 086 223 381 390 | 73 150 227 263 309+C 315+C 489 | 5831-6278=5910 6023 6253 | Magway, Myanmar | Burmans | This study |
| Burman636 | M90 | 086 223 381 390 | 73 150 227 263 309+C 315+C 489 | 5821-6204=5910 6023 | Magway, Myanmar | Burmans | This study |
| MMR206* | M90 | 086 111 223 381 390 | 73 150 263 309+C 315+C 489 | | Kayin State, Myanmar | Karen | 7 |
| Deang ND3 | M90 | 223 381 390 | 73 150 263 309+C 315+C 489 | +12406HpaI, -4831HhaI, -663HaeIII, -3391HaeIII, +10397AluI, 5851-6468=5910 6023 6253 | Yunnan, China | Deang | Our unpublished data |
| Burman209 | M90 | 125 223 381 390 | 61A 62 73 146 152 263 309+C 315+C 489 | 11033-11798=11440 11719; 14071-14697=rCRS | Sagaing, Myanmar | Burmans | This study |
| Thai37* | M90 | 223 381 390 | 61A 62 73 146 152 263 309+CC 315+C 489 | | Thailand | | 8 |
| thai | M90 | 223 381 390 | | | Northern Thailand, Thailand | | 9 |
| Burman085 | M90 | 223 381 390 | 61A 62 73 146 152 263 309+C 315+C 489 | 5813-6362=5910 6023 6253 | Sagaing , Myanmar | Burmans | This study |
| Burman086 | M90 | 223 381 390 | 61A 62 73 146 152 263 309+C 315+C 489 | 5811-6363=5910 6023 6253 | Sagaing , Myanmar | Burmans | This study |
| Burman088* | M90 | 223 381 390 | 61A 62 73 146 152 263 309+C 315+C 489 | | Sagaing , Myanmar | Burmans | This study |
| Burman176 | M90 | 223 381 390 | 61A 62 73 146 152 | | Sagaing, Myanmar | Burmans | This study |
| Burman189 | M90 | 223 381 390 | 61A 62 73 146 152 263 309+C 315+C 489 | 5843-6290=5910 6023 6253 | Sagaing, Myanmar | Burmans | This study |
| Burman190 | M90 | 223 381 390 | 61A 62 73 146 152 263 309+C 315+C 489 | 11023-11821=11719; 14077-14694 =rCRS | Sagaing, Myanmar | Burmans | This study |
| Burman194 | M90 | 223 381 390 | 61A 62 73 146 152 263 309+C 315+C 489 | 11025-11779=11719; 14072-14338 =rCRS | Sagaing, Myanmar | Burmans | This study |
| Burman195 | M90 | 223 381 390 | 61A 62 73 146 152 263 309+C 315+C 489 | | Sagaing, Myanmar | Burmans | This study |
| Burman205 | M90 | 223 381 390 | 61A 62 73 146 152 263 309+C 315+C 489 | 5831-6270=5910 6023 6253 | Sagaing, Myanmar | Burmans | This study |
| Burman211 | M90 | 223 381 390 | 61A 62 73 146 152 263 309+C 315+C 489 | 5831-6362=5910 6023 6253 | Sagaing, Myanmar | Burmans | This study |
| Burman220 | M90 | 223 381 390 | 61A 62 73 146 152 263 309+C 315+C 489 522- | 5814-6280=5910 6023 6253 | Sagaing, Myanmar | Burmans | This study |
| Burman221 | M90 | 223 381 390 | 61A 62 73 146 152 263 309+C 315+C 489 | 11033-11864=11719; 14073-14694=rCRS | Sagaing, Myanmar | Burmans | This study |
| Burman222 | M90 | 223 381 390 | 61A 62 73 146 152 263 309+C 315+C 489 | 5813-6059=5910 6023 | Sagaing, Myanmar | Burmans | This study |
| Burman623 | M90 | 223 381 390 | 61A 62 73 146 152 263 309+C 315+C 489 | 5833-6365=5910 6023 6253 | Magway, Myanmar | Burmans | This study |
| Burman625 | M90 | 223 381 390 | 61A 62 73 146 152 263 309+C 315+C 489 | 5811-6270=5910 6023 6253 | Magway, Myanmar | Burmans | This study |
| MMR225* | M90 | 223 311 381 390 | 61A 62 73 146 152 263 309+C 315+C 489 | | Myanmar | Bamar | 7 |
| MMR007* | M90 | 223 381 390 | 61A 62 73 146 152 263 309+C 315+C 489 | | Kachin State, Myanmar | Bamar | 7 |
| MMR187* | M90 | 174 223 274 320 362 381 390 | 73 263 309+C 315+C 489 | | Bago Division, Myanmar | Karen | 7 |
| Burman243 | M72 | 129 166d 213 214 223 526 | 73 263 315+C 489 | 15422-16035=15497 15644 15820 | Chin, Myanmar | Chin | This study |
| Burman350 | M72 | 129 166d 214 223 526 | 63 73 263 315+C 489 | 15416-16024=15497 15644 15820 | Chin, Myanmar | Chin | This study |
| Burman376* | M72 | 129 166d 214 223 526 | 63 73 263 315+C 489 | | Chin, Myanmar | Chin | This study |
| Burman377 | M72 | 129 166d 214 223 526 | 63 64 73 263 315+C 489 | 15409-16024=15497 15644 15820 | Chin, Myanmar | Chin | This study |
| NE62-2 | M72 | 129 166d 213 214 223 342 526 | 73 263 315+C 489 | | Mizoram, India | Kuki tribe | Our unpublished data |
| NE64-1 | M72 | 129 166d 213 214 223 342 526 | 73 263 315+C 489 | 15412-16055=15497 15644 15820 | Mizoram, India | Kuki tribe | Our unpublished data |
| Burman253* | M72 | 129 166d 213 214 223 342 526 | 73 263 315+C 489 | | Chin, Myanmar | Chin | This study |
| Burman329 | M72 | 129 166d 213 214 223 342 526 | 73 263 315+C 489 | | Chin, Myanmar | Chin | This study |

| Sample name | HG | HVS-I (16000+) | HVS II | Some Coding-Region Polymorphisms | Location | Nation | Reference |
|---|---|---|---|---|---|---|---|
| Kinh101* | M72 | 166d 209 214 223 260 311 | | 9620-10730=10398 10400 | Hanoi, Vietnam | | 10 |
| (03B)0231* | M72 | 093 166d 214 223 249 278 | 73 263 315+C | 10171-10659=10398 10400 | Guangdong, China | Han | 11 |
| Luzon | M72 | 166d 172 214 223 | | | Philippine | Luzon | 12 |
| T-16 | M72 | 214 223 344 | | 10398 10400 15644 15820 | Chittagong hill tract, Bangladesh | Tripura | 13 |
| T-17 | M72 | 214 223 344 | | 10398 10400 15644 15820 | Chittagong hill tract, Bangladesh | Tripura | 13 |
| Mataram | M72 | 124 166d 214 223 | | | Mataram, Indonesian | Mataram | 14 |
| Khmer83 | M72 | 124 166d 175 214 223 519 | 73 263 309+C | G10398A C10400T A15644G C15820T | Cambodia | Khmer | 1 |
| Phnong32 | M72 | 124 166d 175 214 223 519 | 73 263 309+C | G10398A C10400T A15644G C15820T | Cambodia | Phnong | 1 |
| Phnong47 | M72 | 124 166d 175 214 223 519 | 73 263 309+C | G10398A C10400T A15644G C15820T | Cambodia | Phnong | 1 |
| Phnong54 | M72 | 124 166d 175 214 223 519 | 73 263 309+C 310+C | G10398A C10400T A15644G C15820T | Cambodia | Phnong | 1 |
| K24353 | M72 | 166d 214 223 | 73 263 309+C 315+C 489 | 15427-15819=15644 15811 | Xaysed tha Hongsuphab, Laos | lao loum | Our unpublished data |
| K25305 | M72 | 166d 214 223 | 73 263 309+C 315+C 489 750 | | Phongsali, Laos | lao sung | Our unpublished data |
| PH368* | M72 | 166d 172 214 223 | 73 150 263 309+C 315+C 489 | | Philippines | | 12 |
| MMR261* | M72 | 166d 214 223 311 390R 519 | 73 263 309+C 315+C 489 | | Bago Division, Myanmar | Bamar | 7 |
| MMR289 | M72 | 166d 214 223 | 73 263 309+C 315+C 489 | | Bago Division, Myanmar | Bamar | 7 |
| Burman582* | M58 | 183 189 223 266 295 519 | 73 143 153 263 309+C 315+C 489 | | Magway, Myanmar | Burmans | This study |
| Burman720 | M58 | 183 189 193d 223 266 295 519 | 73 143 153 263 309+C 315+C 489 | 15617-16192=15924 16183 16189 | Ayeyarwady, Myanmar | Burmans | This study |
| Burman745 | M58 | 183 189 223 266 295 519 | 73 143 153 263 309+C 315+C 489 | 15625-16200=15924 16183 16189 | Ayeyarwady, Myanmar | Burmans | This study |
| Burman763 | M58 | 183 189 223 266 295 519 | 73 143 153 263 309+C 3159+C 489 | 15616-16192=15924 16183 16189 | Ayeyarwady, Myanmar | Burmans | This study |
| Burman052* | M58 | 129 183 218 223 293C 311 519 | 73 146 263 279 309+C 315+C 489 | 4520-5220=4769; 15016-15472=15043 15301 15326 | Sagaing, Myanmar | Burmans | This study |
| NE09-3 | M58 | 129 183 218 223 293C 311 519 | 73 146 263 279 315+C | 4519-4970/16628-16000=4769 15901 | Mizoram, India | Chakma tribe | Our unpublished data |
| NE20-2 | M58 | 129 183 218 223 293C 311 519 | 73 146 263 279 315+C 489 | 4529-5210=4769 5131+T | Mizoram, India | Chakma tribe | Our unpublished data |
| D147 | M58 | 129 183 218 223 293C 311 519 | 73 146 263 279 315+C 489 | 5440-5793=5460 5662 | Dinajpur, Bangladesh | Bengali_D | Our unpublished data |
| Thoti112 | M58 | 093 223 230 243 270 319 352 | | | Andhra Pradesh, India | Thoti | 15 |
| WA103* | M58 | 223 230 243 270 319 352 519 | 73 152 263 309+C 315+C 331 489 | | Northeast India, India | Wanchoo | 16 |
| SA220 | M58 | 086 223 230 243 270 319 352 | 73 263 151 195 199 204 | | India | Santhal | Our unpublished data |
| MMR127* | M58 | 183 189 193+2C 223 266 295 519 | 73 146 153 263 309+C 315+C 489 | | Kayin State, Myanmar | Mon | 7 |
| TH2E8 | M83 | 129 147 203 223 319 519 527 | 73 146 152 182 263 315+C 356+C 489 | 4769 8701 8860 9540 10400 11719 12426 12705 | Chiang Mai, Thailand | | 17 |
| TH1D4 | M83 | 129 223 | 73 146 263 315+C 356+C 489 | 4769 8701 8860 8997 9540 9797 10400 11719 12426 12705 | Chiang Mai, Thailand | | 17 |
| Ra43 | M83 | 129 223 320 | 73 146 207 263 356+C | | West Bengal, India | Rajbhansi | Our unpublished data |
| Burman484 * | M83 | 129 223 320 | 73 146 207 263 309+C 315+C 356+C 489 | | Sagaing, Myanmar | Naga | This study |
| Burman512 | M83 | 129 223 320 | 73 146 263 309+CC 315+C 489 | 7904-8479=8143 8271 8307 | Sagaing, Myanmar | Naga | This study |
| Burman615 | M83 | 129 223 519 527 | 73 263 309+C 315+C 356+C 489 | 7903-8488=8059 8143 8307 | Magway, Myanmar | Burmans | This study |
| Burman622* | M83 | 129 223 519 527 | 73 263 309+C 315+C | | Magway, Myanmar | Burmans | This study |
| RJ230 | M83 | 209 223 320 | 73 146 207 263 | | West Bengal, India | Rajbhansi | Our unpublished data |
| Ra90 | M83 | 223 320 | 73 146 207 263 356+C | | West Bengal, India | Rajbhansi | Our unpublished data |
| SW16 | M83 | 223 320 | 73 146 207 263 356+C | | West Bengal, India | Rajbhansi | Our unpublished data |
| RV34 | M83 | 223 320 | 73 146 207 263 356+C | | India | Rabha | Our unpublished data |
| RV36 | M83 | 223 320 | 73 146 207 263 356+C | | India | Rabha | Our unpublished data |
| RV51 | M83 | 223 320 | 73 146 207 263 356+C | | India | Rabha | Our unpublished data |
| Sc52 | M83 | 223 320 | 73 146 207 263 356+C | | India | | Our unpublished data |
| BN56/3 | M83 | 223 320 | 73 146 207 263 356+C | | Bangladesh | | Our unpublished data |
| RJ37 | M83 | 223 320 | 73 152 207 263 356+C | | West Bengal, India | Rajbhansi | Our unpublished data |
| RJ131 | M83 | 223 320 | 73 146 207 263 | | West Bengal, India | Rajbhansi | Our unpublished data |
| RV16 | M83 | 223 320 | | | India | Rabha | Our unpublished data |
| Burman501 * | M83 | 223 320 | 73 146 207 263 315+C 356+C 489 | | Sagaing, Myanmar | Naga | This study |

| Sample name | HG | HVS-I (16000+) | HVS II | Some Coding-Region Polymorphisms | Location | Nation | Reference |
|---|---|---|---|---|---|---|---|
| Burman482 | M83 | 223 320 | 73 146 207 263 315+C 356+C 489 | 7908-8442=8143 8271 8307 | Sagaing, Myanmar | Naga | This study |
| Burman513 | M83 | 223 320 | 73 146 207 263 315+C 356+C 489 | 7909-8607=8143 8271 8307 | Sagaing, Myanmar | Naga | This study |
| Tripura20 | M83 | 039T 223 309 320 | | | Tipperah India, India | Tipperah | 15 |
| Tripura8 | M83 | 064A 223 320 | | | Tipperah India, India | Tipperah | 15 |
| Burman767 | M83 | 311 319 357 | 73 152 263 315+C 356+C | 15412-15998=15670 15941 | Ayeyarwady, Myanmar | Burmans | This study |
| Burman775 | M83 | 311 319 357 | 73 152 263 315+C 356+C 489 | 15426-16056=15670 15941 | Ayeyarwady, Myanmar | Burmans | This study |
| Burman801* | M83 | 311 319 357 | 73 152 263 315+C 356+C 489 | | Rakhine, Myanmar | Rakhine | This study |
| Burman811 | M83 | 311 319 357 | 73 152 263 315+C 356+C 489 | 15407-16052=15670 15941 | Rakhine, Myanmar | Rakhine | This study |
| Burman818 | M83 | 311 319 357 | 73 152 263 315+C 356+C 489 | 15426-15710=15670 | Rakhine, Myanmar | Rakhine | This study |
| Tripura6 | M83 | 223 298 311 319 357 | | | Tipperah India, India | Tipperah | 15 |
| ME13 | M83 | 086 129 223 311 319 | 73 263 356+C | | India | Mech | Our unpublished data |
| ME10 | M83 | 086 129 223 311 319 | 73 263 356+C | | India | Mech | Our unpublished data |
| ME16 | M83 | 086 129 223 311 319 | 73 263 356+C | | India | Mech | Our unpublished data |
| ME7 | M83 | 086 129 223 311 319 | 73 263 356+C | | India | Mech | Our unpublished data |
| BO11/2 | M83 | 086 129 223 311 319 | 73 263 356+C | | Assam northeast India, India | Bodo | Our unpublished data |
| BO13/1 | M83 | 086 129 223 311 319 | 73 263 356+C | | Assam northeast India, India | Bodo | Our unpublished data |
| BO15/1 | M83 | 086 129 223 311 319 | 73 263 356+C | | Assam northeast India, India | Bodo | Our unpublished data |
| BO19/2 | M83 | 086 129 223 311 319 | 73 263 356+C | | Assam northeast India, India | Bodo | Our unpublished data |
| BO2/2 | M83 | 086 129 223 311 319 | 73 263 356+C | | Assam northeast India, India | Bodo | Our unpublished data |
| BO33/2 | M83 | 086 129 223 311 319 | 73 263 356+C | | Assam northeast India, India | Bodo | Our unpublished data |
| MMR211* | M83 | 223 163 362 | | | Kayin State, Myanmar | Bamar | 7 |
| Burman681* | M45 | 223 519 | 73 146 152 263 309+C 315+C 489 | | Bago , Myanmar | Burmans | This study |
| AchangKU4* | M45 | 093 193 223 519 | 73 146 152 263 309+C 315+C 489 | +12406HpaI-4831HhaI -3391HaeIII+10397AluI | Yunnan, China | Achang | Our unpublished data |
| JingpoAE1 | M45 | 193 223 519 | 73 146 152 263 309+C 315+C | +12406HpaI-663HaeIII+10397AluI | Yunnan, China | Jingpo | Our unpublished data |
| JingpoAN2* | M45 | 193 223 519 | 73 146 152 263 309+C 315+C | -10397AluI | Yunnan, China | Jingpo | Our unpublished data |
| JingpoCN1* | M45 | 093 193 223 519 | 73 146 152 263 309+CC 315+C | +12406HpaI-3391HaeIII+10397AluI | Yunnan, China | Jingpo | Our unpublished data |
| Java | M45 | 086 129 209 223 237 | | | Java | Javanese | 14 |
| SU59 | M45 | 145 192 223 300 316 | 73 146 263 489 | 8701-9180-10398-10400-12705-15043-15301-15326 | North India | Sunni Muslim | 18 |
| Nepalese075 | M45 | 223 266 357 | 73 189 195 263 309+C 315+C | | Kathmandu, Nepal | | Our unpublished data |
| LP268 | M45 | 183C 189 193+C 223 300 519 | 73 143 146 152 263 315+C 489 750 | | Yuxi, Yunnan, China | Patient | 5 |
| Burman198 | M45 | 223 519 | 73 146 152 153 234 263 309+C 315+C 489 | 1803-2369=rCRS; 2432-4962=2706 3504 3669 3808 4734 4769; 5309-5928=rCRS; 11023-11797=11719; 14075-14701=rCRS | Sagaing, Myanmar | Burmans | Our unpublished data |
| SC41* | M45 | 145 150 182C 183C 189 223 519 | | | China | | 19 |
| HK83* | M45 | 183C 189 193+C 223 300 519 | | | India | | 16 |
| HK93* | M45 | 154 183C 189 223 300 519 | | | India | | 16 |
| KK35* | M45 | 178A 183C 189 223 300 519 | | | India | | 16 |
| KK42* | M45 | 178A 182C 183C 189 223 300 519 | | | India | | 16 |
| MN6* | M45 | 179 183C 189 223 300 519 | | | India | | 16 |
| MN7* | M45 | 179 183C 189 223 266 300 519 | | | India | | 16 |
| MN101* | M45 | 183C 189 223 300 519 | | | India | | 16 |
| MN72* | M45 | 182C 183C 189 223 300 519 | | | India | | 16 |
| HND8913(Tor463 | M45 | 129 189 192 223 300 362 519 | | | India | | 20 |
| Tu65* | M45 | 179 183C 189 223 300 519 | | | Qinghai, China | Tu | 21 |
| TH2F6 | M55 | 111 223 243 274 311 362 381C | 63 64 66 73 263 309+2C 315+C 489 | 4491 10400 | Chiang Mai, Thailand | | 17 |
| Burman386* | M55 | 136 189 217 223 319 381 | 73 94 173 204 263 315+C 482 489 522-523d | 9230-9728=9477 9540 | Chin, Myanmar | Chin | Our unpublished data |
| Naga29 | M55 | 136 217 223 319 381 | | | Nagaland India, India | Naga | 15 |

| Sample name | HG | HVS-I (16000+) | HVS II | Some Coding-Region Polymorphisms | Location | Nation | Reference |
|---|---|---|---|---|---|---|---|
| Mal-113 | M55 | 136 217 223 319 381 | 73 94 173 204 263 315+C 482 489 | | Kuala Lumpur Malaysia, Malaysia | Mal | 22 |
| Burman146 | M55 | 136 217 223 319 381 | 73 94 173 204 263 315+C 469+A 482 489 | 9243-9539=9447 | Chin, Myanmar | Chin | This study |
| Burman164 | M55 | 136 217 223 319 381 | 73 94 173 204 263 315+C 482 489 | 9250-9551=9447 9540 | Chin, Myanmar | Chin | This study |
| Burman172 | M55 | 136 217 223 319 381 | 73 94 173 204 263 315+C 482 489 | 9217-9660=9447 9540 | Chin, Myanmar | Chin | This study |
| Burman296 | M55 | 136 217 223 319 381 | 73 94 173 204 263 315+C 482 489 | 9240-9731=9447 9540 | Chin, Myanmar | Chin | This study |
| Burman346* | M55 | 136 217 223 319 381 | 73 94 173 204 263 315+C 482 489 522-523d | | Chin, Myanmar | Chin | This study |
| Burman393 | M55 | 136 217 223 319 381 | 73 94 173 204 263 315+C 482 489 522-523d | 9228-9592=9447 9540 | Chin, Myanmar | Chin | This study |
| Burman398 | M55 | 136 217 223 319 381 | 73 94 173 204 263 315+C 482 489 522-523d | 9241-9765=9447 9540 | Chin, Myanmar | Chin | This study |
| Burman644 | M55 | 136 217 223 319 381 | 73 94 173 204 263 315+C 482 489 | 9228-9673=9447 9540 | Magway, Myanmar | Chin | This study |
| NE07-1 | M55 | 136 217 223 319 381 | 73 94 173 204 263 309+C 315+C | | Mizoram, India | Chakma tribe | Our unpublished data |
| NE19-2 | M55 | 136 217 223 319 381 | 73 94 173 204 263 315+C 482 489 | | Mizoram, India | Chakma tribe | Our unpublished data |
| NE47-3 | M55 | 136 217 223 319 381 | 73 94 173 204 263 315+C | 3312-3680=3397 3540 | Mizoram, India | Ralt tribe | Our unpublished data |
| NE49-1 | M55 | 136 217 223 319 381 | 73 94 173 204 263 309+C 315+C 482 489 | | Mizoram, India | Ralt tribe | Our unpublished data |
| Melayu* | M55 | 136 217 223 319 381 | | | Sumatrans Malays | Melayu | 23 |
| Burman507 | M55 | 148 217 223 319 381 519 | 73 94 173 204 263 309+C 315+C 482 489 | 9223-9650=9447 9540 | Sagaing, Myanmar | Naga | This study |
| Burman504* | M55 | 148 217 223 319 381 519 | 73 94 173 204 263 309+C 315+C 482 489 | | Sagaing, Myanmar | Naga | This study |
| LisuDI1* | M55 | 172 217 223 291 319 381 | 73 94 173 263 315+C 373 482 489 | | Dehong , China | Lisu | Our unpublished data |
| LisuDL2 | M55 | 172 217 223 291 319 381 | 73 94 173 263 315+C 373 482 489 | | Dehong , China | Lisu | Our unpublished data |
| LisuDY2 | M55 | 172 217 223 291 319 381 | 73 94 173 263 315+C 373 482 489 | | Dehong , China | Lisu | Our unpublished data |
| Lisu | M55 | 172 217 223 319 381 | | 16024–16383 | Chiang Rai, Thailand | | 24 |
| Lisu | M55 | 172 217 223 319 381 | | 16024–16383 | Chiang Rai, Thailand | | 24 |
| Lisu | M55 | 172 217 223 319 381 | | 16024–16383 | Mae Hong Son, Thailand | | 24 |
| Lisu | M55 | 172 217 223 319 381 | | 16024–16383 | Mae Hong Son, Thailand | | 24 |
| Lisu | M55 | 172 217 223 319 381 | | 16024–16383 | Mae Hong Son, Thailand | | 24 |
| Deang OT3* | M55 | 172 217 223 319 381 | 73 94 173 263 315+C 373 482 489 | 8247-9235=8701 8860 -10397AluI | Dehong , China | Deang | Our unpublished data |
| LisuDU1* | M55 | 172 217 223 319 381 | 73 94 173 263 315+C 373 482 489 | | Dehong , China | Lisu | Our unpublished data |
| TH2A10 | M55 | 217 223 319 381C 519 | 73 94 173 204 263 309+2C 315+C 455+T 482 489 | 4491 10400 | Chiang Mai, Thailand | | 17 |
| TH2H10 | M55 | 217 223 319 381C 519 | 73 94 173 204 263 309+2C315+C 482 489 | 4491 10400 | Chiang Mai, Thailand | | 17 |
| GX-Jing120 | M55 | 217 223 319 365 381 | 73 94 182 263 309+CC 315+C | 12406HincII+ 13262AluI- 14465AccI- | Guangxi, China | Jing | Our unpublished data |
| Mussur | M55 | 217 223 319 381 | | | Chiang Mai, Thailand | | 25 |
| Mussur | M55 | 217 223 319 381 | | | Chiang Mai, Thailand | Mussur | 25 |
| Mon* | M55 | 136 217 223 319 381 | | | Rangoon, Myanmar | Mon | Familytree DNA |
| Burman419* | M55 | 217 223 319 381 519 | 73 94 173 204 263 315+C 482 489 | 9241-9570=9447 9540 | Sagaing, Myanmar | Naga | This study |
| Burman432 | M55 | 217 223 319 381 519 | 73 94 173 204 263 315+C 482 489 | 9223-9660=9447 9540 | Sagaing, Myanmar | Naga | This study |
| JingpoAS1* | M55 | 217 223 319 381 519 | 73 94 173 204 263 309+C 315+C | +12406HpaI-663HaeIII+10397AluI | Dehong , China | Jingpo | Our unpublished data |
| JingpoSB1 | M55 | 217 223 319 381 519 | 73 94 173 204 263 309+C 315+C | +12406HpaI-663HaeIII-3391HaeIII+10397AluI | Dehong , China | Jingpo | Our unpublished data |
| Burman459* | M54 | 188 192 223 304 311 519 | 73 146 263 315+C 489 | | Sagaing , Myanmar | Naga | This study |
| RV49 | M54 | 188 192 223 304 311 | | 12007 | India | Rabha | Our unpublished data |
| Thai329* | M54 | 093 188 189 192 223 304 519 | 73 263 315+C 489 | | Thailand | | 8 |
| XEB012* | M54 | 145 188N 189N 192N 223 293 304 519 | 73 263 489 | | Tibet, China | | 26 |
| XEB042* | M54 | 145 188N 189N 192N 223 293 304 519 | 73 263 489 | | Tibet, China | | 26 |
| Tam24 | M54 | 129 145 187 188 192 230 278 293 304 | 73 146 152 195 247 263 309+C 315+C | | Nepal | TB | 27 |
| Tam6 | M54 | 129 145 187 188 192 230 278 293 304 | 73 146 152 195 247 263 309+C 315+C | | Nepal | TB | 27 |
| Burman716 | M54 | 145 172 188d 192+T 223 293 304 318 | 73 263 315+C 489 | | Bago , Myanmar | Burmans | This study |
| Bai01-05 | M54 | 188 189 192 223 239 304 | | | Yunnan, China | Bai | 28 |
| YXLPCT375 | M54 | 188 189 192 223 239 304 519 | 73 263 309+C 315+C 489 523-524d 750 | | Yunnan, China | Han | 5 |
| H-Yunnan26 | M54 | 188 189 192 223 304 | | | Yunnan, China | Han | 29 |
| LP011* | M54 | 188 189 192 223 304 324 519 | 73 263 309+CC 315+C 489 750 | | Yunnan, China | Han | 5 |

| Sample name | HG | HVS-I (16000+) | HVS II | Some Coding-Region Polymorphisms | Location | Nation | Reference |
|---|---|---|---|---|---|---|---|
| LP080* | M54 | 188 189 192 223 304 519 | 73 262 263 309+C 315+C 489 750 | | Yunnan, China | Han | 5 |
| LP482 | M54 | 188 189 192 223 304 519 | 73 263 309+C 315+C 489 750 | | Yunnan, China | Han | 5 |
| YXLPCT187* | M54 | 188 189 192 223 304 519 | 73 263 309+C 315+C 489 750 | | Yunnan, China | Han | 5 |
| YXLPCT598* | M54 | 188 189 192 223 304 519 | 73 263 309+CC 315+C 489 750 | | Yunnan, China | Han | 5 |
| YXLPCT620 | M54 | 188 189 192 223 304 519 | 73 263 309+C 315+C 489 750 | | Yunnan, China | Han | 5 |
| LP366* | M54 | 188 189 192 223 304 519 | 73 263 309+CC 315+C 489 750 | | Yunnan, China | Han | 5 |
| LP533* | M54 | 188 189 192 223 304 519 | 73 180 263 309+CC 315+C 489 750 | | Yunnan, China | Han | 5 |
| YUN-HAN-268 | M54 | 188d 192+C 223 304 519 | 73 180 263 309+CC 315+C | | Yunnan, China | Han | Our unpublished data |
| YUN-HAN-050 | M54 | 188d 192+T 223 304 519 | 73 263 309+C 315+C | | Yunnan, China | Han | Our unpublished data |
| YXLPCT770* | M54 | 188 189 192 223 519 | 73 207 263 291+A 309+C 315+C 489 750 | | Yunnan, China | Han | 5 |
| LisuBD1* | M84 | 223 258C 261 262+C 272 311 519 | 73 150 152 185 263 279 309+C 315+C 489 | +5176AluI-4831HhaI-9820HinfI-3391HaeIII+10397AluI | Yunnan, China | Lisu | Our unpublished data |
| LisuBF2 | M84 | 223 258C 261 262+C 272 311 519 | 73 150 152 185R 263 279 309+C 315+C 489 | +5176AluI-4831HhaI +9820HinfI-3391HaeIII+10397AluI | Yunnan, China | Lisu | Our unpublished data |
| LisuBL1* | M84 | 223 258C 261 262+C 272 311 519 | 73 150 152 185 263 279 309+C 315+C 489 | +5176AluI-4831HhaI -9820HinfI-3391HaeIII+10397AluI | Yunnan, China | Lisu | Our unpublished data |
| LisuBM1 | M84 | 223 258C 262+C 272 311 519 | 73 150 152 185 263 279 309+C 315+C 489 | +5176AluI-4831HhaI -9820HinfI-3391HaeIII+10397AluI | Yunnan, China | Lisu | Our unpublished data |
| Burman233* | M84 | 223 258C 262+C 272 519 | 73 185 189 263 315+C 489 | | Chin, Myanmar | Chin | This study |
| Naga16 | M84 | 223 258C 272 | | | Nagaland India, India | Naga | 15 |
| Naga34 | M84 | 223 258C 272 | | | Nagaland India, India | Naga | 15 |
| Naga58 | M84 | 223 258C 272 | | | Nagaland India, India | Naga | 15 |
| Burman092* | M84 | 223 258C 272 519 | 73 185 263 309+C 315+C 489 | 14079-14596=14110 | Sagaing , Myanmar | Burmans | This study |
| Burman094 | M84 | 223 258C 272 519 | 73 185 263 309+C 315+C | 14076-14604=14110 | Sagaing , Myanmar | Burmans | This study |
| Burman171* | M84 | 223 258C 272 519 | 73 185 263 315+C 456 489 522-523d | 14079-14595=14110 | Chin, Myanmar | Chin | This study |
| NE48-2 | M84 | 223 258C 272 519 | 73 185 189 263 315+C 489 | 4561-5074=4769 4907 | Mizoram, India | Ralt tribe | Our unpublished data |
| NE49-4 | M84 | 223 258C 272 519 | 73 185 189 263 315+C 489 | | Mizoram, India | Ralt tribe | Our unpublished data |
| Naga3 | M84 | 223 258C 272 295 | | | Nagaland India, India | Naga | 15 |
| JingpoSC1* | M84 | 223 258C 272 311 519 | 73 150 152 185 263 279 315+C | +5176AluI-4831HhaI | Dehong , China | Jingpo | Our unpublished data |
| NE19-3 | M84 | 093 223 258d 263 272 519 | 73 185 263 309+C 315+C | 4508-5074=4769 4907 | Mizoram, India | Chakma tribe | Our unpublished data |
| Burman131* | M84 | 093 223 258d 272 519 | 73 185 188 195 263 315+C | | Chin, Myanmar | Chin | This study |
| Burman168 | M84 | 093 223 258d 272 519 | 73 185 188 195 263 315+C 489 | 1592-1929=1719 1809 | Chin, Myanmar | Chin | This study |
| Burman250* | M84 | 093 223 258d 272 519 | 73 185 263 315+C 489 | | Chin, Myanmar | Chin | This study |
| Burman366 | M84 | 093 223 258d 272 519 | 73 185 263 315+C 489 | 1532-1882=1719 1809 | Chin, Myanmar | Chin | This study |
| Burman405 | M84 | 093 223 258d 272 519 | 73 185 263 315+C 489 | 1518-2018=1719 1809 | Chin, Myanmar | Chin | This study |
| Burman649 | M84 | 093 223 258d 272 519 | 73 185 188 195 263 315+C 489 | 1532-1871=1719 1809 | Magway, Myanmar | Chin | This study |
| NE22-1 | M84 | 093 223 258d 272 519 | 73 185 263 309+C 315+C 489 | 4564-5074=4769 4907 | Mizoram, India | Chakma tribe | Our unpublished data |
| Burman592* | M84 | 129 223 258d 272 519 | 73 185 195 263 315+C 489 | 1513-2000=1719 1809 | Magway, Myanmar | Burmans | This study |
| Burman652 | M84 | 183d 223 224 258d 272 519 | 73 146 185 263 309+C 315+C 489 | 1502-2000=1719 | Bago, Myanmar | Burmans | This study |
| Burman012* | M84 | 183d 223 224 258d 272 519 | 73 146 185 263 309+C 315+C | | Magway, Myanmar | Rakhine | This study |
| Burman645* | M84 | 193 223 258d 272 519 | 73 185 263 315+C 489 | 1530-1860=1719 1809 | Magway, Myanmar | Chin | This study |
| Burman170* | M84 | 223 247 258d 272 519 | 73 185 263 315+C 456 489 522-523d | 1626-1962=rCRS | Chin, Myanmar | Chin | This study |
| NE02-1 | M84 | 223 258d 272 362 519 | 73 185 263 315+C | 4702-5074=4769 4907 | Mizoram, India | Chakma tribe | Our unpublished data |
| NE14-2 | M84 | 223 258d 272 362 519 | 73 185 263 315+C 489 | 4523-5251/15628-16000=4769 4907 | Mizoram, India | Chakma tribe | Our unpublished data |
| NE24-2 | M84 | 223 258d 272 362 519 | 73 185 263 315+C 489 | 4554-5074=4769 4907 | Mizoram, India | Chakma tribe | Our unpublished data |
| NE40-3 | M84 | 223 258d 272 362 519 | 73 185 263 315+C 489 | 4623-5074=4769 4907 | Mizoram, India | Ralt tribe | Our unpublished data |
| NE09-4 | M84 | 223 258d 272 519 | 73 185 263 315+C | 4520-5285/15628-16000=4769 4907 5021 | Mizoram, India | Chakma tribe | Our unpublished data |
| Burman196 | M84 | 223 258d 272 519 | 73 185 263 309+C 315+C | 14077-14705=14110 | Sagaing, Myanmar | Chin | This study |
| Burman125 | M84 | 223 258d 272 519 | 73 185 263 315+C 489 | 11033-11817=11191 11719 | Chin, Myanmar | Chin | This study |

| Sample name | HG | HVS-I (16000+) | HVS II | Some Coding-Region Polymorphisms | Location | Nation | Reference |
|---|---|---|---|---|---|---|---|
| Burman136 | M84 | 223 258d 272 519 | 73 185 263 315+C 489 | 1529-1839=1719 | Chin, Myanmar | Chin | This study |
| Burman138 | M84 | 223 258d 272 519 | 73 185 263 315+C 489 | 1634-1762=1719 | Chin, Myanmar | Chin | This study |
| Burman139 | M84 | 223 258d 272 519 | 73 185 263 315+C 489 | 1703-1731=1719 | Chin, Myanmar | Chin | This study |
| Burman150 | M84 | 223 258d 272 519 | 73 185 263 315+C 489 | 1502-2000=1719 1809 | Chin, Myanmar | Chin | This study |
| Burman151 | M84 | 223 258d 272 519 | 73 185 263 315+C | 1513-1900=1719 1809 | Chin, Myanmar | Chin | This study |
| Burman157 | M84 | 223 258d 272 519 | 73 185 263 315+C | 1624-1764=1719 | Chin, Myanmar | Chin | This study |
| Burman158 | M84 | 223 258d 272 519 | 73 185 263 315+C | 1690-1856=1719 1809 | Chin, Myanmar | Chin | This study |
| Burman159 | M84 | 223 258d 272 519 | 73 185 263 315+C 489 | | Chin, Myanmar | Chin | This study |
| Burman165 | M84 | 223 258d 272 519 | 73 185 263 315+C 489 | 1624-1936=1719 1809 | Chin, Myanmar | Chin | This study |
| Burman166 | M84 | 223 258d 272 519 | 73 185 263 315+C | 1563-1897=1719 1809 | Chin, Myanmar | Chin | This study |
| Burman235* | M84 | 223 258d 272 519 | 73 185 189 199 263 315+C 489 | 1660-1968=1719 1809 | Chin, Myanmar | Chin | This study |
| Burman342 | M84 | 223 258d 272 519 | 73 185 263 315+C 489 | 1538-1892=1719 1809 | Chin, Myanmar | Chin | This study |
| Burman640 | M84 | 223 258d 272 519 | 73 185 263 315+C 489 | | Magway, Myanmar | Chin | This study |
| Burman641 | M84 | 223 258d 272 519 | 73 185 263 315+C 489 | 1516-1881=1719 1809 | Magway, Myanmar | Chin | This study |
| NE20-1 | M84 | 223 258d 272 519 | 73 185 263 315+C 489 | 4743-5074=4769 4907 | Mizoram, India | Chakma tribe | Our unpublished data |
| NE35-1 | M84 | 223 258d 272 519 | 73 185 239 263 315+C 489 | 4686-5074=4769 4907 | Mizoram, India | Ralt tribe | Our unpublished data |
| NE42-2 | M84 | 223 258d 272 519 | 73 185 263 315+C 489 | 4670-5074=4769 4907 | Mizoram, India | Ralt tribe | Our unpublished data |
| NE43-1 | M84 | 223 258d 272 519 | 73 185 263 309+C 315+C | 4500-5074=4769 4907 | Mizoram, India | Ralt tribe | Our unpublished data |
| NE55-4 | M84 | 223 258d 272 519 | 73 185 263 315+C 489 | | Mizoram, India | Ralt tribe | Our unpublished data |
| NE56-1 | M84 | 223 258d 272 519 | 73 185 189 263 315+C 489 | 4675-5074=4769 4907 | Mizoram, India | Kuki tribe | Our unpublished data |
| NE62-3 | M84 | 223 258d 272 519 | 73 185 189 263 315+C 489 563+A 573+C | 4700-5081=4769 4907 | Mizoram, India | Kuki tribe | Our unpublished data |
| NE64-2 | M84 | 223 258d 272 519 | 73 185 263 315+C 489 | | Mizoram, India | Kuki tribe | Our unpublished data |
| MMR144 | M84 | 223 258d 272 519 | 73 150 185 263 315+C 489 | | Mon State, Myanmar | Bamar | 7 |
| Burman515 | M63 | 223 399 488 497 519 | 73 214 263 309+C 315+C 489 | 11756-12313=12007 12239 | Magway, Myanmar | Burmans | This study |
| Burman516 | M63 | 223 399 488 497 519 | 73 214 263 309+C 315+C 489 | 11756-12268=12007 12239 | Magway, Myanmar | Burmans | This study |
| Burman534* | M63 | 223 399 488 497 519 | 73 214 263 309+C 315+C 489 | | Magway, Myanmar | Burmans | This study |
| Burman601 | M63 | 223 399 488 497 519 | 73 214 263 309+C 315+C 489 | 11726-12246=12007 12236 | Magway, Myanmar | Burmans | This study |
| A196 | M63 | 126 223 318T 488 497 | 73 214 263 | | Uttar Pradesh, India | Bhargava | Our unpublished data |
| BN13/2 | M63 | 488 497 | 73 214 263 | | Bangladesh | | Our unpublished data |
| Bi32* | M63 | 223 488 497 519 527 | | | Madhya Pradesh, India | | 30 |
| MA60* | M63 | 172 192 223 248 519 | | | India | | 16 |
| MA110* | M63 | 172 192 223 519 | | | India | | 16 |
| MA78* | M63 | 172 192 223 248 519 | | | India | | 16 |
| MA79* | M63 | 172 192 223 248 519 | | | India | | 16 |
| MA84* | M63 | 172 192 223 248 519 | | | India | | 16 |
| MA106* | M63 | 172 192 223 248 519 | | | India | | 16 |

Note: Suffixe "*" means the mtDNA of the sample has been completely sequenced.

**References for Table S8**


1. Zhang, X. M. *et al.* Analysis of mitochondrial genome diversity identifies new and ancient maternal lineages in Cambodian aborigines. Nat. Commun. **4,** (2013).

2. Scholes, C. *et al.* Genetic Diversity and Evidence for Population Admixture in Batak Negritos from Palawan. Am. J. Phys. Anthropol. **146,** 62-72 (2011).

3. Kivisild, T. *et al.* The Role of Selection in the Evolution of Human Mitochondrial Genomes. Genetics **172,** 373-387 (2006).

4. Zhao, M. *et al.* Mitochondrial genome evidence reveals successful Late Paleolithic settlement on the Tibetan Plateau. Proc. Natl. Acad. Sci. USA **106,** 21230-21235 (2009).

5. van Oven M, Kayser M. Updated comprehensive phylogenetic tree of global human mitochondrial DNA variation. Hum. Mutat. **30,** E386-394 (2009).

6. Wen B, *et al.* Genetic evidence supports demic diffusion of Han culture. Nature **431,** 302-305 (2004).

7. Summerer, M. *et al.* Large-scale mitochondrial DNA analysis in Southeast Asia reveals evolutionary effects of cultural isolation in the multi-ethnic population of Myanmar. BMC Evol. Biol. **14,** 17 (2014).

8. Pradutkanchana, S., Ishida, T. & Kimura, R. Mitochondrial diversity of the sea nomads of Thailand. Unpublished (2011).

9. Yao, Y. G. *et al.* Genetic relationship of Chinese ethnic populations revealed by mtDNA sequence diversity. Am. J. Phys. Anthropol. **118,** 63-76 (2002).

10. Peng, M. S. *et al.* Tracing the Austronesian Footprint in Mainland Southeast Asia: A Perspective from Mitochondrial DNA. Mol. Biol. Evol. **27,** 2417-2430 (2010).

11. Wang, W. Z. *et al.* Tracing the origins of Hakka and Chaoshanese by mitochondrial DNA analysis. Am. J. Phys. Anthropol. **141,** 124-130 (2010).

12. Tabbada, K. A. *et al.* Philippine Mitochondrial DNA Diversity: A Populated Viaduct between Taiwan and Indonesia? Mol. Biol. Evol. **27,** 21-31 (2010).

13. Gazi, N. N. *et al.* Genetic Structure of Tibeto-Burman Populations of Bangladesh: Evaluating the Gene Flow along the Sides of Bay-of-Bengal. PLoS ONE **8,** e75064 (2013).

14. Hill, C. *et al.* A mitochondrial stratigraphy for island southeast Asia. Am. J. Hum. Genet. **80,** 29-43 (2007).

15. Cordaux, R. *et al.* Mitochondrial DNA analysis reveals diverse histories of tribal populations from India. Eur. J. Hum. Genet. **11,** 253-264 (2003).

16. Chandrasekar, A. *et al.* Updating phylogeny of mitochondrial DNA macrohaplogroup M in India: dispersal of modern human in South Asian corridor. PLoS ONE **4,** e7447 (2009).

17. Zimmermann, B. *et al.* Forensic and phylogeographic characterization of mtDNA lineages from northern Thailand (Chiang Mai). Int. J. Legal. Med. **123,** 495-501 (2009).

18. Eaaswarkhanth, M. *et al.* Traces of sub-Saharan and Middle Eastern lineages in Indian Muslim populations. Eur. J. Hum. Genet. **18,** 354-363 (2009).

19. Ji, F. Y. *et al.* Mitochondrial DNA variant associated with Leber hereditary optic neuropathy and high-altitude Tibetans. Proc. Natl. Acad. Sci. USA **109,** 7391-7396 (2012).

20. Fornarino, S. *et al.* Mitochondrial and Y-chromosome diversity of the Tharus (Nepal): a reservoir of genetic variation. BMC Evol. Biol. **9,** 154-171 (2009).

21. Kong, Q. P. *et al.* Large-scale mtDNA screening reveals a surprising matrilineal complexity in east asia and its implications to the peopling of the region. Mol. Biol. Evol. **28,** 513-522 (2011).

22. Maruyama, S., Minaguchi, K. & Saitou, N. Sequence polymorphisms of the mitochondrial DNA control region and phylogenetic analysis of mtDNA lineages in the Japanese population. Int. J. Legal. Med. **117,** 218-225 (2003).

23. Hill, C. *et al.* Phylogeography and ethnogenesis of aboriginal Southeast Asians. Mol. Biol. Evol. **23,** 2480-2491 (2006).

24. Oota, H., Settheetham-Ishida, W., Tiwawech, D., Ishida, T. & Stoneking, M. Human mtDNA and Y-chromosome variation is correlated with matrilocal versus patrilocal residence. Nat. Genet. **29,** 20-21 (2001).

25. Fucharoen, G., Fucharoen, S. & Horai, S. Mitochondrial DNA polymorphisms in Thailand. J. Hum. Genet. **46,** 115-125 (2001).

26. Kang, L. L. *et al.* MtDNA lineage expansions in Sherpa population suggest adaptive evolution in Tibetan highlands. Mol. Biol. Evol. **30,** 2579-2587 (2013).

27. Gayden, T. *et al.* The Himalayas: Barrier and conduit for gene flow. Am. J. Phys. Anthropol. **151,** 169-182 (2013).

28. Wen, B. *et al.* Analyses of genetic structure of Tibeto-Burman populations reveals sex-biased admixture in southern Tibeto-Burmans. Am. J. Hum. Genet. **74,** 856-865 (2004).

29. Wen, B. *et al.* Genetic evidence supports demic diffusion of Han culture. Nature **431,** 302-305 (2004).

30. Sharma, G. *et al.* Genetic Affinities of the Central Indian Tribal Populations. PLoS ONE **7,** e32546 (2012).
